# Identification of candidate biomarkers and pathways associated with type 1 diabetes mellitus using bioinformatics analysis

**DOI:** 10.1038/s41598-022-13291-1

**Published:** 2022-06-01

**Authors:** Madhu Pujar, Basavaraj Vastrad, Satish Kavatagimath, Chanabasayya Vastrad, Shivakumar Kotturshetti

**Affiliations:** 1 Department of Pediatrics, J J M Medical College, Davangere, Karnataka 577004 India; 2 Department of Pharmaceutical Chemistry, K.L.E. College of Pharmacy, Gadag, Karnataka 582101 India; 3 Department of Pharmacognosy, K.L.E. College of Pharmacy, Belagavi, Karnataka 590010 India; 4 Biostatistics and Bioinformatics, Chanabasava Nilaya, Bharthinagar, Dharwad, Karnataka 580001 India

**Keywords:** Computational biology and bioinformatics, Biomarkers, Endocrine system and metabolic diseases, Molecular medicine

## Abstract

Type 1 diabetes mellitus (T1DM) is a metabolic disorder for which the underlying molecular mechanisms remain largely unclear. This investigation aimed to elucidate essential candidate genes and pathways in T1DM by integrated bioinformatics analysis. In this study, differentially expressed genes (DEGs) were analyzed using DESeq2 of R package from GSE162689 of the Gene Expression Omnibus (GEO). Gene ontology (GO) enrichment analysis, REACTOME pathway enrichment analysis, and construction and analysis of protein–protein interaction (PPI) network, modules, miRNA-hub gene regulatory network and TF-hub gene regulatory network, and validation of hub genes were performed. A total of 952 DEGs (477 up regulated and 475 down regulated genes) were identified in T1DM. GO and REACTOME enrichment result results showed that DEGs mainly enriched in multicellular organism development, detection of stimulus, diseases of signal transduction by growth factor receptors and second messengers, and olfactory signaling pathway. The top hub genes such as MYC, EGFR, LNX1, YBX1, HSP90AA1, ESR1, FN1, TK1, ANLN and SMAD9 were screened out as the critical genes among the DEGs from the PPI network, modules, miRNA-hub gene regulatory network and TF-hub gene regulatory network. Receiver operating characteristic curve (ROC) analysis confirmed that these genes were significantly associated with T1DM. In conclusion, the identified DEGs, particularly the hub genes, strengthen the understanding of the advancement and progression of T1DM, and certain genes might be used as candidate target molecules to diagnose, monitor and treat T1DM.

## Introduction

Type 1 diabetes mellitus (T1DM) is chronic autoimmune diabetes characterized by autoimmune mediated destruction of pancreatic beta cells^1^. T1DM is most generally identified in children and adolescents^2^. Epidemiological studies have shown that the incidence of T1DM has been increasing by 2–5% globally^3^. T1DM is a complex disease affected by numerous environmental factors, genetic factors and their interactions^4,5^. Several T1DM associated complications include cardiovascular disease^6^, hypertension^7^, diabetic retinopathy^8^, diabetic nephropathy^9^, diabetic neuropathy^10^, obesity^11^ and cognitive impairment^12^. Therefore, it is crucial to understand the precise molecular mechanisms associated in the progression of T1DM and thus establish effective diagnostic, prognostics and therapeutic strategies.

Although the remarkable improvement is achieved in the treatment of T1DM is insulin therapy^13^, the long-term survival rates of T1DM still remain low worldwide. One of the major reasons is that most patients with T1DM were diagnosed at advanced stages. It is crucial to find out novel diagnostic biomarkers, prognostic biomarkers and therapeutic targets for the early diagnosis, prognosis and timely treatment of T1DM. Therefore, it is still urgent to further explore the exact molecular mechanisms of the development of T1DM. At present, several genes and signaling pathway are identified; for example vitamin D receptor (VDR)^14^, HLA-B and HLA-A^15^, HLA-DQ^16^, HLA‐DQB1, HLA‐DQA1 and HLA‐DRB1^17^, IDDM2^18^, CaMKII/NF-κB/TGF-β1 and PPAR-γ signaling pathway^19^, Keap1/Nrf2 signaling pathway^20^, HIF-1/VEGF pathway^21^, NLRP3 and NLRP1 inflammasomes signaling pathway^22^ and NO/cGMP signaling pathway^23^. Therefore, it is crucial to examine the accurate molecular targets included in occurrence and advancement of T1DM, in order to make a contribution to the diagnosis and treatment of T1DM.

Next generation sequencing (NGS) platform for gene expression analysis have been increasingly recognized as approaches with significant clinical value in areas such as molecular diagnosis, prognostic prediction and identification of novel therapeutic targets^24^. In recent years, NGS data analysis has been effective in detecting the advancement of T1DM, and even in screening biomarkers for T1DM prognosis, diagnosis and therapy. We therefore used an NGS dataset to investigate the molecular pathogenesis of T1DM.

In the present investigation, we selected NGS dataset GSE162689^25^, from Gene Expression Omnibus database (GEO) (http://www.ncbi.nlm.nih.gov/geo/)^26^ and used the DESeq2 package in R software to screen DEGs. We performed subsequent bioinformatics analysis, including gene ontology (GO) enrichment and REACTOME pathway enrichment analysis, and construction and analysis of protein–protein interaction (PPI) network, module analysis, construction and analysis of miRNA-hub gene regulatory network and TF-hub gene regulatory network. The hub genes were validated by receiver operating characteristic curve (ROC) analysis. This investigation might offer better insight into potential molecular mechanisms to examine preventive and therapeutic strategies.

## Materials and methods

### Data resources

NGS dataset of T1DM (GSE162689)^25^ was downloaded from the GEO database. The GSE162689 NGS data was composed of 27 T1DM samples and 32 normal control samples was based on the GPL24014 Ion Torrent S5 XL (Homo sapiens).

### Identification of DEGs

Differentially expressed genes (DEGs) between T1DM and normal control samples were identified by using the DESeq2 package in R language software^27^. DEGs were considered when an adjusted P < 0.05, and a |log2 fold change|> 0.63 for up regulated genes and |log2 fold change|< − 1.3 for down regulated genes. The adjusted P values, by employing Benjamini and Hochberg false discovery rate^28^, were aimed to correct the occurrence of false positive results. The DEGs were presented in volcano plot and heat map drawn using a plotting tool ggplot2 and gplots based on the R language.

### GO and REACTOME pathway enrichment analysis of DEGs

One online tool, g:Profiler (http://biit.cs.ut.ee/gprofiler/)^29^, was applied to carried out the functional annotation for DEGs. Gene Ontology (GO) (http://geneontology.org/)^30^ generally performs enrichment analysis of genomes. GO terms includes biological processes (BP), cellular components (CC) and molecular functions (MF) in the GO enrichment analysis. REACTOME (https://reactome.org/)^31^ is a comprehensive database of genomic, chemical, and systemic functional information. GO and pathway enrichment analyses were used to identify the significant GO terms and pathways. P < 0.05 was set as the cutoff criterion.

### Construction of the PPI network and module analysis

PPI network was established using the IntAct Molecular Interaction Database (https://www.ebi.ac.uk/intact/)^32^. To assess possible PPI correlations, previously identified DEGs were mapped to the IntAct database, followed by extraction of PPI pairs with a combined score > 0.4. Cytoscape 3.8.2 software (www.cytoscape.org/)^33^ was then employed to visualize the PPI network, and the Cytoscape plugin Network Analyzer was used to calculate the node degree^34^, betweenness centrality^35^, stress centrality^36^ and closeness centrality^37^ of each node in PPI network. Specifically, nodes with a higher node degree, betweenness centrality, stress centrality and closeness centrality were likely to play a more vital role in maintaining the stability of the entire network. The PEWCC1 (http://apps.cytoscape.org/apps/PEWCC1)^38^ plug-in was applied to analyze the modules in the PPI networks, with the default parameters (node score = 0.2, K-core ≧ 2, and max depth = 100).

### MiRNA-hub gene regulatory network construction

The miRNAs targeting the T1DM related were predicted using the miRNet database (https://www.mirnet.ca/)^39^, and those predicted by at least 14 databases (TarBase, miRTarBase, miRecords, miRanda, miR2Disease, HMDD, PhenomiR, SM2miR, PharmacomiR, EpimiR, starBase, TransmiR, ADmiRE, and TAM 2.0) were selected for constructing the miRNA-hub gene regulatory network by Cytoscape 3.8.2 software^33^.

### TF-hub gene regulatory network construction

The TFs targeting the T1DM related were predicted using the NetworkAnalyst database (https://www.networkanalyst.ca/)^40^, and those predicted by RegNetwork database was selected for constructing the TF-hub gene regulatory network by Cytoscape 3.8.2 software^33^.

### Validation of hub genes by receiver operating characteristic curve (ROC) analysis

A ROC curve analysis is an approach for visualizing, organizing and selecting classifiers based on their achievement of hub genes. A diagnostic test was firstly performed in order to estimate the diagnostic value of hub genes in T1DM. ROC curves were obtained by plotting the sensitivity, against the specificity using the R package “pROC”^41^. Area under the curve (AUC) was used to measure the accuracy of these diagnostic values of the hub genes An AUC > 0.9 determined that the model had a favorable fitting effect.

### Ethical approval

This article does not contain any studies with human participants or animals performed by any of the authors.

### Informed consent

No informed consent because this study does not contain human or animals participants.

## Results

### Identification of DEGs

**Figure 1:**
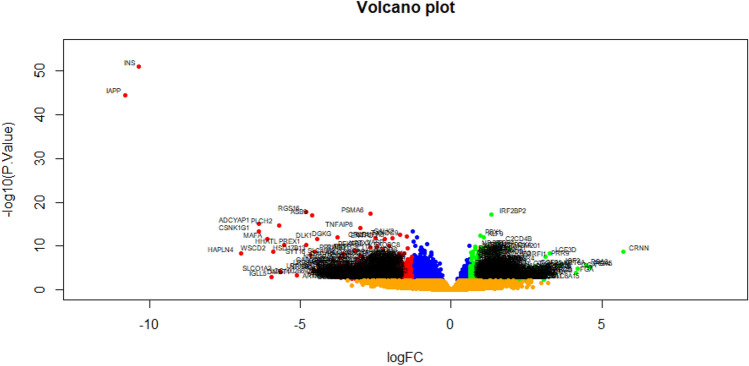
Volcano plot of differentially expressed genes. Genes with a significant change of more than two-fold were selected. Green dot represented up regulated significant genes and red dot represented down regulated significant genes.

**Figure 2:**
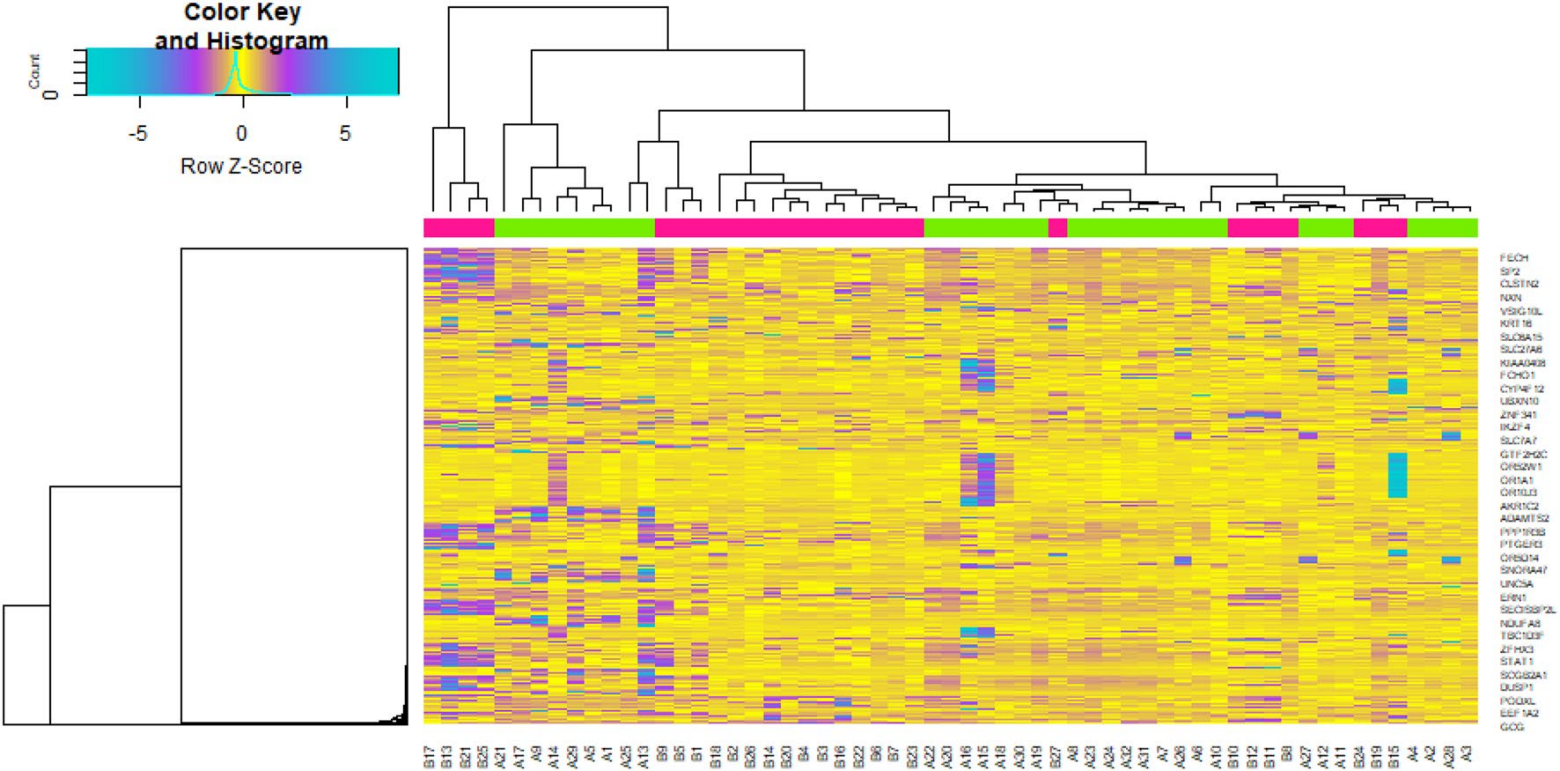
Heat map of differentially expressed genes. Legend on the top left indicate log fold change of genes. (A1–A32 = normal control samples; B1–B27 = T1DM samples).

On the basis of the cut‐off criteria, DEGs in GEO dataset was identified between T1DN and normal control samples (Supplementary Table S1). There were 952 DEGs, including 477 up regulated and 475 down regulated genes in GSE162689 with the threshold of adjusted P < 0.05, and a |log2 fold change|> 0.63 for up regulated genes and |log2 fold change|< − 1.3 for down regulated genes. Volcano plots (Fig. 1) showed the correlation of all DEGs from the NGS data. Heat map of the up regulated and down regulated genes were indicated in Fig. 2.

### GO and REACTOME pathway enrichment analysis of DEGs

To characterize the functional roles of the above DEGs, we used GO (Table 1) and REACTOME pathway (Table 2) enrichment analyses. The BP category of the GO analysis results showed that up regulated genes were significantly enriched in multicellular organism development and nitrogen compound metabolic process. For CC, these up regulated were enriched in membrane-enclosed lumen and nuclear lumen. Moreover, up regulated genes were significantly enriched in protein binding and transcription regulator activity in the MF categories. In addition, the most significantly enriched GO terms for down regulated genes were detection of stimulus and multicellular organismal process (BP), cell periphery and plasma membrane (CC), and transmembrane signaling receptor activity and molecular transducer activity (MF). According to REACTOME pathway enrichment analysis, up regulated genes were significantly enriched in diseases of signal transduction by growth factor receptors and second messengers and formation of the cornified envelope. Down regulated genes were enriched in olfactory signaling pathway and sensory perception.

**Table 1:** The enriched GO terms of the up and down regulated differentially expressed genes.

GO ID	CATEGORY	GO name	Adjusted p value	Negative log10 of adjusted p value	Gene count	Gene
**Up regulated genes**						
GO:0007275	BP	Multicellular organism development	1.03027E−05	4.987047489	192	IGF2, KRT6A, LCE3D, SPRR3, FLG, SPRR2D, SPRR1B, SLITRK6, FGF21, TBX22, CALCR, SPRR1A, USH2A, OTX2, DCC, KCP, NOG, STAR, KRT16, IL1RN, SHISA2, AQP5, SYNDIG1, TFAP2C, ERRFI1, PLP1, ALOX12, KRT13, SPRR2E, TENM1, PEMT, LY6H, FAP, EYA4, LCE3E, EGR1, CSGALNACT1, MAL, MMP16, PTGER4, COL6A3, OAS2, ETV4, MAOB, GPC6, SOCS5, BCL9, POU6F2, BTG2, PRR15, VEGFC, FAM20C, MPZ, TMEM176A, KLK13, RPS6, ID3, ALOXE3, DDIT4, IRF2BP2, KLF15, EGR3, RNF165, LTBP4, CREB3L1, EMX1, KLF3, ZFP36, QDPR, ETV5, KL, GADD45B, NXN, TLE3, HEYL, HRAS, GLI3, SFTPD, MYC, IGSF8, DMD, FKBP8, DBP, MTCH1, KLF10, PODXL, BVES, MNT, LSR, CEL, FOSL2, WASF1, PAPSS2, NR1D1, DUSP4, SNX19, FOXN3, IL6ST, IL6R, PCDHB6, KLF6, ZFP36L1, PBX1, SH3RF1, CNTN3, NHEJ1, BOC, STAT1, DUSP1, CLSTN2, DIP2B, MYADM, PCDHB11, APOD, NR0B2, NHS, SYVN1, TCF7L2, DLL4, NNMT, SMAD1, TP53, PCDH18, SUN2, SOS1, PRKACA, EGFR, ETS1, PIK3CA, TSHZ1, GCNT2, PKN1, TNFRSF1A, ELOVL1, NCSTN, MFAP2, YBX1, PLCE1, NDST1, KCNJ8, GAMT, DGCR2, TPI1, JMJD8, NES, WDTC1, MAML1, PCDHB4, NFIB, MAPK3, SLC23A2, FOXK1, CAMSAP3, ACVR2B, PLOD3, ZFHX3, SMURF1, PRMT6, PRKCSH, ETV6, GSK3A, WDR74, BCOR, NPAS2, USP1, RBM47, RNF43, TCOF1, NCL, METTL14, KCTD11, HSP90AA1, BTG1, GAB1, S1PR1, THRB, EDNRB, SYBU, CDNF, MEX3C, SIAH2, NFKBIA, GJA1, CD34, DEAF1, INSR, NR1D2, GADD45G, ZNRF3, MBOAT7, BSG, BTF3
GO:0006807	BP	Nitrogen compound metabolic process	8.09009E−05	4.092046627	292	CRNN, PGA5, CGA, FGA, IGF2, FGB, APOA5, SPRR3, FLG, SPRR1B, FGF21, FGG, TBX22, CALCR, PGC, SPRR1A, ABCB11, OTX2, MUC21, NOG, HSFX2, ZSCAN10, TFAP2C, ERRFI1, RUNX1T1, ALOX12, SPRR2E, PDK4, TENM1, HAO1, PEMT, FAP, EYA4, ZNF554, EGR1, CSGALNACT1, GCG, MMP16, ADAMTS8, COL6A3, MAPK4, OAS2, ETV4, MAOB, GPC6, SOCS5, GMNC, BCL9, PDK3, POU6F2, BTG2, A4GNT, CST6, VEGFC, FAM20C, USP27X, STK32B, KLK13, RPS6, ID3, PGAP2, ALOXE3, LSM11, BCAP31, DDIT4, DPP4, TYSND1, IRF2BP2, KLF15, EGR3, RNF165, PHLDA1, LTBP4, IKZF4, CREB3L1, A1CF, EMX1, NAT10, ACSL6, KLF3, ZFP36, QDPR, OAS1, ETV5, GADD45B, MAN1A1, NXN, TLE3, RPL8, HEYL, PER3, HRAS, GLI3, SFTPD, MYC, RPRD2, DMD, ZNF416, FKBP8, RPS28, DBP, MUC6, MTCH1, CHST10, KLF9, EIF4B, KLF10, GLTP, MNT, CEL, NUP205, FOSL2, ZNF362, PAPSS2, NR1D1, DUSP4, SMCR8, FECH, FOXN3, IL6ST, IL6R, ZNF581, KLF6, CPA1, ABCA1, ZFP36L1, PBX1, SH3RF1, URM1, ERN1, RPL18, RNF24, PRRC1, NHEJ1, PDK2, STAT1, HSPA2, DUSP1, DIP2B, FMOD, MYADM, APOD, GLS, FBXO32, SRM, DIRAS3, NR0B2, SYVN1, ARL6IP1, TCF7L2, TMUB1, MRPL37, DLL4, HELZ2, NNMT, SMAD1, TP53, ELK1, PTBP1, PRKACA, BACH2, CTSF, EGFR, ETS1, PIK3CA, ZNRF1, CMPK2, GUK1, TSHZ1, ZNF326, MED13L, GCNT2, SAE1, PKN1, TNFRSF1A, ELOVL1, TUT1, NCSTN, YBX1, PPP1R3B, PLCE1, NDST1, ATF6B, GAMT, TPI1, JMJD8, SECISBP2L, PRPF8, EIF3B, WDTC1, MAML1, CRNKL1, NFIB, MAPK3, RPL18A, CNDP2, SERTAD2, RCE1, FOXK1, ZNF646, ACVR2B, PLOD3, ZFHX3, SMURF1, AOX1, RPS15, PRMT6, TRIM24, PRKCSH, ETV6, GSK3A, WDR74, LARP6, BCOR, URB1, ZNF341, SGTA, STUB1, NPAS2, MAOA, USP1, RBM47, RNF43, SHISA5, TCOF1, NCL, FKBP5, RBM22, METTL14, PIGM, ATF7IP, POLR1D, UBAP2, UBE2E2, SPPL3, KCTD11, HSP90AA1, BTG1, S1PR1, THRB, RPL39, UTP14A, EDNRB, CRTC3, AK1, MED25, ERP29, RBM23, RNASEK, LPCAT3, CASC3, MTMR3, TNKS, SF3A1, ADPRHL1, MEX3C, SIAH2, ACD, NFKBIA, GJA1, RNPS1, ATP13A2, TXNDC5, MRPL49, SLC35C2, CD34, SQSTM1, DEAF1, INSR, RPL19, NR1D2, AGBL5, SP2, SLC2A4RG, MRPS18B, NEDD9, GADD45G, ZNRF3, MAST3, SNX12, BAZ2A, MBOAT7, GID8, DCPS, LIG3, SP4, KLF11, CIZ1, LNX1, RALB, BTF3, ZNF836
GO:0031974	CC	Membrane-enclosed lumen	0.000311835	3.506075034	175	PGA5, CGA, FGA, IGF2, FGB, APOA5, FGG, MUC21, STAR, ZSCAN10, TFAP2C, RUNX1T1, SLC7A14, PDK4, TENM1, HAO1, ZNF554, EGR1, GCG, MMP16, COL6A3, MAPK4, OAS2, ETV4, GPC6, BCL9, PDK3, VEGFC, FAM20C, RPS6, LSM11, TYSND1, IRF2BP2, KLF15, PHLDA1, IKZF4, A1CF, EMX1, NAT10, KLF3, OAS1, ETV5, GRIK5, GPR63, TLE3, HEYL, HRAS, GLI3, MYC, RPRD2, IRS1, RPS28, MUC6, KLF9, PODXL, MNT, NUP205, FOSL2, NR1D1, DUSP4, SMCR8, FECH, KLF6, PBX1, RPL18, ABCG2, NHEJ1, PDK2, SELENBP1, STAT1, HSPA2, FMOD, GLS, FBXO32, NR0B2, NHS, SYVN1, TCF7L2, TMUB1, MRPL37, HELZ2, SMAD1, TP53, ELK1, SUN2, PTBP1, PRKACA, BACH2, TOR2A, CTSF, EGFR, ETS1, CMPK2, C17ORF49, ZNF326, SAE1, PKN1, TUT1, YBX1, ME2, JMJD8, PRPF8, WDTC1, MAML1, CRNKL1, NFIB, MAPK3, CNDP2, SERTAD2, FOXK1, CAMSAP3, PLOD3, FAM193B, ZFHX3, SMURF1, RPS15, PRMT6, TRIM24, PRKCSH, ETV6, WDR74, BCOR, URB1, STUB1, NPAS2, USP1, SHISA5, TCOF1, NCL, FKBP5, RBM22, METTL14, MZT2B, ATF7IP, POLR1D, FIGN, HSP90AA1, BTG1, S1PR1, THRB, UTP14A, CRTC3, MED25, ERP29, CASC3, TNKS, SF3A1, CHID1, SIAH2, ACD, NFKBIA, ARHGAP17, GJA1, RNPS1, ATP13A2, TXNDC5, MRPL49, SLC35C2, SQSTM1, DEAF1, INSR, RPL19, NR1D2, SP2, SLC2A4RG, MRPS18B, NEDD9, BAZ2A, CCDC86, GID8, DCPS, LIG3, SP4, KLF11, CIZ1
GO:0031981	CC	nuclear lumen	0.004404749	2.356078816	139	ZSCAN10, TFAP2C, RUNX1T1, SLC7A14, TENM1, ZNF554, EGR1, MAPK4, OAS2, ETV4, BCL9, PDK3, RPS6, LSM11, IRF2BP2, KLF15, PHLDA1, IKZF4, A1CF, EMX1, NAT10, KLF3, OAS1, ETV5, GRIK5, GPR63, TLE3, HEYL, HRAS, GLI3, MYC, RPRD2, IRS1, RPS28, KLF9, PODXL, MNT, NUP205, FOSL2, NR1D1, DUSP4, SMCR8, KLF6, PBX1, RPL18, ABCG2, NHEJ1, PDK2, SELENBP1, STAT1, HSPA2, FBXO32, NR0B2, NHS, SYVN1, TCF7L2, TMUB1, HELZ2, SMAD1, TP53, ELK1, SUN2, PTBP1, PRKACA, BACH2, ETS1, CMPK2, C17ORF49, ZNF326, SAE1, PKN1, TUT1, YBX1, PRPF8, WDTC1, MAML1, CRNKL1, NFIB, MAPK3, CNDP2, SERTAD2, FOXK1, CAMSAP3, FAM193B, ZFHX3, SMURF1, RPS15, PRMT6, TRIM24, ETV6, WDR74, BCOR, URB1, STUB1, NPAS2, USP1, TCOF1, NCL, FKBP5, RBM22, METTL14, MZT2B, ATF7IP, POLR1D, FIGN, HSP90AA1, BTG1, S1PR1, THRB, UTP14A, CRTC3, MED25, CASC3, TNKS, SF3A1, SIAH2, ACD, NFKBIA, ARHGAP17, GJA1, RNPS1, SLC35C2, SQSTM1, DEAF1, INSR, RPL19, NR1D2, SP2, SLC2A4RG, MRPS18B, NEDD9, BAZ2A, CCDC86, GID8, DCPS, LIG3, SP4, KLF11, CIZ1
GO:0005515	MF	Protein binding	0.010406822	1.982681882	384	CRNN, CGA, FGA, IGF2, KRT6A, FGB, LCE3D, SLC6A15, APOA5, SPRR3, FLG, SLITRK6, FGF21, FGG, TBX22, CALCR, SPRR1A, ABCB11, USH2A, OTX2, HBM, DCC, POTED, KCP, RGPD3, NOG, STAR, KRT16, HSFX2, IL1RN, ZSCAN10, SHISA2, TACR1, AQP5, SYNDIG1, TFAP2C, ERRFI1, TMEM174, CNTNAP4, PLP1, RUNX1T1, ALOX12, KRT13, SLC7A14, SPRR2E, PDK4, TENM1, PRLHR, SLC22A11, PEMT, LY6H, TMEM236, FAP, EYA4, BTBD11, FBP2, LCE3E, ZNF554, EGR1, CSGALNACT1, MT2A, MAL, SLC38A4, GCG, ASIC1, ADAMTS8, PTGER4, TMEM201, COL6A3, MAPK4, OAS2, AQP7, SLC25A48, ETV4, MAOB, GPC6, KCNJ2, MT1E, SOCS5, GMNC, BCL9, PDK3, ABCC9, POU6F2, BTG2, CST6, PRR15, VEGFC, FAM20C, C16ORF89, TMEM176A, KLK13, RPS6, ID3, PGAP2, ALOXE3, LSM11, BCAP31, DDIT4, DPP4, TYSND1, KLF15, RNF165, AQP8, SLC22A17, PHLDA1, FAM86B2, LTBP4, IKZF4, CREB3L1, A1CF, EMX1, NAT10, NETO2, ACSL6, KLF3, ZFP36, QDPR, OAS1, ETV5, GRIK5, KL, ZNF385C, GADD45B, SLC29A3, CGREF1, TLE3, RPL8, HEYL, PER3, HRAS, GLI3, WDR89, KIAA0408, SFTPD, MYC, TTI1, IFIT3, IGSF8, RPRD2, DMD, AQP12B, FKBP8, IRS1, KIAA1958, RPS28, TMEM140, MUC6, MTCH1, ABHD15, EIF4B, KLF10, PODXL, BVES, GLTP, MNT, CEL, NUP205, FOSL2, SLC1A5, WASF1, PAPSS2, TMCO4, NR1D1, DUSP4, SNX19, SMCR8, FECH, FOXN3, IL6ST, IL6R, PCDHB6, ZNF581, KLF6, CPA1, ABCA1, ZFP36L1, PBX1, DYSF, SH3RF1, PLEKHG6, URM1, ERN1, RPL18, CNTN3, CYGB, RNF24, ABCG2, PRRC1, NHEJ1, PDK2, BOC, SELENBP1, STAT1, HSPA2, DUSP1, DIP2B, FMOD, MYADM, ARPC1A, KCTD12, APOD, GLS, FBXO32, SRM, DIRAS3, NR0B2, SYVN1, ARL6IP1, TCF7L2, TMUB1, DHRS11, DLL4, HELZ2, SMAD1, TP53, PCDH18, ELK1, SUN2, TMEM150A, PTBP1, SOS1, PRKACA, BACH2, TOR2A, EGFR, ETS1, PIK3CA, ZNRF1, C17ORF49, GUK1, ZNF326, GCNT2, SAE1, GTPBP8, PKN1, TNFRSF1A, ELOVL1, TUT1, NCSTN, MFAP2, YBX1, PPP1R3B, PLCE1, NDST1, KCNJ8, ATF6B, GAMT, DGCR2, TPI1, JMJD8, NES, CDC42EP3, SECISBP2L, PRPF8, DNAJC4, EIF3B, EPHX1, WDTC1, MAML1, CRNKL1, MAPK3, RPL18A, FAM83B, SERTAD2, FOXK1, ZNF646, CAMSAP3, ACVR2B, ATXN7L1, LRRC8B, PLOD3, FAM193B, ZFHX3, SMURF1, AOX1, RPS15, PRMT6, TRIM24, PRKCSH, ETV6, GSK3A, WDR74, KCNC4, LARP6, BCOR, PSD4, RAB4A, ZNF341, SGTA, STUB1, SLC35E1, NPAS2, MAOA, USP1, RBM47, RNF43, KIAA0930, SHISA5, TCOF1, NCL, FKBP5, RBM22, SLC48A1, TRAPPC3, METTL14, MZT2B, ATF7IP, RWDD2B, POLR1D, UBIAD1, SFXN2, FIGN, UBAP2, UBE2E2, MX1, SPPL3, KCTD11, HSP90AA1, BTG1, GAB1, PXMP2, S1PR1, THRB, UTP14A, EDNRB, CRTC3, SYBU, MED25, ERP29, RBM23, CDNF, RNASEK, CASC3, MTMR3, TNKS, SF3A1, CHID1, AGPAT3, MEX3C, SIAH2, ACD, NFKBIA, CDC42EP4, ARHGAP17, GJA1, RNPS1, ATP13A2, ZSWIM3, TXNDC5, PPFIBP2, MRPL49, SLC35C2, CD34, SQSTM1, DEAF1, CPNE8, INSR, RPL19, NR1D2, AGBL5, SP2, YIPF3, MRPS18B, NEDD9, GADD45G, ZNRF3, MAST3, SNX12, TMED5, BAZ2A, CCDC86, MBOAT7, GID8, DCPS, LIG3, BSG, SP4, KLF11, CIZ1, LNX1, RALB, BTF3, NET1
GO:0140110	MF	Transcription regulator activity	0.019982707	1.699345683	71	TBX22, OTX2, HSFX2, ZSCAN10, TFAP2C, RUNX1T1, ZNF554, EGR1, ETV4, BCL9, POU6F2, BTG2, IRF2BP2, KLF15, EGR3, IKZF4, CREB3L1, EMX1, KLF3, ETV5, TLE3, HEYL, GLI3, MYC, ZNF416, DBP, KLF9, KLF10, MNT, FOSL2, ZNF362, NR1D1, FOXN3, ZNF581, KLF6, PBX1, STAT1, NR0B2, TCF7L2, HELZ2, SMAD1, TP53, ELK1, BACH2, ETS1, TSHZ1, MED13L, PKN1, ATF6B, MAML1, NFIB, SERTAD2, FOXK1, ZNF646, ZFHX3, TRIM24, ETV6, BCOR, ZNF341, NPAS2, ATF7IP, BTG1, THRB, SIAH2, DEAF1, NR1D2, SP2, SLC2A4RG, SP4, KLF11, ZNF836
**Down regulated genes**						
GO:0051606	BP	Detection of stimulus	9.55E−21	20.01997014	63	DMBT1, OR10J3, OR4F5, OR6C74, OR5L1, OR4D2, OR2T33, OR2T6, GJA10, OR8H2, OR4N2, CASQ2, OR2A25, TAS2R7, OR1E2, OR2B3, OR6N1, OR4K5, OR4A5, OR5AN1, OR5AS1, OR13G1, OR2M5, OR13C5, OR4C3, OR52L1, OR8K1, OR6B2, OR56A4, OR5B17, OR51A2, OR5T1, OR1A1, OR13C8, OR13C2, OR51B4, OR2AG2, OR2M7, OR51L1, OR5H1, OR8G1, OR6K6, OR10Z1, OR4K1, OR5T2, OR2A12, OR1L8, OR1J2, TAS2R60, CHRNA10, OR5AP2, OR52K1, OR9A2, OR10A5, OR4C46, OR52W1, TAS2R8, OR52E6, TTN, OR6K3, OR11H2, OR52D1, PKDREJ
GO:0032501	BP	Multicellular organismal process	3.26E−07	6.486491268	224	IAPP, HAPLN4, ADCYAP1, DMBT1, RGS16, PREX1, NRG3, LRRTM3, GABRA2, GTSF1, NTNG2, GLRA1, DGKG, ISX, OR10J3, SLC6A17, DACH2, IFNA16, OR4F5, SLC18A2, KLHL1, OR6C74, DKK4, UNC5A, OR5L1, OR4D2, OR2T33, OR2T6, CDHR1, GJA10, BMP5, OR8H2, INSRR, POSTN, OR4N2, CASQ2, OR2A25, CYP24A1, TAS2R7, OR1E2, OR2B3, SLC45A3, IFNA10, LRRC10, GREM2, OR6N1, CAPN8, TFF3, OR4K5, HOPX, COMP, OR4A5, OR5AN1, KRTAP1-3, OR5AS1, GABRR2, OR13G1, OR2M5, HTR3A, KRTAP13-3, OR13C5, OR4C3, CHST8, MAS1, OR52L1, KRTAP4-2, OR8K1, KIRREL3, OR6B2, KRTAP19-1, KRTAP3-1, KRTAP3-2, KRTAP9-9, OR56A4, PSMA6, OR5B17, TMEM108, OR51A2, P2RX5, OR5T1, OR1A1, OR13C8, OR13C2, GAP43, MFAP5, ADAMTSL1, OR51B4, LCE1F, KCNQ2, SFRP4, OR2AG2, OR2M7, OR51L1, OR5H1, CRLF1, KRTAP21-2, OR8G1, OR6K6, KRTAP19-2, OR10Z1, KRTAP10-1, DNAH11, NPTX2, OR4K1, OR5T2, EDARADD, PI16, JPH2, INSC, FN1, ACTBL2, C1QL1, NPFF, OR2A12, BEND6, OR1L8, HBD, OR1J2, TAS2R60, VSIG4, IGF2BP3, CHRNA10, OR5AP2, OR52K1, CYP26B1, FAT3, IL10, TNFRSF11A, GCGR, OR9A2, LCN2, TOP2A, MESP2, CDH22, OR10A5, OR4C46, UCHL1, FGL1, ACTC1, VASP, CIT, CTNNA3, OR52W1, IL5RA, SOSTDC1, TTC8, RADIL, TAS2R8, NTN1, OR52E6, P2RX6, CTNNA2, TTN, OR6K3, MUSTN1, OR11H2, CCR2, P2RY12, CNN1, HRC, PTGS2, DEF8, APOC1, LYZ, GLP1R, NDRG4, CNGA4, APOC2, KRTAP5-1, HADH, SERPINA3, OR52D1, TMEM178A, EFNA4, PIR, HSD17B3, PTGFR, SCRG1, NR6A1, LGI2, AKR1C2, CDH23, ADAMTS2, P2RY1, IGSF11, TSHZ3, PDLIM3, CRYGS, KIF20B, ESR1, ARHGAP22, AGAP2, CYP4F12, SLC26A7, TFCP2L1, RELT, MAP1A, LOXL1, KIF18A, PRICKLE4, JMJD6, ARG2, POU5F1, ROBO1, ALOX5, ANLN, CDK1, SELPLG, GREB1, MYH11, ABCB1, CYP2J2, VAV3, TYMS, SCN1B, MATN3, LAMB3, SRD5A1, SRPX2, SGIP1, GLG1, TPM2, SIX2, SAMHD1
GO:0071944	CC	Cell periphery	1.40E−07	6.853891822	186	HAPLN4, CSNK1G1, IGLL5, PLCH2, SLCO1A2, DMBT1, RGS16, PREX1, LRFN2, NRG3, LRRTM3, GABRA2, NTNG2, GLRA1, DGKG, OR10J3, SLC6A17, OR4F5, SLC18A2, SLC26A9, OR6C74, UNC5A, OR5L1, OR4D2, KCNG3, SLC27A6, OR2T33, CLDN25, OR2T6, ADAM30, CDHR1, GJA10, RASD1, TAAR9, OR8H2, INSRR, POSTN, OR4N2, OR2A25, LY6G6E, TAS2R7, KIF20A, OR1E2, OR2B3, SLC45A3, OR6N1, OR4K5, COMP, OR4A5, OR5AN1, OR5AS1, GABRR2, OR13G1, OR2M5, HTR3A, OR13C5, OR4C3, MAS1, OR52L1, OR8K1, KIRREL3, OR6B2, SLCO5A1, OR56A4, OR5B17, PCDH7, OR51A2, P2RX5, SLC22A9, OR5T1, OR1A1, OR13C8, OR13C2, GAP43, MFAP5, ADAMTSL1, OR51B4, KCNQ2, KCNF1, OR2AG2, CD1E, OR2M7, OR51L1, OR5H1, CRLF1, OR8G1, OR6K6, OR10Z1, KCNH1, OR4K1, OR5T2, SDR16C5, JPH2, INSC, FN1, OR2A12, CAPNS2, YIPF4, OR1L8, OR1J2, TAS2R60, CHRNA10, OR5AP2, MEP1B, OR52K1, FAT3, TNFRSF11A, GCGR, OR9A2, ENTPD3, OXGR1, CDH22, OR10A5, OR4C46, UCHL1, HLA-C, CLEC4D, FGL1, VASP, OR52W1, IL5RA, TTC8, TAS2R8, OLFM4, TSPAN1, GPR141, NTN1, OR52E6, P2RX6, CTNNA2, TTN, OR6K3, OR11H2, CCR2, PRAM1, P2RY12, PTGS2, ITGBL1, GLP1R, NDRG4, KLRK1, CNGA4, SERPINA3, OR52D1, EFNA4, PTGFR, CFB, FCHO1, LRRC7, MMP28, CDH23, ADAMTS2, ATP2C1, P2RY1, IGSF11, TSHZ3, FRMPD1, SELL, ESR1, CYP4F12, RASD2, SLC26A7, LY6G5B, CSF2RA, RELT, LOXL1, KIF18A, B3GNT3, MYO1F, JMJD6, ROBO1, ANLN, SELPLG, ABCB1, DLG2, ADAMTS10, VAV3, SCN1B, LAT, MATN3, LAMB3, SRPX2, SGIP1, SLC7A7, GLG1, SAMHD1
GO:0005886	CC	Plasma membrane	9.39E−07	6.027126436	171	CSNK1G1, IGLL5, PLCH2, SLCO1A2, RGS16, PREX1, LRFN2, NRG3, LRRTM3, GABRA2, NTNG2, GLRA1, DGKG, OR10J3, SLC6A17, OR4F5, SLC18A2, SLC26A9, OR6C74, UNC5A, OR5L1, OR4D2, KCNG3, SLC27A6, OR2T33, CLDN25, OR2T6, ADAM30, CDHR1, GJA10, RASD1, TAAR9, OR8H2, INSRR, OR4N2, OR2A25, LY6G6E, TAS2R7, KIF20A, OR1E2, OR2B3, SLC45A3, OR6N1, OR4K5, OR4A5, OR5AN1, OR5AS1, GABRR2, OR13G1, OR2M5, HTR3A, OR13C5, OR4C3, MAS1, OR52L1, OR8K1, KIRREL3, OR6B2, SLCO5A1, OR56A4, OR5B17, PCDH7, OR51A2, P2RX5, SLC22A9, OR5T1, OR1A1, OR13C8, OR13C2, GAP43, ADAMTSL1, OR51B4, KCNQ2, KCNF1, OR2AG2, CD1E, OR2M7, OR51L1, OR5H1, CRLF1, OR8G1, OR6K6, OR10Z1, KCNH1, OR4K1, OR5T2, SDR16C5, JPH2, INSC, FN1, OR2A12, CAPNS2, YIPF4, OR1L8, OR1J2, TAS2R60, CHRNA10, OR5AP2, MEP1B, OR52K1, FAT3, TNFRSF11A, GCGR, OR9A2, ENTPD3, OXGR1, CDH22, OR10A5, OR4C46, UCHL1, HLA-C, CLEC4D, VASP, OR52W1, IL5RA, TTC8, TAS2R8, OLFM4, TSPAN1, GPR141, OR52E6, P2RX6, CTNNA2, TTN, OR6K3, OR11H2, CCR2, PRAM1, P2RY12, PTGS2, ITGBL1, GLP1R, NDRG4, KLRK1, CNGA4, OR52D1, EFNA4, PTGFR, CFB, FCHO1, LRRC7, CDH23, ATP2C1, P2RY1, IGSF11, TSHZ3, FRMPD1, SELL, ESR1, CYP4F12, RASD2, SLC26A7, LY6G5B, CSF2RA, RELT, KIF18A, B3GNT3, MYO1F, JMJD6, ROBO1, SELPLG, ABCB1, DLG2, VAV3, SCN1B, LAT, SRPX2, SGIP1, SLC7A7, GLG1, SAMHD1
GO:0004888	MF	Transmembranesignaling receptor activity	4.64E−19	18.3338049	83	GABRA2, GLRA1, OR10J3, OR4F5, OR6C74, UNC5A, OR5L1, OR4D2, OR2T33, OR2T6, TAAR9, OR8H2, INSRR, OR4N2, OR2A25, TAS2R7, OR1E2, OR2B3, OR6N1, OR4K5, OR4A5, OR5AN1, OR5AS1, GABRR2, OR13G1, OR2M5, HTR3A, OR13C5, OR4C3, MAS1, OR52L1, OR8K1, OR6B2, OR56A4, OR5B17, OR51A2, P2RX5, OR5T1, OR1A1, OR13C8, OR13C2, OR51B4, OR2AG2, OR2M7, OR51L1, OR5H1, CRLF1, OR8G1, OR6K6, OR10Z1, OR4K1, OR5T2, OR2A12, OR1L8, OR1J2, TAS2R60, CHRNA10, OR5AP2, OR52K1, TNFRSF11A, GCGR, OR9A2, OXGR1, OR10A5, OR4C46, OR52W1, IL5RA, SOSTDC1, TAS2R8, GPR141, OR52E6, P2RX6, OR6K3, OR11H2, CCR2, P2RY12, GLP1R, OR52D1, EFNA4, PTGFR, P2RY1, CSF2RA, ROBO1
GO:0060089	MF	Molecular transducer activity	2.72E-18	17.56616903	88	DMBT1, GABRA2, GLRA1, OR10J3, RXRG, OR4F5, OR6C74, UNC5A, OR5L1, OR4D2, OR2T33, OR2T6, TAAR9, OR8H2, INSRR, OR4N2, OR2A25, TAS2R7, OR1E2, OR2B3, OR6N1, OR4K5, OR4A5, OR5AN1, OR5AS1, GABRR2, OR13G1, OR2M5, HTR3A, OR13C5, OR4C3, MAS1, OR52L1, OR8K1, OR6B2, OR56A4, OR5B17, OR51A2, P2RX5, OR5T1, OR1A1, OR13C8, OR13C2, OR51B4, OR2AG2, OR2M7, OR51L1, OR5H1, CRLF1, OR8G1, OR6K6, OR10Z1, OR4K1, OR5T2, OR2A12, OR1L8, OR1J2, TAS2R60, CHRNA10, OR5AP2, OR52K1, TNFRSF11A, GCGR, OR9A2, OXGR1, OR10A5, OR4C46, OR52W1, IL5RA, SOSTDC1, TAS2R8, GPR141, OR52E6, P2RX6, OR6K3, OR11H2, CCR2, P2RY12, GLP1R, KLRK1, OR52D1, EFNA4, PTGFR, P2RY1, ESR1, CSF2RA, JMJD6, ROBO1

**Table 2:** The enriched pathway terms of the up and down regulated differentially expressed genes.

Pathway ID	Pathway name	Adjusted p value	Negative log10 of adjusted p value	Gene count	Gene
**Up regulated genes**					
REAC:R-HSA-5663202	Diseases of signal transduction by growth factor receptors and second messengers	0.002918856	2.534787284	25	FGA, FGB, FGG, HEYL, HRAS, MYC, IRS1, KIAA1549, STAT1, SYVN1, TCF7L2, DLL4, SOS1, EGFR, PIK3CA, NCSTN, MAML1, MAPK3, TRIM24, ETV6, GSK3A, RNF43, HSP90AA1, GAB1, TNKS
REAC:R-HSA-6809371	Formation of the cornified envelope	0.009712336	2.012676308	12	KRT6A, LCE3D, SPRR3, FLG, SPRR2D, SPRR1B, SPRR1A, KRT16, KRT13, SPRR2E, LCE3E, KLK13
REAC:R-HSA-156827	L13a-mediated translational silencing of Ceruloplasmin expression	0.027095018	1.567110554	10	RPS6, RPL8, RPS28, EIF4B, RPL18, EIF3B, RPL18A, RPS15, RPL39, RPL19
REAC:R-HSA-1643685	Disease	0.027095018	1.567110554	60	CGA, FGA, FGB, FGG, CALCR, ABCB11, MUC21, GCG, ADAMTS8, PTGER4, GPC6, ABCC9, RPS6, SLC29A3, RPL8, HEYL, HRAS, SFTPD, MYC, IRS1, RPS28, MUC6, NUP205, WASF1, PAPSS2, KIAA1549, IL6R, ABCA1, RPL18, STAT1, FMOD, ARPC1A, SYVN1, TCF7L2, DLL4, ELK1, SOS1, PRKACA, EGFR, PIK3CA, NCSTN, MAML1, MAPK3, RPL18A, RPS15, TRIM24, PRKCSH, ETV6, GSK3A, MAOA, RNF43, HSP90AA1, GAB1, S1PR1, RPL39, TNKS, NFKBIA, RPL19, SLC39A4, BSG
REAC:R-HSA-9006934	Signaling by receptor tyrosine kinases	0.03209698	1.493535827	25	IGF2, EGR1, COL6A3, VEGFC, ID3, EGR3, HRAS, IRS1, WASF1, DUSP4, STAT1, ELK1, PTBP1, SOS1, PRKACA, EGFR, PIK3CA, NCSTN, MAPK3, RAB4A, STUB1, HSP90AA1, GAB1, INSR, RALB
REAC:R-HSA-1266738	Developmental biology	0.032172975	1.492508778	45	KRT6A, LCE3D, SPRR3, FLG, SPRR2D, SPRR1B, SPRR1A, DCC, KRT16, ZSCAN10, KRT13, SPRR2E, LCE3E, COL6A3, MPZ, KLK13, RPS6, RPL8, HRAS, MYC, RPS28, IL6R, PBX1, RPL18, ARPC1A, HELZ2, SOS1, PRKACA, EGFR, PIK3CA, MED13L, NCSTN, MAML1, MAPK3, RPL18A, ACVR2B, RPS15, HSP90AA1, GAB1, RPL39, MED25, CASC3, SIAH2, RNPS1, RPL19
**Down regulated genes**					
REAC:R-HSA-381753	Olfactory signaling pathway	4.32E−23	22.36427935	54	OR10J3, OR4F5, OR6C74, OR5L1, OR4D2, OR2T33, OR2T6, OR8H2, OR4N2, OR2A25, OR1E2, OR2B3, OR6N1, OR4K5, OR4A5, OR5AN1, OR5AS1, OR13G1, OR2M5, OR13C5, OR4C3, OR52L1, OR8K1, OR6B2, OR56A4, OR5B17, OR51A2, OR5T1, OR1A1, OR13C8, OR13C2, OR51B4, OR2AG2, OR2M7, OR51L1, OR5H1, OR8G1, OR6K6, OR10Z1, OR4K1, OR5T2, OR2A12, OR1L8, OR1J2, OR5AP2, OR52K1, OR9A2, OR10A5, OR4C46, OR52W1, OR52E6, OR6K3, OR11H2, OR52D1
REAC:R-HSA-9709957	Sensory perception	4.85E−21	20.31389969	57	OR10J3, OR4F5, OR6C74, OR5L1, OR4D2, OR2T33, OR2T6, OR8H2, OR4N2, OR2A25, OR1E2, OR2B3, OR6N1, OR4K5, OR4A5, OR5AN1, OR5AS1, OR13G1, OR2M5, OR13C5, OR4C3, OR52L1, OR8K1, OR6B2, OR56A4, OR5B17, OR51A2, OR5T1, OR1A1, OR13C8, OR13C2, OR51B4, OR2AG2, OR2M7, OR51L1, OR5H1, OR8G1, OR6K6, OR10Z1, OR4K1, OR5T2, OR2A12, OR1L8, RDH16, OR1J2, OR5AP2, OR52K1, OR9A2, OR10A5, OR4C46, OR52W1, OR52E6, OR6K3, BCO2, OR11H2, APOC2, OR52D1
REAC:R-HSA-418555	G alpha (s) signalling events	6.29E−20	19.20156126	58	IAPP, ADCYAP1, OR10J3, OR4F5, OR6C74, OR5L1, OR4D2, OR2T33, OR2T6, TAAR9, OR8H2, OR4N2, OR2A25, OR1E2, OR2B3, OR6N1, OR4K5, OR4A5, OR5AN1, OR5AS1, OR13G1, OR2M5, OR13C5, OR4C3, OR52L1, OR8K1, OR6B2, OR56A4, OR5B17, OR51A2, OR5T1, OR1A1, OR13C8, OR13C2, OR51B4, OR2AG2, OR2M7, OR51L1, OR5H1, OR8G1, OR6K6, OR10Z1, OR4K1, OR5T2, OR2A12, OR1L8, OR1J2, OR5AP2, OR52K1, OR9A2, OR10A5, OR4C46, OR52W1, OR52E6, OR6K3, OR11H2, GLP1R, OR52D1
REAC:R-HSA-388396	GPCR downstream signalling	9.32E−15	14.0308131	76	IAPP, ADCYAP1, RGS16, PREX1, DGKG, OR10J3, OR4F5, OR6C74, OR5L1, OR4D2, ARHGEF35, OR2T33, OR2T6, TAAR9, OR8H2, OR4N2, OR2A25, TAS2R7, OR1E2, OR2B3, OR6N1, OR4K5, OR4A5, OR5AN1, OR5AS1, OR13G1, OR2M5, OR13C5, OR4C3, OR52L1, OR8K1, OR6B2, OR56A4, OR5B17, OR51A2, OR5T1, OR1A1, OR13C8, OR13C2, OR51B4, OR2AG2, OR2M7, OR51L1, OR5H1, OR8G1, OR6K6, OR10Z1, OR4K1, OR5T2, NPFF, OR2A12, OR1L8, RDH16, OR1J2, TAS2R60, OR5AP2, OR52K1, OR9A2, OXGR1, OR10A5, OR4C46, OR52W1, TAS2R8, OR52E6, OR6K3, BCO2, OR11H2, CCR2, P2RY12, GLP1R, APOC2, OR52D1, PTGFR, P2RY1, PDE1C, VAV3
REAC:R-HSA-372790	Signaling by GPCR	2.45E−13	12.61114695	76	IAPP, ADCYAP1, RGS16, PREX1, DGKG, OR10J3, OR4F5, OR6C74, OR5L1, OR4D2, ARHGEF35, OR2T33, OR2T6, TAAR9, OR8H2, OR4N2, OR2A25, TAS2R7, OR1E2, OR2B3, OR6N1, OR4K5, OR4A5, OR5AN1, OR5AS1, OR13G1, OR2M5, OR13C5, OR4C3, OR52L1, OR8K1, OR6B2, OR56A4, OR5B17, OR51A2, OR5T1, OR1A1, OR13C8, OR13C2, OR51B4, OR2AG2, OR2M7, OR51L1, OR5H1, OR8G1, OR6K6, OR10Z1, OR4K1, OR5T2, NPFF, OR2A12, OR1L8, RDH16, OR1J2, TAS2R60, OR5AP2, OR52K1, OR9A2, OXGR1, OR10A5, OR4C46, OR52W1, TAS2R8, OR52E6, OR6K3, BCO2, OR11H2, CCR2, P2RY12, GLP1R, APOC2, OR52D1, PTGFR, P2RY1, PDE1C, VAV3
REAC:R-HSA-162582	Signal transduction	7.38146E−05	4.131857963	105	IAPP, ADCYAP1, RGS16, PREX1, NRG3, DLK1, DGKG, OR10J3, RXRG, OR4F5, OR6C74, DKK4, OR5L1, OR4D2, ARHGEF35, OR2T33, OR2T6, TAAR9, OR8H2, OR4N2, OR2A25, TAS2R7, OR1E2, OR2B3, GREM2, OR6N1, TFF3, OR4K5, OR4A5, OR5AN1, OR5AS1, OR13G1, OR2M5, OR13C5, OR4C3, OR52L1, OR8K1, OR6B2, OR56A4, PSMA6, DLGAP5, OR5B17, OR51A2, OR5T1, OR1A1, OR13C8, OR13C2, OR51B4, OR2AG2, OR2M7, OR51L1, OR5H1, OR8G1, OR6K6, OR10Z1, OR4K1, OR5T2, SDR16C5, FN1, NPFF, OR2A12, OR1L8, RDH16, OR1J2, TAS2R60, OR5AP2, OR52K1, CYP26B1, OR9A2, OXGR1, OR10A5, SPC24, OR4C46, CIT, OR52W1, IL5RA, TAS2R8, OR52E6, OR6K3, BCO2, OR11H2, CCR2, P2RY12, APOC1, GLP1R, APOC2, OR52D1, SMAD9, PTGFR, BUB1B, P2RY1, ARHGAP20, NEDD8, ESR1, ARHGAP22, CSF2RA, KIF18A, ARHGAP9, CDK1, GREB1, MYH11, PDE1C, DLG2, VAV3, LAMB3

### Construction of the PPI network and module analysis

**Figure 3:**
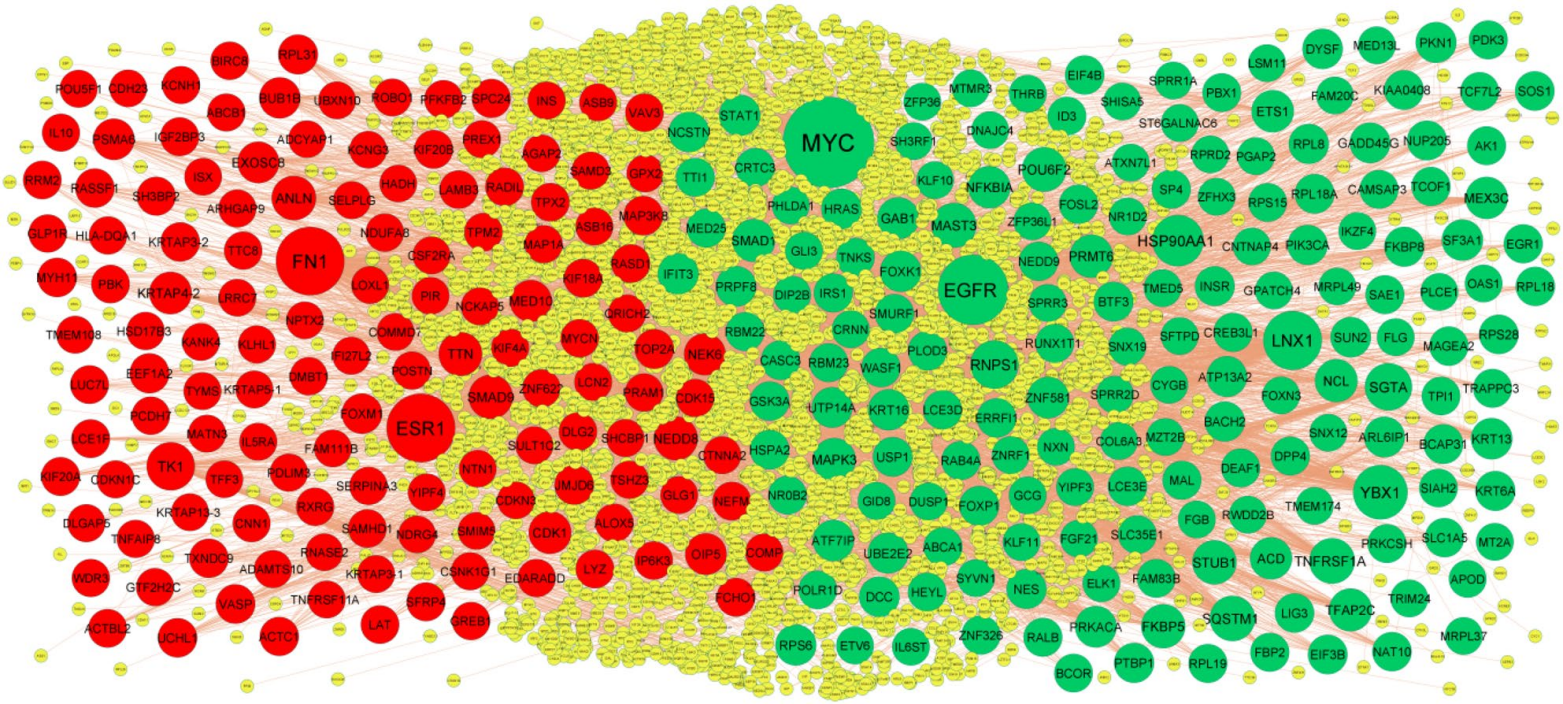
PPI network of DEGs. The PPI network of DEGs was constructed using Cytoscap. Up regulated genes are marked in green; down regulated genes are marked in red.

**Figure 4:**
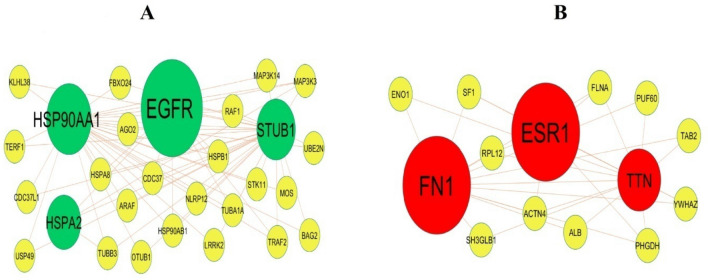
Modules of isolated form PPI of DEGs. (**A**) The most significant module was obtained from PPI network with 28 nodes and 63 edges for up regulated genes (**B**) The most significant module was obtained from PPI network with 14 nodes and 30 edges for down regulated genes. Up regulated genes are marked in green; down regulated genes are marked in red.

The PPI network of the DEGs was constructed with 5111 nodes and 9392 edges by using the IntAct database (Fig. 3). A node with a higher node degree, betweenness centrality, stress centrality and closeness centrality consider as a hub genes and are listed in Table 3. The hub genes included MYC, EGFR, LNX1, YBX1, HSP90AA1, ESR1, FN1, TK1, ANLN and SMAD9. To detect significant modules in the PPI network, the PEWCC1 plug‐in was used for analysis, and two modules that had the highest degree stood out. GO and pathway enrichment analysis showed that module 1 contained 28 nodes and 63 edges (Fig. 4A), which were associated with diseases of signal transduction by growth factor receptors and second messengers, disease, nitrogen compound metabolic process and membrane-enclosed lumen, while module 2 had 14 nodes and 30 edges (Fig. 4B), which were mainly associated with signal transduction, multicellular organismal process and detection of stimulus.

**Table 3:** Topology table for up and down regulated genes.

Regulation	Node	Degree	Betweenness	Stress	Closeness
Up	MYC	769	0.24506	1.04E + 08	0.402932
Up	EGFR	469	0.138032	61,812,344	0.381322
Up	LNX1	301	0.098028	35,639,540	0.344753
Up	YBX1	242	0.052423	15,440,248	0.370488
Up	HSP90AA1	198	0.042012	18,645,766	0.349323
Up	RNPS1	148	0.029398	16,186,898	0.324119
Up	SGTA	139	0.039126	11,319,544	0.315439
Up	TNFRSF1A	116	0.016842	8,191,032	0.328598
Up	SQSTM1	112	0.025913	6,213,766	0.344335
Up	MAST3	105	0.022	12,287,012	0.305669
Up	POU6F2	99	0.028224	4,939,174	0.317338
Up	STUB1	95	0.01869	5,557,470	0.333856
Up	FKBP5	94	0.015263	3,991,284	0.329764
Up	PRMT6	82	0.023047	4,016,020	0.301486
Up	MAPK3	78	0.01798	4,710,364	0.312259
Up	SMAD1	77	0.014129	5,216,492	0.309237
Up	TFAP2C	77	0.020062	3,309,460	0.333573
Up	NCL	73	0.015947	5,344,646	0.374917
Up	ETS1	69	0.014065	3,895,426	0.310987
Up	MEX3C	68	0.003769	2,594,352	0.310439
Up	ACD	66	0.014094	3,525,238	0.307138
Up	NFKBIA	66	0.008138	4,939,524	0.310854
Up	GAB1	61	0.005759	2,357,788	0.296365
Up	ZNF581	58	0.008074	2,392,056	0.303509
Up	PRKACA	58	0.012141	2,683,394	0.312833
Up	KRT13	56	0.009539	2,356,276	0.31577
Up	SF3A1	54	0.005297	3,631,052	0.304231
Up	STAT1	54	0.010129	1,749,810	0.330531
Up	ATF7IP	54	0.011319	2,610,676	0.297746
Up	FOXK1	52	0.010322	2,431,366	0.309968
Up	KRT16	52	0.009137	2,768,286	0.302646
Up	RPS6	50	0.005709	1,809,230	0.323503
Up	IFIT3	48	0.004333	1,419,676	0.307396
Up	UBE2E2	48	0.009384	2,778,786	0.289714
Up	PKN1	47	0.007325	2,119,206	0.29714
Up	ARL6IP1	46	0.011853	5,167,578	0.284047
Up	UTP14A	45	0.00788	1,716,012	0.322239
Up	PRPF8	44	0.006824	1,731,078	0.329977
Up	GADD45G	44	0.007648	1,727,574	0.29879
Up	ATP13A2	43	0.011364	3,235,048	0.28605
Up	HRAS	42	0.006113	1,934,688	0.306916
Up	DCC	42	0.005613	2,081,812	0.292265
Up	SUN2	42	0.004561	2,137,880	0.299806
Up	DYSF	41	0.007116	2,286,238	0.293962
Up	NCSTN	40	0.007729	5,776,106	0.274308
Up	RPL8	37	0.004561	1,421,422	0.324922
Up	BACH2	37	0.008838	2,796,552	0.273369
Up	PIK3CA	37	0.005768	1,280,756	0.304576
Up	PTBP1	36	0.003339	1,190,818	0.333507
Up	FKBP8	35	0.00586	1,371,274	0.3223
Up	NES	35	0.007013	1,126,640	0.295338
Up	KRT6A	34	0.005784	1,381,296	0.320281
Up	NUP205	33	0.005495	1,090,580	0.323442
Up	FOXP1	33	0.006278	1,144,548	0.31992
Up	INSR	33	0.003132	1,562,024	0.279757
Up	BCAP31	32	0.00482	1,838,970	0.292131
Up	BTF3	31	0.00454	1,476,600	0.290636
Up	RPL18A	31	0.002498	754,566	0.318306
Up	KIAA0408	30	0.00425	1,221,042	0.302575
Up	USP1	30	0.004591	2,441,058	0.280879
Up	EIF3B	29	0.003846	916,674	0.315848
Up	HSPA2	28	0.002862	684,442	0.313947
Up	RPS28	28	0.002873	1,278,818	0.301718
Up	SOS1	28	0.002513	879,664	0.284205
Up	ERRFI1	26	0.002199	611,152	0.311214
Up	LCE3E	26	0.001327	524,110	0.25448
Up	GSK3A	26	0.004252	1,655,588	0.292867
Up	NAT10	26	0.001696	666,842	0.325191
Up	LIG3	26	0.00228	725,644	0.294691
Up	RPL18	25	9.73E−04	477,540	0.322646
Up	SLC1A5	25	0.004674	807,018	0.306677
Up	EIF4B	25	0.00188	620,280	0.295594
Up	NEDD9	24	0.003127	549,922	0.289271
Up	CASC3	23	0.002491	601,190	0.302503
Up	FOSL2	23	0.003682	831,006	0.310911
Up	SMURF1	22	0.002704	664,080	0.284142
Up	PLOD3	22	0.003554	1,082,282	0.335808
Up	TCOF1	22	0.00218	501,286	0.314295
Up	ZNF326	22	0.001349	640,724	0.29001
Up	SP4	22	0.003993	853,624	0.26749
Up	EGR1	21	0.002422	466,912	0.302056
Up	MAGEA2	21	0.004402	1,011,862	0.293759
Up	CREB3L1	21	0.006704	4,786,040	0.227129
Up	WASF1	20	0.003044	1,067,564	0.274868
Up	IRS1	20	0.001568	411,892	0.309837
Up	CRTC3	20	0.002135	967,284	0.279558
Up	FBP2	20	9.48E−04	423,694	0.288846
Up	MED25	20	0.001218	294,794	0.275609
Up	LCE3D	20	4.82E−04	304,904	0.263329
Up	DEAF1	19	0.002531	618,844	0.3017
Up	PDK3	19	0.003011	604,606	0.307526
Up	RPL19	19	6.37E−04	392,064	0.319241
Up	ABCA1	19	0.004473	1,062,228	0.298441
Up	SAE1	19	0.003167	702,666	0.299157
Up	MAL	19	0.004533	1,470,896	0.264227
Up	RAB4A	19	0.003857	747,204	0.292398
Up	RALB	19	0.001738	1,634,506	0.255536
Up	TRAPPC3	19	0.005096	1,890,182	0.252046
Up	DPP4	19	0.00527	1,619,646	0.234023
Up	PRKCSH	18	0.002123	375,070	0.316043
Up	RBM22	18	0.002051	950,490	0.283386
Up	CAMSAP3	18	0.002085	1,031,300	0.274014
Up	AK1	18	0.002632	1,174,620	0.257986
Up	TTI1	17	0.001947	994,560	0.275505
Up	RUNX1T1	17	0.002454	1,455,104	0.275046
Up	RPS15	16	0.00225	468,552	0.316395
Up	NR0B2	16	0.002709	597,146	0.313581
Up	POLR1D	16	0.001752	656,232	0.282369
Up	FAM83B	16	8.49E−04	301,500	0.277314
Up	PBX1	16	0.001944	703,560	0.258718
Up	TCF7L2	15	0.001345	373,312	0.28326
Up	BCOR	15	0.002336	806,128	0.273164
Up	ID3	15	0.002818	819,716	0.269962
Up	SLC35E1	15	0.003323	1,409,720	0.24654
Up	TPI1	14	0.002071	457,522	0.314391
Up	SIAH2	14	0.001994	845,214	0.269677
Up	TNKS	14	0.002475	650,184	0.251959
Up	RBM23	14	2.87E−04	166,572	0.276758
Up	HEYL	14	0.002211	402,860	0.280864
Up	MRPL37	2	1.61E−04	51,382	0.28108
Up	GID8	2	4.01E−05	6218	0.246921
Up	DIP2B	2	0	0	0.291299
Up	SH3RF1	2	2.53E−04	99,400	0.287919
Up	ZFP36L1	2	9.45E−05	13,940	0.255792
Up	GCG	2	1.27E−04	41,870	0.209131
Up	YIPF3	2	7.28E−05	18,416	0.211048
Up	FGB	1	0	0	0.254404
Up	FAM20C	1	0	0	0.254404
Up	GLI3	1	0	0	0.236207
Up	NXN	1	0	0	0.215614
Up	PHLDA1	1	0	0	0.276071
Up	SPRR1A	1	0	0	0.276071
Up	DNAJC4	1	0	0	0.276071
Up	MRPL49	1	0	0	0.270347
Up	COL6A3	1	0	0	0.22719
Up	MZT2B	1	0	0	0.287223
Up	ZFHX3	1	0	0	0.287223
Up	SNX19	1	0	0	0.287223
Up	KLF10	1	0	0	0.287223
Up	ST6GALNAC6	1	0	0	0.287223
Up	RPRD2	1	0	0	0.287223
Up	ETV6	1	0	0	0.287223
Up	SYVN1	1	0	0	0.273764
Up	SPRR2D	1	0	0	0.229084
Up	FGF21	1	0	0	0.239809
Up	TMEM174	1	0	0	0.239809
Up	CYGB	1	0	0	0.222435
Up	CRNN	1	0	0	0.256151
Up	PLCE1	1	0	0	0.258901
Up	SFTPD	1	0	0	0.201125
Up	TRIM24	1	0	0	0.273179
Up	APOD	1	0	0	0.273179
Up	THRB	1	0	0	0.21607
Up	IKZF4	1	0	0	0.23715
Up	SPRR3	1	0	0	0.23715
Up	CNTNAP4	1	0	0	0.23412
Up	ZNRF1	1	0	0	0.224644
Up	ATXN7L1	1	0	0	0.211048
Up	FLG	1	0	0	0.219296
Up	MT2A	1	0	0	0.250147
Up	SHISA5	1	0	0	0.250147
Up	RWDD2B	1	0	0	0.230063
Up	OAS1	1	0	0	0.231658
Up	TMED5	1	0	0	0.231658
Up	NR1D2	1	0	0	0.231658
Up	GPATCH4	1	0	0	0.228011
Up	KLF11	1	0	0	0.217829
Up	ZFP36	1	0	0	0.239696
Up	MTMR3	1	0	0	0.239696
Up	FOXN3	1	0	0	0.239696
Up	PGAP2	1	0	0	0.221817
Up	ELK1	1	0	0	0.237967
Up	DUSP1	1	0	0	0.237967
Up	IL6ST	1	0	0	0.248433
Up	SNX12	1	0	0	0.221222
Up	LSM11	1	0	0	0.237183
Up	MED13L	1	0	0	0.232501
Down	ESR1	448	0.115717	68,567,806	0.375827
Down	FN1	445	0.130416	47,618,446	0.393806
Down	TK1	155	0.044276	10,622,930	0.341187
Down	ANLN	102	0.019831	5,950,428	0.327945
Down	SMAD9	99	0.023512	10,495,428	0.31337
Down	NEDD8	94	0.013216	4,708,022	0.323626
Down	TTN	88	0.021791	4,877,390	0.354606
Down	CDK1	76	0.019122	4,123,802	0.343387
Down	KRTAP4-2	73	0.019925	3,654,538	0.300106
Down	MED10	65	0.012717	3,973,710	0.302915
Down	EXOSC8	61	0.015702	2,371,472	0.315244
Down	NEK6	61	0.009473	2,973,110	0.31289
Down	OIP5	60	0.013758	3,810,166	0.30688
Down	PSMA6	57	0.012284	2,937,164	0.324778
Down	BUB1B	54	0.010813	5,323,030	0.298111
Down	FOXM1	43	0.007242	2,131,654	0.300317
Down	RPL31	35	0.003717	1,156,016	0.323013
Down	EEF1A2	33	0.002074	927,396	0.307212
Down	JMJD6	31	0.006976	4,154,054	0.261751
Down	VASP	30	0.005691	1,017,020	0.316768
Down	MAP3K8	30	0.001715	773,154	0.310458
Down	UCHL1	28	0.003224	905,164	0.308808
Down	TOP2A	28	0.0032	1,042,178	0.328788
Down	MYH11	28	0.003406	1,173,838	0.305103
Down	RASSF1	27	0.004112	1,253,252	0.299175
Down	KRTAP3-2	27	0.004532	1,398,432	0.265421
Down	TXNDC9	26	0.004126	1,143,166	0.278477
Down	ACTC1	24	0.004063	1,053,202	0.329594
Down	SPC24	23	0.002762	801,496	0.268982
Down	SH3BP2	21	0.001863	813,564	0.277887
Down	IGF2BP3	20	0.001318	388,432	0.332401
Down	KIF20A	20	0.004794	850,452	0.262706
Down	SFRP4	20	0.001565	724,830	0.277404
Down	KIF20B	18	0.003465	529,620	0.306935
Down	RASD1	18	0.001969	611,526	0.282869
Down	NEFM	18	0.002405	500,776	0.29515
Down	RADIL	18	0.001925	718,708	0.28187
Down	LCE1F	18	5.31E−04	1,078,996	0.215551
Down	LUC7L	17	0.001779	331,232	0.297175
Down	LYZ	17	0.002823	462,382	0.311441
Down	PFKFB2	17	0.002624	984,686	0.285029
Down	ACTBL2	17	5.95E−04	248,094	0.310647
Down	FCHO1	17	0.002083	725,112	0.267588
Down	KIF18A	17	0.002914	1,632,588	0.264802
Down	SAMHD1	16	0.002408	964,652	0.269962
Down	LAT	15	0.002067	607,316	0.301593
Down	PBK	15	0.002668	669,380	0.294912
Down	TPX2	15	9.04E−04	407,858	0.293506
Down	RRM2	15	0.002347	419,566	0.272147
Down	LAMB3	15	0.002279	1,460,718	0.265531
Down	ALOX5	15	0.003126	496,364	0.267099
Down	TPM2	15	4.76E−04	162,954	0.290092
Down	IP6K3	14	0.001497	346,042	0.275387
Down	VAV3	14	0.001429	470,180	0.297573
Down	NDUFA8	13	0.00167	479,156	0.290455
Down	BIRC8	13	0.002152	927,372	0.24679
Down	DLG2	13	0.002105	767,688	0.273896
Down	MYCN	12	1.11E−04	41,976	0.304939
Down	PRAM1	12	0.001039	343,780	0.260883
Down	PCDH7	12	0.001704	788,890	0.266375
Down	EDARADD	11	0.001215	325,252	0.268544
Down	GREB1	11	9.15E−04	206,196	0.268968
Down	ZNF622	11	8.30E−04	730,808	0.269663
Down	RXRG	11	0.002054	800,986	0.250012
Down	GLP1R	11	3.87E−04	306,068	0.257778
Down	TTC8	11	0.003532	958,578	0.227362
Down	SERPINA3	10	0.002011	543,412	0.243151
Down	CDK15	10	2.37E−04	85,694	0.294623
Down	HLA-DQA1	10	0.001488	682,934	0.249878
Down	HSD17B3	10	1.13E−04	75,794	0.26087
Down	TSHZ3	10	0.001111	304,690	0.256253
Down	MAP1A	9	8.61E−04	207,068	0.312393
Down	CSNK1G1	9	0.001614	637,430	0.248529
Down	GTF2H2C	9	0.001734	341,202	0.245876
Down	PDLIM3	9	0.001957	559,870	0.239831
Down	WDR3	9	6.45E−04	234,640	0.274455
Down	TNFAIP8	9	9.44E−04	850,962	0.230957
Down	ADAMTS10	8	9.45E−04	260,474	0.258443
Down	ABCB1	8	9.74E−04	146,904	0.3
Down	QRICH2	8	0.001291	295,070	0.261336
Down	INS	8	4.75E−04	194,074	0.248216
Down	COMP	8	0.001207	326,670	0.231995
Down	SHCBP1	7	4.46E−04	233,846	0.256678
Down	DLGAP5	7	1.46E−04	74,806	0.290108
Down	ARHGAP9	7	3.62E−04	66,924	0.28522
Down	KCNH1	7	3.41E−04	213,892	0.253094
Down	KLHL1	6	1.15E−04	30,788	0.283638
Down	LCN2	6	4.47E−04	83,350	0.256575
Down	TYMS	6	4.58E−04	134,624	0.25317
Down	CDKN1C	6	4.20E−04	155,814	0.26589
Down	DMBT1	6	0.001176	361,022	0.251748
Down	SULT1C2	6	4.34E−04	98,520	0.244289
Down	IL10	6	8.05E−04	231,670	0.245675
Down	SAMD3	6	6.69E−04	135,970	0.268713
Down	NCKAP5	6	4.03E−05	22,814	0.2639
Down	CNN1	6	2.65E−04	64,314	0.23624
Down	PREX1	6	8.35E−04	387,030	0.254975
Down	CTNNA2	5	5.71E−04	182,504	0.281204
Down	POU5F1	5	7.40E−04	107,680	0.274839
Down	POSTN	5	5.96E−04	207,158	0.228766
Down	CSF2RA	5	8.10E−04	258,390	0.252907
Down	MATN3	5	1.69E−04	28,890	0.235848
Down	ASB9	5	5.76E−04	238,362	0.238055
Down	KRTAP5-1	5	0.001174	179,682	0.190051
Down	IL5RA	5	0.001176	281,872	0.257441
Down	KANK4	5	7.94E−04	141,232	0.240633
Down	ASB16	5	2.41E−05	16,592	0.266028
Down	KIF4A	5	4.60E−05	13,994	0.259374
Down	ROBO1	5	4.93E−04	116,624	0.246421
Down	TNFRSF11A	4	4.04E−04	116,804	0.251934
Down	KRTAP3-1	4	2.89E−05	20,452	0.252494
Down	UBXN10	4	5.63E−04	69,202	0.248047
Down	GLG1	4	4.17E−04	62,018	0.265572
Down	LOXL1	4	4.78E−04	74,588	0.250404
Down	COMMD7	4	4.03E−04	180,366	0.233127
Down	SELPLG	4	8.09E−04	244,340	0.221136
Down	YIPF4	4	4.79E−05	15,522	0.197986
Down	TMEM108	4	2.77E−05	10,972	0.243081
Down	KRTAP13-3	3	3.50E−07	422	0.210396
Down	LRRC7	3	7.82E−04	166,682	0.180159
Down	GPX2	2	0	0	0.299578
Down	CDH23	1	0	0	0.215614
Down	ISX	1	0	0	0.240905
Down	NPTX2	1	0	0	0.287223
Down	KCNG3	1	0	0	0.287223
Down	SMIM5	1	0	0	0.239809
Down	TFF3	1	0	0	0.239809
Down	FAM111B	1	0	0	0.222435
Down	CDKN3	1	0	0	0.255626
Down	IFI27L2	1	0	0	0.250147
Down	NTN1	1	0	0	0.226175
Down	AGAP2	1	0	0	0.218611
Down	PIR	1	0	0	0.238611
Down	RNASE2	1	0	0	0.215016
Down	ADCYAP1	1	0	0	0.189649
Down	HADH	1	0	0	0.248433
Down	NDRG4	1	0	0	0.221222

### MiRNA-hub gene regulatory network construction

**Figure 5:**
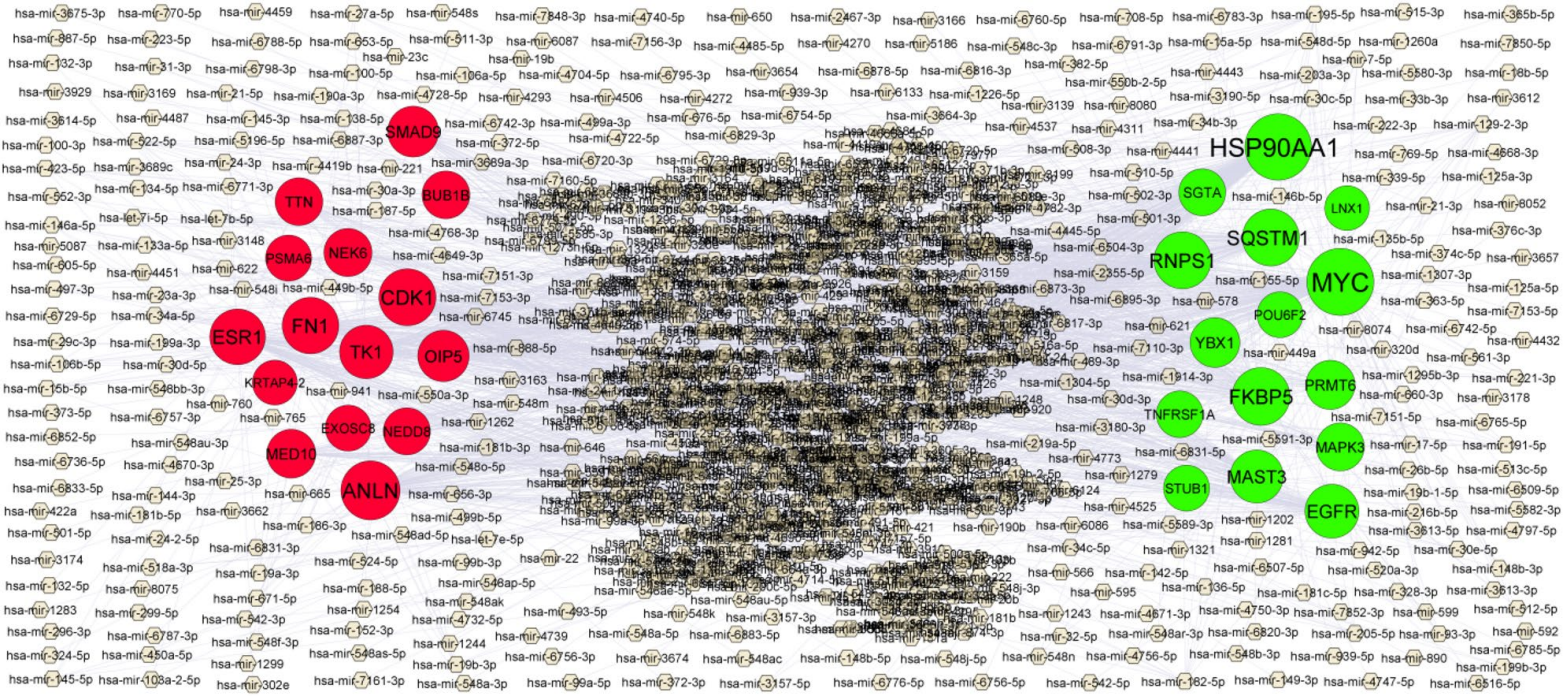
MiRNA—hub gene regulatory network. The chocolate color diamond nodes represent the key miRNAs; up regulated genes are marked in green; down regulated genes are marked in red.

The network of miRNAs and predicted targets (hub genes) is presented in Table 4. Based on the miRNAs, a miRNA -hub gene regulatory network was constructed with 2568 nodes (miRNA: 2259; hub gene: 309) and 16,618 interaction pairs (Fig. 5). Notably, MYC targeted 194 miRNAs, including hsa-mir-4677-3p; HSP90AA1 targeted 188 miRNAs, including hsa-mir-3125; FKBP5 targeted 116 miRNAs, including hsa-mir-4779; RNPS1 targeted 109 miRNAs, including hsa-mir-548az-3p; SQSTM1 targeted 108 miRNAs, including hsa-mir-106a-5p; ANLN targeted 127 miRNAs, including hsa-mir-664a-3p; CDK1 targeted 109 miRNAs, including hsa-mir-5688;FN1 targeted 105 miRNAs, including hsa-mir-199b-3p;ESR1 targeted 98 miRNAs, including hsa-mir-206; TK1 targeted 80 miRNAs, including hsa-mir-6512-3p.

**Table 4:** miRNA-target gene and TF-target gene interaction.

Regulation	Target genes	Degree	MicroRNA	Regulation	Target genes	Degree	TF
Up	MYC	194	hsa-mir-4677-3p	Up	MAPK3	48	JUND
Up	HSP90AA1	188	hsa-mir-3125	Up	HSP90AA1	35	HSF2
Up	FKBP5	116	hsa-mir-4779	Up	SQSTM1	34	SMAD4
Up	RNPS1	109	hsa-mir-548az-3p	Up	STUB1	31	ATF6
Up	SQSTM1	108	hsa-mir-106a-5p	Up	EGFR	27	ELF3
Up	EGFR	83	hsa-mir-219a-5p	Up	YBX1	24	TFAP2A
Up	MAST3	76	hsa-mir-129-2-3p	Up	LNX1	21	MAX
Up	YBX1	48	hsa-mir-1537-3p	Up	FKBP5	20	PGR
Up	PRMT6	43	hsa-mir-4330	Up	PRMT6	9	YY1
Up	MAPK3	33	hsa-mir-3158-3p	Up	SGTA	7	MXI1
Up	SGTA	24	hsa-mir-421	Up	POU6F2	7	ALX1
Up	TNFRSF1A	22	hsa-mir-548an	Up	RNPS1	7	STAT3
Up	STUB1	16	hsa-mir-942-5p	Up	TNFRSF1A	6	EP300
Up	POU6F2	15	hsa-mir-7850-5p	Up	MAST3	1	NFYA
Down	ANLN	127	hsa-mir-664a-3p	Down	ESR1	126	FOXF2
Down	CDK1	109	hsa-mir-5688	Down	SMAD9	38	XAB2
Down	FN1	105	hsa-mir-199b-3p	Down	CDK1	36	KHDRBS1
Down	ESR1	98	hsa-mir-206	Down	FN1	25	RELA
Down	TK1	80	hsa-mir-6512-3p	Down	NEK6	16	SRY
Down	OIP5	62	hsa-mir-767-5p	Down	TK1	11	DRAP1
Down	SMAD9	58	hsa-mir-3689a-3p	Down	NEDD8	10	PARP1
Down	MED10	41	hsa-mir-647	Down	TTN	10	FOXD3
Down	NEK6	34	hsa-mir-4485-3p	Down	ANLN	9	JUN
Down	BUB1B	32	hsa-mir-449b-5p	Down	BUB1B	9	MYB
Down	TTN	31	hsa-mir-181c-5p	Down	MED10	6	KDM4B
Down	NEDD8	26	hsa-mir-583	Down	PSMA6	6	EBF1
Down	PSMA6	17	hsa-mir-539-5p	Down	OIP5	5	GATA2
Down	EXOSC8	17	hsa-mir-191-5p	Down	KRTAP4-2	1	TBP

### TF-hub gene regulatory network construction

**Figure 6:**
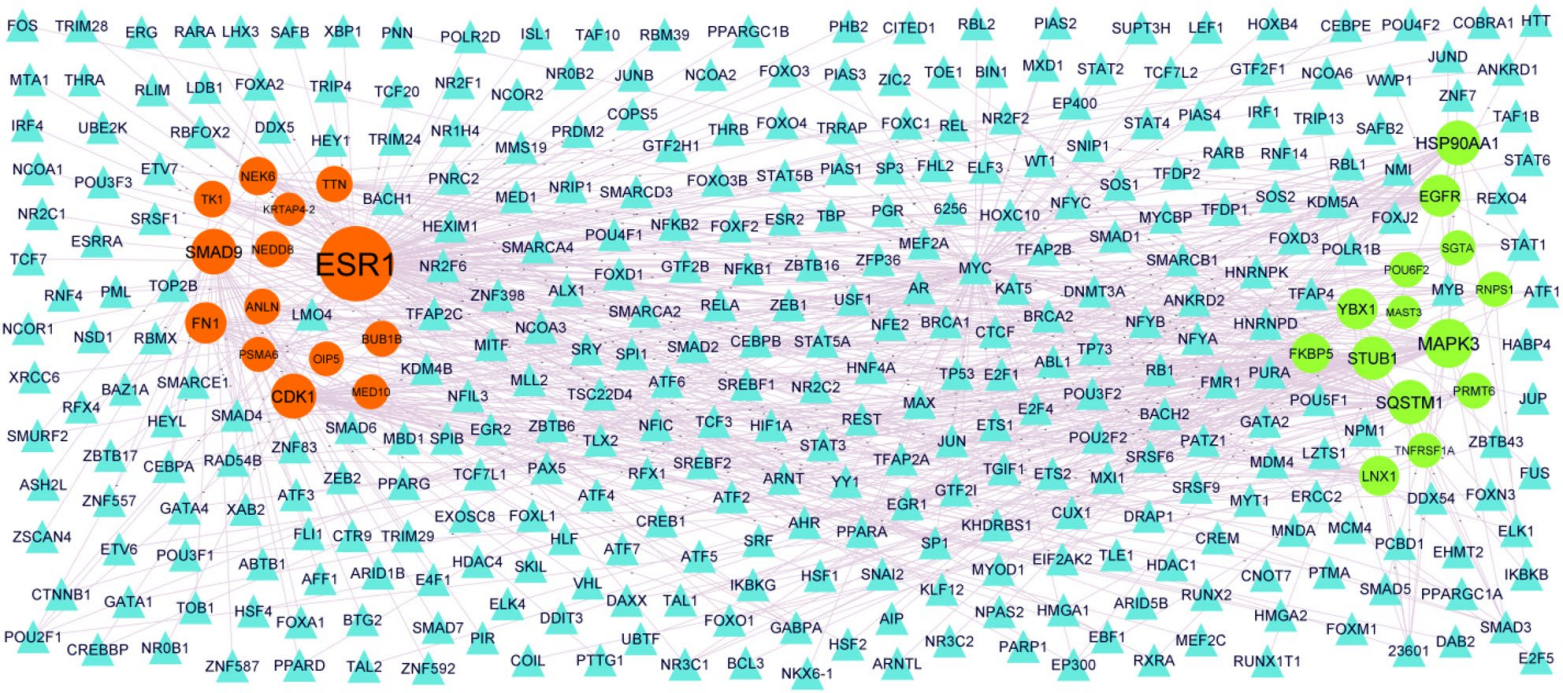
TF—hub gene regulatory network. The blue color triangle nodes represent the key TFs; up regulated genes are marked in green; down regulated genes are marked in red.

The network of TFs and predicted targets (hub genes) is presented in Table 4. Based on the TFs, a TF -hub gene regulatory network was constructed with 899 nodes (TF: 604; hub gene: 295) and 3542 interaction pairs (Fig. 6). Notably, MAPK3 targeted 48 TFs, including JUND; HSP90AA1 targeted 35 TFs, including HSF2; SQSTM1 targeted 34 TFs, including SMAD4; STUB1 targeted 31 TFs, including ATF6; EGFR targeted 27 TFs, including ELF3; ESR1 targeted 126 TFs, including ELF3; SMAD9 targeted 38 TFs, including ELF3; CDK1 targeted 36 TFs, including ELF3; FN1 targeted 25 TFs, including ELF3; NEK6 targeted 16 TFs, including ELF3.

### Validation of hub genes by receiver operating characteristic curve (ROC) analysis

**Figure 7:**
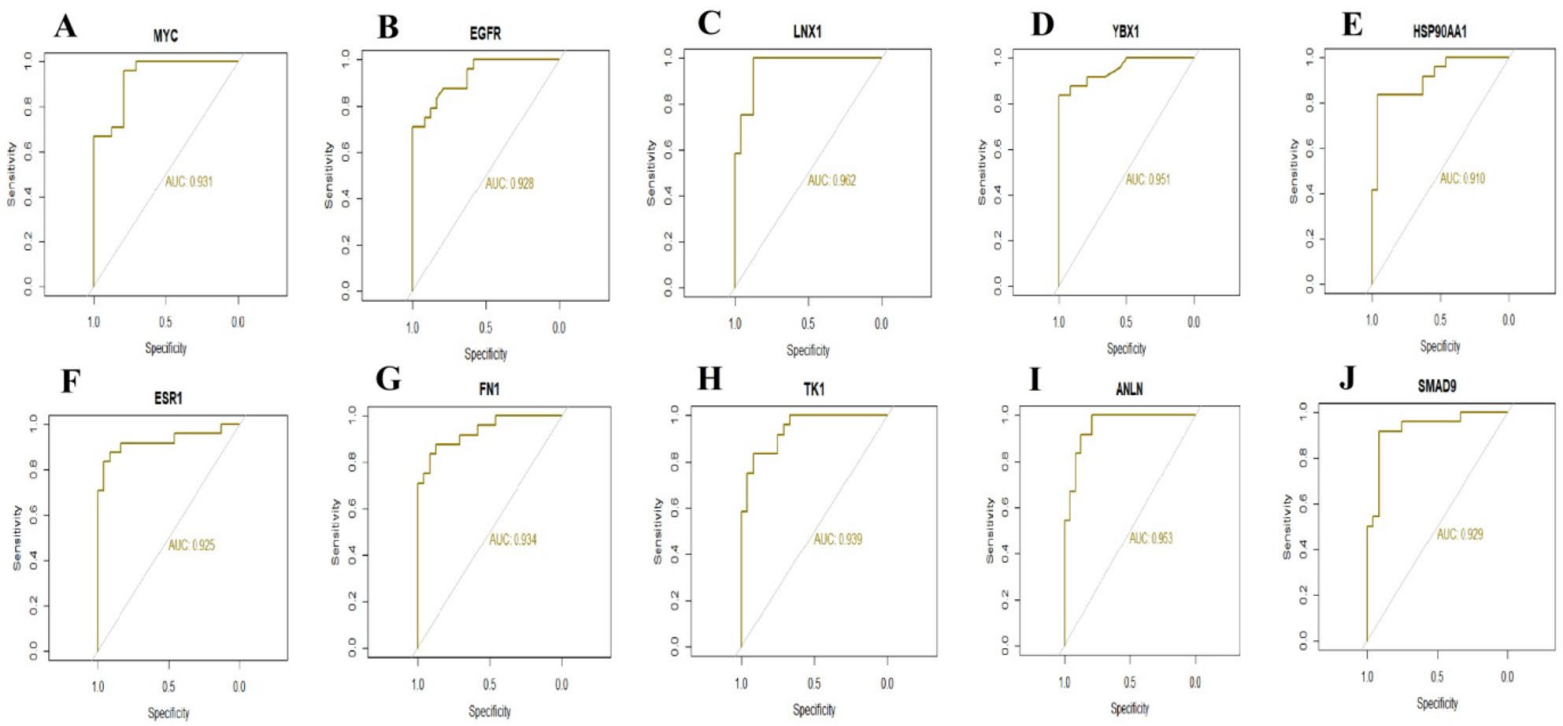
ROC curve validated the sensitivity, specificity of hub genes as a predictive biomarker for dementia prognosis. (**A**) MYC (**B**) EGFR (**C**) LNX1 (**D**) YBX1 (**E**) HSP90AA1 (**F**) ESR1 (**G**) FN1 (**H**) TK1 (**I**) ANLN (**J**) SMAD9.

As these 10 hub genes are prominently expressed in T1DM, we performed a ROC curve analysis to evaluate their sensitivity and specificity for the diagnosis of T1DM. As shown in Fig. 7, MYC, EGFR, LNX1, YBX1, HSP90AA1, ESR1, FN1, TK1, ANLN and SMAD9 achieved an AUC value of > 0.9, demonstrating that these genes have high sensitivity and specificity for T1DM diagnosis. The results suggested that MYC, EGFR, LNX1, YBX1, HSP90AA1, ESR1, FN1, TK1, ANLN and SMAD9 can be used as biomarkers for the diagnosis of T1DM.

## Discussion

T1DM is the common forms of chronic autoimmune diabetes that affect an individual's quality of childhood life^42^. However, the potential causes of T1DM remain uncertain. Understanding the underlying molecular pathogenesis of T1DM is of key importance for diagnosis, prognosis and identifying drug targets. As NGS data can provide information regarding the expression levels of thousands of genes in the human genome simultaneously, this methodology has been widely used to predict the potential diagnostic and therapeutic targets for T1DM. In the present investigation, we analyzed the NGS dataset GSE162689, which includes 27 T1DM samples and 32 normal control samples. We identified 477 up regulated and 475 down regulated genes between T1DM samples and normal control samples using DESeq2 package in R language software. FGA (fibrinogen alpha chain)^43^ and FGB (fibrinogen beta chain)^44^ levels are correlated with disease severity in patients with cardiovascular disease, but these genes might provide new targets for the development of drugs to treat T1DM. IGF2^45^, IAPP (islet amyloid polypeptide)^46^, INS (insulin)^47^ and MAFA (MAF bZIP transcription factor A)^48^ are proved to be involved in T1DM. Altered expression of ADCYAP1 was observed to be associated with the progression of type 2 diabetes mellitus^49^. Gold et al.^50^ reported that CSNK1G1 might be essential for cognitive impairment. Therefore, these genes are might be essential in the advancement of T1DM and its complications.

Furthermore, we investigated the biological functions of these DEGs by using online website, and GO and pathway enrichment analysis. Husemoen et al.^51^, Zhang et al.^52^, Hartz et al.^53^, Słomiński et al.^54^, Johansson et al.^55^, Pan et al.^56^, Lopez-Sanz et al.^57^, Grant^58^, Słomiński et al.^59^, Galán et al.^60^, Jordan et al.^61^, Winkler et al.^62^, Yip et al.^63^, Crookshank et al.^64^, Lempainen et al.^65^, Qu and Polychronakos^66^, Morrison et al.^67^, Zhang et al.^68^, Gerlinger-Romero et al.^69^, Belanger et al.^70^, Dieter et al.^71^, Wanic et al.^72^, Ushijima Wanic et al.^73^, Guo et al.^74^, Davis et al.^75^, Elbarbary et al.^76^, Villasenor et al.^77^, Zhang et al.^78^, Lee et al.^79^, Zhi et al.^80^, Li Calzi et al.^81^, Sebastiani et al.^82^, Cherney et al.^83^, Doggrell^84^ and Yanagihara et al.^85^ studied the clinical and prognostic values of FLG (filaggrin), FGF21, PEMT (phosphatidylethanolamine *N*-methyltransferase) KL (klotho), CEL (carboxyl ester lipase), FOSL2, STAT1, TCF7L2, TP53, EGFR (epidermal growth factor receptor), ETS1, KCNJ8, DEAF1, GCG (glucagon), IKZF4, OAS1, IRS1, ABCG2, FBXO32, PTBP1, BACH2, CNDP2, KLF11, MT1E, DPP4, SLC29A3, RGS16, MAS1, GCGR (glucagon receptor), HLA-C, VASP (vasodilator stimulated phosphoprotein), CCR2, PTGS2, GLP1R and JMJD6 in patients with T1DM. Vassilev et al.^86^, Qin et al.^87^, Ma et al.^88^, West et al.^89^, Hoffmann et al.^90^, Deary et al.^91^, Belangero et al.^92^, Jung et al.^93^, Tang et al.^94^, Goodier et al.^95^, Petyuk et al.^96^, Roux et al.^97^, Castrogiovanni et al.^98^, Suleiman et al.^99^, Haack et al.^100^, Kwiatkowski et al.^101^, Pinacho et al.^102^, Luo et al.^103^, He et al.^104^, Moudi et al.^105^, Thevenon et al.^106^, Li et al.^107^, Reitz et al.^108^, Jenkins and Escayg^109^, Letronne et al.^110^, Ma et al.^111^, Chabbert et al.^112^, Abramsson et al.^113^, Aeby et al.^114^ and Roll et al.^115^ showed the diagnostic values of genes include DCC (DCC netrin 1 receptor), PLP1, SNX19, SH3RF1, TNFRSF1A, NCSTN (nicastrin), DGCR2, NPAS2, CDNF (cerebral dopamine neurotrophic factor), SMCR8, HSPA2, STUB1, CHID1, ATP13A2, SQSTM1, LIG3, SP4, ACSL6, ERN1, ATF6B, LRFN2, NRG3, LRRTM3, GABRA2, ADAM30, GABRR2, TSHZ3, LOXL1, SCN1B and SRPX2 in patients with cognitive impairment. Previous studies have shown that genes include KCP (kielin cysteine rich BMP regulator)^116^, NOG (noggin)^117^, COL6A3^118^, BTG2^119^, RPS6^120^, KLF15^121^, KLF3^122^, ZFP36^123^, ETV5^124^, TLE3^125^, NNMT (nicotinamide N-methyltransferase)^126^, WDTC1^127^, ZFHX3^128^, SIAH2^129^, MBOAT7^130^, RUNX1T1^131^, MAPK4^132^, KLF9^133^, SELENBP1^134^, HELZ2^135^, ELK1^136^, SERTAD2^137^, CRTC3^138^, ABCB11^139^, TACR1^140^, SLC22A11^141^, PER3^142^, P2RX5^143^, MFAP5^144^, FGL1^145^, OLFM4^146^, NTN1^147^, ESR1^148^, ABCB1^149^, VAV3^150^ and LAMB3^151^ can be used as clinical prognostic biomarkers for obesity. Genes include STAR (steroidogenic acute regulatory protein)^152^, IL1RN^153^, AQP5^154^, EGR1^155^, SFTPD (surfactant protein D)^156^, KLF10^157^, PODXL (podocalyxin like)^158^, FOXN3^159^, IL6R^160^, PBX1^161^, APOD (apolipoprotein D)^162^, ACVR2B^163^, CD34^164^, INSR (insulin receptor)^165^, APOA5^166^, STAR (steroidogenic acute regulatory protein)^167^, PDK4^168^, GLS (glutaminase)^169^, FKBP5^170^, SLC6A15^171^, MT2A^172^, SLC38A4^173^, AQP7^174^, ABHD15^175^, ABCA1^176^, ZNRF1^177^, PPP1R3B^178^, MAOA (monoamine oxidase A)^179^, UBE2E2^180^, RNASEK (ribonuclease K)^181^, PREX1^182^, DGKG (diacylglycerol kinase gamma)^183^, POSTN (periostin)^184^, COMP (cartilage oligomeric matrix protein)^185^, GAP43^186^, P2RY12^187^, SELL (selectin L)^188^ and DLG2^189^ were related to type 2 diabetes mellitus. Expression of ERRFI1^190^, ALOX12^191^, SOCS5^192^, DDIT4^193^, DUSP4^194^, IL6ST^195^, DUSP1^196^, SMAD1^197^, NCL (nucleolin)^198^, METTL14^199^, FMOD (fibromodulin)^200^, CYGB (cytoglobin)^201^, UNC5A^202^ and TAAR9^203^ are believed to be associated with diabetic nephropathy. Genes include FAP (fibroblast activation protein alpha)^204^, EYA4^205^, BCL9^206^, IRF2BP2^207^, EGR3^208^, GADD45B^209^, DMD (dystrophin)^210^, LSR (lipolysis stimulated lipoprotein receptor)^211^, DLL4^212^, SUN2^213^, SOS1^214^, PIK3CA^215^, GAMT (guanidinoacetate *N*-methyltransferase)^216^, RBM47^217^, HSP90AA1^218^, GAB1^219^, S1PR1^220^, EDNRB (endothelin receptor type B)^221^, NFKBIA (NFKB inhibitor alpha)^222^, GJA1^223^, GADD45G^224^, PHLDA1^225^, CMPK2^226^, FIGN (fidgetin, microtubule severing factor)^227^, KCNJ2^228^, ABCC9^229^, DIRAS3^230^, EPHX1^231^, RAB4A^232^, UBIAD1^233^, CASQ2^234^, TTN (titin)^235^, KCNH1^236^, JPH2^237^, OXGR1^238^, UCHL1^239^, SERPINA3^240^, MMP28^241^, ADAMTS2^242^, P2RY1^243^, CSF2RA^244^, MYO1F^245^, SELPLG (selectin P ligand)^246^ and SAMHD1^247^ have been reported to be associated with cardiovascular disease. Previous studies had shown that the altered expression of genes include MAOB (monoamine oxidase B)^248^, VEGFC (vascular endothelial growth factor C)^249^, DBP (D-box binding PAR bZIP transcription factor)^250^, MYADM (myeloid associated differentiation marker)^251^, NES (nestin)^252^, SMURF1^253^, EDNRB (endothelin receptor type B)^254^, MUC6^255^, TOR2A^256^, TNKS (tankyrase)^257^, NEDD9^258^, ASIC1^259^, ADAMTS8^260^, DYSF (dysferlin)^261^, SLC26A9^262^, SLC45A3^263^ and KCNQ2^264^ were closely related to the occurrence of hypertension. Yang et al.^265^, Zhang et al.^266^ and Wang et al.^267^ revealed that genes include SYVN1, BTG1 and CFB (complement factor B) might be the potential targets for diabetic retinopathy diagnosis and treatment. Study indicating that these enriched genes might play important roles in the progression of T1DM.

Construction of PPI network of DEGs may be favorable for understanding the relationship of advancing T1DM. The results of the present investigation might provide potential biomarkers for the diagnosis of T1DM. SMAD9 plays an important role in the development of hypertension^268^. Our results indicate the importance of this hub gene might be involved in occurrence and development of T1DM. MYC (MYC proto-oncogene, bHLH transcription factor), LNX1, YBX1, FN1, TK1 and ANLN (anillin actin binding protein) are likely to provide new potential biomarkers for clinical practice or treatment of T1DM with further research.

In this investigation, the miRNA-hub gene regulatory network and TF-hub gene regulatory network that regulates T1DM was constructed. CDK1^269^, hsa-mir-199b-3p^270^, JUND^271^ and FOXF2^272^ are a promising biomarkers in obesity detection and diagnosis. Hsa-mir-106a-5p^273^, hsa-mir-206^274^, SMAD4^275^ and ATF6^276^ biomarkers were confirmed in type 2 diabetes mellitus progression. Hsa-mir-106a-5p^277^ and HSF2^278^ have been shown to promote cardiovascular disease.. Mendes-Silva et al.^279^ reported that hsa-mir-664a-3p promotes cognitive impairment. Some scholars pointed out that ELF3 was involved in the pathogenesis of diabetic nephropathy^280^. Previous studies have shown that SRY is involved in the development of hypertension^281^. Our results showed that these hub genes, miRNAs and TFs are might be involved in progression of T1DM. Together, RNPS1, MAPK3, NEK6, hsa-mir-4677-3p, hsa-mir-3125, hsa-mir-4779, hsa-mir-548az-3p, hsa-mir-5688, hsa-mir-6512-3p, XAB2, KHDRBS1 and RELA might be effective targets in T1DM, but more experimental investigations and clinical trials are needed.

In conclusion, the study used a comprehensive bioinformatics analysis methods to identify DEGs, as well as unique biological functions and pathways of T1DM, thereby enhancing the current understanding of the molecular pathogenesis of T1DM. Moreover, these results might provide potential biomarkers for the initial and proper diagnosis of T1DM, as well as potential therapeutic targets for the advancementof novel T1DM treatments.

## Supplementary Information

Supplementary Table S1.

## Data Availability

The datasets supporting the conclusions of this article are available in the GEO (Gene Expression Omnibus) (https://www.ncbi.nlm.nih.gov/geo/) repository. [(GSE162689) (https://www.ncbi.nlm.nih.gov/geo/query/acc.cgi?acc=GSE162689].
