# Supplementary material for: Identification of candidate biomarkers and pathways associated with type 1 diabetes mellitus using bioinformatics analysis
Source: Sci Rep. 2022 Jun 1;12:9157. doi: 10.1038/s41598-022-13291-1 (PMC9160069; doi:10.1038/s41598-022-13291-1)
Supplement: Supplementary file 1 — Supplementary Table S1. [file 41598_2022_13291_MOESM1_ESM.docx]

**Supplementary Table S1** The statistical metrics for key differentially expressed genes (DEGs)

| **Gene Symbol** | **logFC** | **pValue** | **adj.P.Val** | **tvalue** | **Regulation** | **Gene Name** |
| --- | --- | --- | --- | --- | --- | --- |
| CRNN | 5.712946 | 1.99E-09 | 6.00E-07 | 5.998709 | Up | cornulin |
| PGA5 | 4.614794 | 6.37E-06 | 0.000351 | 4.513665 | Up | pepsinogen A5 |
| PGA4 | 4.56097 | 6.52E-06 | 0.000356 | 4.508769 | Up | pepsinogen A4 |
| PGA3 | 4.488203 | 2.38E-06 | 0.00017 | 4.718 | Up | pepsinogen A3 |
| CGA | 4.197712 | 1.53E-05 | 0.000659 | 4.324569 | Up | glycoprotein hormones, alpha polypeptide |
| FGA | 4.148471 | 0.000107 | 0.002674 | 3.873135 | Up | fibrinogen alpha chain |
| IGF2 | 3.675622 | 1.58E-06 | 0.000129 | 4.800531 | Up | insulin like growth factor 2 |
| KRT6A | 3.542048 | 3.39E-06 | 0.000219 | 4.645643 | Up | keratin 6A |
| FGB | 3.450811 | 0.00024 | 0.004787 | 3.6727 | Up | fibrinogen beta chain |
| LCE3D | 3.269781 | 4.62E-09 | 1.14E-06 | 5.860339 | Up | late cornified envelope 3D |
| PRR9 | 3.141513 | 2.53E-08 | 4.72E-06 | 5.571309 | Up | proline rich 9 |
| SLC6A15 | 3.082853 | 0.004861 | 0.038891 | 2.816137 | Up | solute carrier family 6 member 15 |
| APOA5 | 3.070951 | 6.13E-06 | 0.000342 | 4.521718 | Up | apolipoprotein A5 |
| SPRR3 | 2.937684 | 1.25E-05 | 0.000573 | 4.368603 | Up | small proline rich protein 3 |
| FLG | 2.932836 | 0.000863 | 0.01179 | 3.331797 | Up | filaggrin |
| SPRR2D | 2.867184 | 0.000152 | 0.003454 | 3.787481 | Up | small proline rich protein 2D |
| SPRR1B | 2.866557 | 2.47E-05 | 0.000915 | 4.217563 | Up | small proline rich protein 1B |
| SLITRK6 | 2.858672 | 3.81E-05 | 0.001252 | 4.118929 | Up | SLIT and NTRK like family member 6 |
| CT62 | 2.82068 | 0.000229 | 0.004614 | 3.684932 | Up | cancer/testis associated 62 |
| FGF21 | 2.789926 | 3.91E-06 | 0.000244 | 4.615907 | Up | fibroblast growth factor 21 |
| FGG | 2.773228 | 0.001178 | 0.01471 | 3.244101 | Up | fibrinogen gamma chain |
| TBX22 | 2.752029 | 5.13E-05 | 0.001556 | 4.049842 | Up | T-box transcription factor 22 |
| CALCR | 2.747775 | 0.000411 | 0.006924 | 3.533141 | Up | calcitonin receptor |
| PGC | 2.688901 | 2.74E-05 | 0.000994 | 4.194452 | Up | progastricsin |
| SPRR1A | 2.664413 | 0.000477 | 0.007678 | 3.493077 | Up | small proline rich protein 1A |
| ABCB11 | 2.650492 | 0.004481 | 0.036813 | 2.842169 | Up | ATP binding cassette subfamily B member 11 |
| USH2A | 2.631238 | 0.002995 | 0.027872 | 2.968252 | Up | usherin |
| OTX2 | 2.568693 | 6.44E-06 | 0.000354 | 4.511363 | Up | orthodenticlehomeobox 2 |
| HBM | 2.562338 | 0.002326 | 0.023468 | 3.045118 | Up | hemoglobin subunit mu |
| DCC | 2.561422 | 0.000102 | 0.002565 | 3.885894 | Up | DCC netrin 1 receptor |
| POTED | 2.474492 | 0.002454 | 0.024342 | 3.028922 | Up | POTE ankyrin domain family member D |
| MUC21 | 2.456424 | 0.005692 | 0.043083 | 2.765033 | Up | mucin 21, cell surface associated |
| KCP | 2.393659 | 0.001362 | 0.016053 | 3.202565 | Up | kielin cysteine rich BMP regulator |
| RGPD3 | 2.363618 | 0.003817 | 0.032868 | 2.892895 | Up | RANBP2 like and GRIP domain containing 3 |
| NOG | 2.354832 | 0.000792 | 0.01105 | 3.355497 | Up | noggin |
| STAR | 2.354313 | 0.000103 | 0.002597 | 3.882572 | Up | steroidogenic acute regulatory protein |
| KRT16 | 2.299283 | 0.004488 | 0.036852 | 2.841684 | Up | keratin 16 |
| HSFX2 | 2.284692 | 0.000834 | 0.01146 | 3.341177 | Up | heat shock transcription factor family, X-linked 2 |
| IL1RN | 2.270537 | 0.001478 | 0.017037 | 3.179045 | Up | interleukin 1 receptor antagonist |
| ZSCAN10 | 2.266233 | 0.000319 | 0.005811 | 3.599153 | Up | zinc finger and SCAN domain containing 10 |
| MAGEA2 | 2.264446 | 0.000456 | 0.007479 | 3.505373 | Up | MAGE family member A2 |
| SHISA2 | 2.254679 | 2.83E-05 | 0.001022 | 4.186632 | Up | shisa family member 2 |
| TACR1 | 2.231669 | 0.001599 | 0.017899 | 3.156123 | Up | tachykinin receptor 1 |
| AQP5 | 2.195113 | 0.00015 | 0.003428 | 3.791855 | Up | aquaporin 5 |
| PRRG3 | 2.180564 | 0.005567 | 0.042512 | 2.77226 | Up | proline rich and Gla domain 3 |
| SYNDIG1 | 2.170655 | 0.002504 | 0.024649 | 3.022825 | Up | synapse differentiation inducing 1 |
| TFAP2C | 2.160589 | 0.000396 | 0.006739 | 3.542676 | Up | transcription factor AP-2 gamma |
| ERRFI1 | 2.155407 | 3.75E-08 | 6.56E-06 | 5.5022 | Up | ERBB receptor feedback inhibitor 1 |
| TMEM174 | 2.090104 | 7.82E-05 | 0.002098 | 3.949874 | Up | transmembrane protein 174 |
| CNTNAP4 | 2.08132 | 0.001501 | 0.017188 | 3.174485 | Up | contactin associated protein family member 4 |
| PLP1 | 2.069097 | 0.001613 | 0.017994 | 3.153457 | Up | proteolipid protein 1 |
| RUNX1T1 | 2.059542 | 8.51E-05 | 0.002242 | 3.929478 | Up | RUNX1 partner transcriptional co-repressor 1 |
| ALOX12 | 2.044757 | 0.007045 | 0.049709 | 2.694705 | Up | arachidonate 12-lipoxygenase, 12S type |
| KRT13 | 2.038413 | 0.002945 | 0.027488 | 2.973453 | Up | keratin 13 |
| SLC7A14 | 1.98194 | 0.006299 | 0.0461 | 2.731787 | Up | solute carrier family 7 member 14 |
| SPRR2E | 1.941341 | 0.006326 | 0.046256 | 2.730406 | Up | small proline rich protein 2E |
| PDK4 | 1.930909 | 2.91E-10 | 1.25E-07 | 6.303686 | Up | pyruvate dehydrogenase kinase 4 |
| TENM1 | 1.914484 | 0.006644 | 0.047813 | 2.714165 | Up | teneurintransmembrane protein 1 |
| PRLHR | 1.912351 | 0.006684 | 0.047973 | 2.712197 | Up | prolactin releasing hormone receptor |
| HAO1 | 1.901548 | 0.004154 | 0.03502 | 2.866261 | Up | hydroxyacid oxidase 1 |
| SLC22A11 | 1.873469 | 0.006672 | 0.047915 | 2.7128 | Up | solute carrier family 22 member 11 |
| LOC729080 | 1.856377 | 0.007083 | 0.049857 | 2.692934 | Up | glycine cleavage system protein H (aminomethyl carrier) pseudogene |
| PEMT | 1.849123 | 7.27E-06 | 0.000386 | 4.485648 | Up | phosphatidylethanolamine N-methyltransferase |
| LY6H | 1.840253 | 0.0016 | 0.017903 | 3.155867 | Up | lymphocyte antigen 6 family member H |
| FAM205A | 1.836628 | 0.004029 | 0.034239 | 2.875852 | Up | family with sequence similarity 205 member A |
| TMEM236 | 1.814489 | 0.000914 | 0.012245 | 3.315815 | Up | transmembrane protein 236 |
| KCNJ13 | 1.809864 | 0.004278 | 0.035664 | 2.856913 | Up | potassium inwardly rectifying channel subfamily J member 13 |
| FAP | 1.803439 | 0.000962 | 0.01274 | 3.301429 | Up | fibroblast activation protein alpha |
| EYA4 | 1.800375 | 0.003266 | 0.029605 | 2.941492 | Up | EYA transcriptional coactivator and phosphatase 4 |
| BTBD11 | 1.791996 | 0.002099 | 0.021686 | 3.075855 | Up | BTB domain containing 11 |
| OR5J2 | 1.786096 | 0.000858 | 0.011727 | 3.333497 | Up | olfactory receptor family 5 subfamily J member 2 |
| FBP2 | 1.783474 | 0.001941 | 0.020527 | 3.099056 | Up | fructose-bisphosphatase 2 |
| LCE3E | 1.78332 | 0.000904 | 0.012149 | 3.318662 | Up | late cornified envelope 3E |
| ZNF554 | 1.777372 | 0.000256 | 0.005002 | 3.656153 | Up | zinc finger protein 554 |
| OR10H1 | 1.769139 | 0.001545 | 0.017516 | 3.166037 | Up | olfactory receptor family 10 subfamily H member 1 |
| AQP7P1 | 1.76578 | 0.000244 | 0.004837 | 3.668581 | Up | aquaporin 7 pseudogene 1 |
| EGR1 | 1.751068 | 8.94E-07 | 7.97E-05 | 4.913629 | Up | early growth response 1 |
| CSGALNACT1 | 1.736673 | 0.000245 | 0.004842 | 3.666956 | Up | chondroitin sulfate N-acetylgalactosaminyltransferase 1 |
| MT2A | 1.736195 | 7.33E-07 | 6.80E-05 | 4.952342 | Up | metallothionein 2A |
| MAL | 1.723595 | 5.94E-06 | 0.000338 | 4.528542 | Up | mal, T cell differentiation protein |
| SLC38A4 | 1.716457 | 0.00026 | 0.00507 | 3.652138 | Up | solute carrier family 38 member 4 |
| GCG | 1.704823 | 0.004274 | 0.035664 | 2.857184 | Up | glucagon |
| OR13D1 | 1.699677 | 0.004653 | 0.037774 | 2.830118 | Up | olfactory receptor family 13 subfamily D member 1 |
| ASIC1 | 1.678655 | 0.00502 | 0.039748 | 2.805723 | Up | acid sensing ion channel subunit 1 |
| MMP16 | 1.671308 | 0.005478 | 0.041991 | 2.777507 | Up | matrix metallopeptidase 16 |
| ADAMTS8 | 1.657804 | 0.005782 | 0.043574 | 2.759899 | Up | ADAM metallopeptidase with thrombospondin type 1 motif 8 |
| PTGER4 | 1.653859 | 3.38E-07 | 3.75E-05 | 5.101075 | Up | prostaglandin E receptor 4 |
| TMEM201 | 1.653235 | 5.59E-10 | 2.16E-07 | 6.201596 | Up | transmembrane protein 201 |
| FLJ40194 | 1.624297 | 0.004189 | 0.035182 | 2.863583 | Up | uncharacterized FLJ40194 |
| SPATA31C2 | 1.606051 | 0.007008 | 0.049527 | 2.696447 | Up | SPATA31 subfamily C member 2 |
| COL6A3 | 1.59849 | 2.57E-08 | 4.74E-06 | 5.56868 | Up | collagen type VI alpha 3 chain |
| MAPK4 | 1.583571 | 0.002391 | 0.023885 | 3.0368 | Up | mitogen-activated protein kinase 4 |
| OAS2 | 1.57667 | 0.002062 | 0.02144 | 3.08113 | Up | 2'-5'-oligoadenylate synthetase 2 |
| AQP7 | 1.564682 | 2.01E-05 | 0.000792 | 4.263552 | Up | aquaporin 7 |
| SLC25A48 | 1.563621 | 0.001616 | 0.017994 | 3.152948 | Up | solute carrier family 25 member 48 |
| ETV4 | 1.562936 | 0.000269 | 0.005148 | 3.643839 | Up | ETS variant transcription factor 4 |
| MAOB | 1.561413 | 1.32E-08 | 2.88E-06 | 5.683282 | Up | monoamine oxidase B |
| C2CD4B | 1.560294 | 1.38E-11 | 9.97E-09 | 6.760282 | Up | C2 calcium dependent domain containing 4B |
| CCDC178 | 1.555319 | 0.006459 | 0.046949 | 2.723504 | Up | coiled-coil domain containing 178 |
| GPC6 | 1.545071 | 0.000569 | 0.008694 | 3.445993 | Up | glypican 6 |
| KCNJ2 | 1.54264 | 0.005658 | 0.042973 | 2.766965 | Up | potassium inwardly rectifying channel subfamily J member 2 |
| OR5D14 | 1.535933 | 0.004061 | 0.034442 | 2.873396 | Up | olfactory receptor family 5 subfamily D member 14 |
| MT1E | 1.527259 | 4.13E-05 | 0.001326 | 4.100352 | Up | metallothionein 1E |
| SOCS5 | 1.523522 | 0.002001 | 0.02099 | 3.090019 | Up | suppressor of cytokine signaling 5 |
| GMNC | 1.517516 | 0.001378 | 0.016174 | 3.199272 | Up | geminin coiled-coil domain containing |
| BCL9 | 1.517476 | 6.32E-05 | 0.00178 | 4.00057 | Up | BCL9 transcription coactivator |
| PDK3 | 1.516039 | 5.57E-05 | 0.001659 | 4.030114 | Up | pyruvate dehydrogenase kinase 3 |
| ABCC9 | 1.508688 | 3.88E-05 | 0.001266 | 4.114451 | Up | ATP binding cassette subfamily C member 9 |
| POU6F2 | 1.507127 | 0.000877 | 0.011878 | 3.327431 | Up | POU class 6 homeobox 2 |
| BTG2 | 1.506976 | 1.08E-05 | 0.000512 | 4.40043 | Up | BTG anti-proliferation factor 2 |
| A4GNT | 1.487207 | 0.006669 | 0.047915 | 2.712924 | Up | alpha-1,4-N-acetylglucosaminyltransferase |
| CST6 | 1.474865 | 0.005179 | 0.040581 | 2.795655 | Up | cystatin E/M |
| CELP | 1.456416 | 0.000752 | 0.010713 | 3.369855 | Up | carboxyl ester lipase pseudogene |
| PRR15 | 1.44211 | 4.79E-05 | 0.001473 | 4.065781 | Up | proline rich 15 |
| VEGFC | 1.439026 | 0.000138 | 0.00323 | 3.81237 | Up | vascular endothelial growth factor C |
| FAM20C | 1.417883 | 3.53E-07 | 3.87E-05 | 5.092481 | Up | FAM20C golgi associated secretory pathway kinase |
| MPZ | 1.412425 | 0.001225 | 0.015005 | 3.232986 | Up | myelin protein zero |
| C16orf89 | 1.406088 | 0.002355 | 0.023671 | 3.041349 | Up | chromosome 16 open reading frame 89 |
| USP27X | 1.405375 | 2.98E-05 | 0.001061 | 4.175094 | Up | ubiquitin specific peptidase 27 X-linked |
| STK32B | 1.401504 | 0.00137 | 0.016106 | 3.200934 | Up | serine/threonine kinase 32B |
| TMEM176A | 1.395841 | 0.001167 | 0.014603 | 3.24678 | Up | transmembrane protein 176A |
| KLK13 | 1.39366 | 0.003137 | 0.028793 | 2.95399 | Up | kallikrein related peptidase 13 |
| RPS6 | 1.393238 | 0.000141 | 0.003288 | 3.806963 | Up | ribosomal protein S6 |
| ID3 | 1.379774 | 0.001032 | 0.01339 | 3.281721 | Up | inhibitor of DNA binding 3, HLH protein |
| PGAP2 | 1.372801 | 0.003315 | 0.029859 | 2.936936 | Up | post-GPI attachment to proteins 2 |
| ALOXE3 | 1.371318 | 0.005208 | 0.040713 | 2.793904 | Up | arachidonatelipoxygenase 3 |
| LSM11 | 1.370033 | 0.000167 | 0.003679 | 3.763746 | Up | LSM11, U7 small nuclear RNA associated |
| BCAP31 | 1.366767 | 0.000323 | 0.005863 | 3.596276 | Up | B cell receptor associated protein 31 |
| DDIT4 | 1.357877 | 2.95E-05 | 0.001054 | 4.177066 | Up | DNA damage inducible transcript 4 |
| DPP4 | 1.354505 | 0.000874 | 0.01186 | 3.328261 | Up | dipeptidyl peptidase 4 |
| TYSND1 | 1.351009 | 1.59E-09 | 5.04E-07 | 6.035046 | Up | trypsin like peroxisomal matrix peptidase 1 |
| IRF2BP2 | 1.34637 | 5.95E-18 | 2.15E-14 | 8.633426 | Up | interferon regulatory factor 2 binding protein 2 |
| KLF15 | 1.343721 | 1.94E-06 | 0.000145 | 4.760024 | Up | Kruppel like factor 15 |
| PCDHB7 | 1.334027 | 0.002902 | 0.027259 | 2.97794 | Up | protocadherin beta 7 |
| EGR3 | 1.31896 | 0.00597 | 0.044535 | 2.749441 | Up | early growth response 3 |
| RNF165 | 1.312414 | 0.004877 | 0.038959 | 2.815046 | Up | ring finger protein 165 |
| GRPR | 1.2879 | 0.000461 | 0.007519 | 3.502487 | Up | gastrin releasing peptide receptor |
| SLC10A5 | 1.281611 | 0.000367 | 0.006373 | 3.562596 | Up | solute carrier family 10 member 5 |
| AQP8 | 1.275576 | 0.000165 | 0.003653 | 3.767345 | Up | aquaporin 8 |
| SLC22A17 | 1.275441 | 0.005167 | 0.040504 | 2.796408 | Up | solute carrier family 22 member 17 |
| PHLDA1 | 1.265 | 7.26E-07 | 6.77E-05 | 4.954344 | Up | pleckstrin homology like domain family A member 1 |
| FAM86B2 | 1.261397 | 0.00011 | 0.002723 | 3.868009 | Up | family with sequence similarity 86 member B2 |
| TNXA | 1.26094 | 0.000548 | 0.008447 | 3.456266 | Up | tenascin XA (pseudogene) |
| LTBP4 | 1.249779 | 1.25E-10 | 6.48E-08 | 6.432767 | Up | latent transforming growth factor beta binding protein 4 |
| IKZF4 | 1.247809 | 0.005701 | 0.043119 | 2.764503 | Up | IKAROS family zinc finger 4 |
| CREB3L1 | 1.240081 | 0.001747 | 0.019045 | 3.13026 | Up | cAMP responsive element binding protein 3 like 1 |
| A1CF | 1.237404 | 0.000302 | 0.00558 | 3.613598 | Up | APOBEC1 complementation factor |
| EMX1 | 1.231921 | 0.007097 | 0.049918 | 2.692267 | Up | empty spiracles homeobox 1 |
| NAT10 | 1.230925 | 0.000288 | 0.005418 | 3.625785 | Up | N-acetyltransferase 10 |
| NETO2 | 1.226436 | 2.78E-05 | 0.001004 | 4.191134 | Up | neuropilin and tolloid like 2 |
| ACSL6 | 1.22516 | 0.000699 | 0.010166 | 3.390161 | Up | acyl-CoA synthetase long chain family member 6 |
| KLF3 | 1.222656 | 2.23E-09 | 6.52E-07 | 5.979866 | Up | Kruppel like factor 3 |
| ZFP36 | 1.222469 | 2.71E-07 | 3.23E-05 | 5.142302 | Up | ZFP36 ring finger protein |
| QDPR | 1.212418 | 0.000429 | 0.007144 | 3.521702 | Up | quinoiddihydropteridinereductase |
| OAS1 | 1.211224 | 0.004602 | 0.03752 | 2.833639 | Up | 2'-5'-oligoadenylate synthetase 1 |
| ETV5 | 1.20819 | 3.13E-06 | 0.00021 | 4.662414 | Up | ETS variant transcription factor 5 |
| GRIK5 | 1.206035 | 0.002239 | 0.022781 | 3.056565 | Up | glutamate ionotropic receptor kainate type subunit 5 |
| KL | 1.201704 | 0.006204 | 0.045712 | 2.736802 | Up | klotho |
| ZNF385C | 1.200196 | 0.00318 | 0.029058 | 2.94975 | Up | zinc finger protein 385C |
| TMEM168 | 1.200047 | 0.001202 | 0.014842 | 3.238427 | Up | transmembrane protein 168 |
| GADD45B | 1.195691 | 6.00E-06 | 0.000339 | 4.526504 | Up | growth arrest and DNA damage inducible beta |
| SLC29A3 | 1.193652 | 1.40E-05 | 0.000618 | 4.344205 | Up | solute carrier family 29 member 3 |
| GPR63 | 1.189122 | 5.77E-05 | 0.001693 | 4.022037 | Up | G protein-coupled receptor 63 |
| MAN1A1 | 1.187174 | 5.53E-06 | 0.000322 | 4.54348 | Up | mannosidase alpha class 1A member 1 |
| NXN | 1.184622 | 3.38E-05 | 0.001161 | 4.146023 | Up | nucleoredoxin |
| CGREF1 | 1.181501 | 0.004649 | 0.037769 | 2.830375 | Up | cell growth regulator with EF-hand domain 1 |
| TLE3 | 1.181041 | 1.46E-09 | 4.91E-07 | 6.048256 | Up | TLE family member 3, transcriptional corepressor |
| RPL8 | 1.175798 | 8.84E-06 | 0.000445 | 4.443715 | Up | ribosomal protein L8 |
| HEYL | 1.175375 | 7.83E-05 | 0.002098 | 3.94967 | Up | hes related family bHLH transcription factor with YRPW motif like |
| PER3 | 1.174824 | 2.57E-06 | 0.00018 | 4.702617 | Up | period circadian regulator 3 |
| CXXC4 | 1.172558 | 0.000526 | 0.008232 | 3.46716 | Up | CXXC finger protein 4 |
| HRAS | 1.17075 | 4.69E-05 | 0.001454 | 4.070447 | Up | HRas proto-oncogene, GTPase |
| GLI3 | 1.168455 | 0.00011 | 0.002728 | 3.866542 | Up | GLI family zinc finger 3 |
| SYCN | 1.16423 | 0.001588 | 0.017817 | 3.158183 | Up | syncollin |
| WDR89 | 1.15871 | 0.003082 | 0.028428 | 2.959443 | Up | WD repeat domain 89 |
| KIAA0408 | 1.156987 | 0.00359 | 0.031565 | 2.912147 | Up | KIAA0408 |
| SFTPD | 1.156467 | 0.000869 | 0.011855 | 3.329763 | Up | surfactant protein D |
| MYC | 1.155459 | 0.000232 | 0.004664 | 3.68131 | Up | MYC proto-oncogene, bHLH transcription factor |
| TTI1 | 1.153438 | 7.67E-06 | 0.000403 | 4.474283 | Up | TELO2 interacting protein 1 |
| IFIT3 | 1.153223 | 0.001484 | 0.017069 | 3.177774 | Up | interferon induced protein with tetratricopeptide repeats 3 |
| IGSF8 | 1.147182 | 3.42E-05 | 0.00117 | 4.143533 | Up | immunoglobulin superfamily member 8 |
| RPRD2 | 1.136951 | 4.05E-09 | 1.04E-06 | 5.882334 | Up | regulation of nuclear pre-mRNA domain containing 2 |
| MT1JP | 1.135989 | 0.002674 | 0.025796 | 3.002974 | Up | metallothionein 1J, pseudogene |
| SNORA81 | 1.134404 | 7.05E-05 | 0.001942 | 3.974527 | Up | small nucleolar RNA, H/ACA box 81 |
| DMD | 1.134394 | 0.0025 | 0.024649 | 3.02331 | Up | dystrophin |
| ZNF416 | 1.12885 | 3.44E-06 | 0.00022 | 4.642339 | Up | zinc finger protein 416 |
| AQP12B | 1.122447 | 0.003687 | 0.032134 | 2.903728 | Up | aquaporin 12B |
| FKBP8 | 1.12091 | 0.000337 | 0.006069 | 3.585204 | Up | FKBP prolylisomerase 8 |
| IRS1 | 1.109776 | 2.50E-09 | 7.07E-07 | 5.961342 | Up | insulin receptor substrate 1 |
| KIAA1958 | 1.104138 | 2.20E-07 | 2.72E-05 | 5.181865 | Up | KIAA1958 |
| RPS28 | 1.099636 | 2.86E-10 | 1.25E-07 | 6.306441 | Up | ribosomal protein S28 |
| TMEM140 | 1.093078 | 0.00016 | 0.003573 | 3.774447 | Up | transmembrane protein 140 |
| DBP | 1.092264 | 1.15E-06 | 9.80E-05 | 4.86439 | Up | D-box binding PAR bZIP transcription factor |
| MUC6 | 1.08831 | 0.005474 | 0.041981 | 2.777718 | Up | mucin 6, oligomeric mucus/gel-forming |
| MTCH1 | 1.088002 | 0.004192 | 0.035182 | 2.86336 | Up | mitochondrial carrier 1 |
| CHST10 | 1.086677 | 0.005118 | 0.040291 | 2.799519 | Up | carbohydrate sulfotransferase 10 |
| ABHD15 | 1.086125 | 0.000312 | 0.005712 | 3.605158 | Up | abhydrolase domain containing 15 |
| KLF9 | 1.080005 | 8.43E-13 | 9.53E-10 | 7.15394 | Up | Kruppel like factor 9 |
| EIF4B | 1.06405 | 0.000817 | 0.011276 | 3.347136 | Up | eukaryotic translation initiation factor 4B |
| KLF10 | 1.055746 | 0.000299 | 0.005546 | 3.616276 | Up | Kruppel like factor 10 |
| PODXL | 1.05305 | 1.75E-06 | 0.000137 | 4.780037 | Up | podocalyxin like |
| BVES | 1.050529 | 0.000813 | 0.011258 | 3.348218 | Up | blood vessel epicardial substance |
| GLTP | 1.050372 | 5.80E-06 | 0.000333 | 4.533591 | Up | glycolipid transfer protein |
| MNT | 1.045232 | 2.72E-05 | 0.000992 | 4.195389 | Up | MAX network transcriptional repressor |
| LSR | 1.044877 | 1.76E-05 | 0.000726 | 4.292801 | Up | lipolysis stimulated lipoprotein receptor |
| CEL | 1.038816 | 0.002503 | 0.024649 | 3.023013 | Up | carboxyl ester lipase |
| NUP205 | 1.036199 | 0.002925 | 0.027371 | 2.975558 | Up | nucleoporin 205 |
| MT1L | 1.036012 | 0.001676 | 0.018469 | 3.142292 | Up | metallothionein 1L, pseudogene |
| FOSL2 | 1.035358 | 3.00E-05 | 0.001065 | 4.173687 | Up | FOS like 2, AP-1 transcription factor subunit |
| ACVR2B-AS1 | 1.03269 | 0.002713 | 0.026028 | 2.998466 | Up | ACVR2B antisense RNA 1 |
| SLC1A5 | 1.032611 | 0.000353 | 0.006238 | 3.572762 | Up | solute carrier family 1 member 5 |
| ZNF362 | 1.03072 | 5.17E-06 | 0.000306 | 4.557695 | Up | zinc finger protein 362 |
| WASF1 | 1.029075 | 9.96E-05 | 0.002531 | 3.891542 | Up | WASP family member 1 |
| PAPSS2 | 1.024653 | 0.003229 | 0.029385 | 2.945045 | Up | 3'-phosphoadenosine 5'-phosphosulfate synthase 2 |
| TMCO4 | 1.023584 | 0.000267 | 0.005139 | 3.645801 | Up | transmembrane and coiled-coil domains 4 |
| KIAA1549 | 1.022711 | 0.000159 | 0.003558 | 3.777019 | Up | KIAA1549 |
| NR1D1 | 1.022533 | 0.002444 | 0.024264 | 3.030221 | Up | nuclear receptor subfamily 1 group D member 1 |
| DUSP4 | 1.013696 | 5.02E-05 | 0.001535 | 4.054499 | Up | dual specificity phosphatase 4 |
| SNX19 | 1.013619 | 3.95E-05 | 0.001287 | 4.110135 | Up | sorting nexin 19 |
| SMCR8 | 1.013255 | 1.35E-05 | 0.000604 | 4.351545 | Up | SMCR8-C9orf72 complex subunit |
| FECH | 1.008972 | 1.98E-05 | 0.000786 | 4.267423 | Up | ferrochelatase |
| FOXN3 | 1.007664 | 3.12E-10 | 1.31E-07 | 6.292462 | Up | forkhead box N3 |
| IL6ST | 1.006029 | 0.002468 | 0.024433 | 3.027296 | Up | interleukin 6 signal transducer |
| IL6R | 0.999744 | 0.006884 | 0.048917 | 2.702392 | Up | interleukin 6 receptor |
| PCDHB6 | 0.994986 | 0.005158 | 0.040486 | 2.796974 | Up | protocadherin beta 6 |
| ZNF581 | 0.993708 | 0.000106 | 0.00265 | 3.876665 | Up | zinc finger protein 581 |
| KLF6 | 0.991792 | 6.78E-07 | 6.43E-05 | 4.967609 | Up | Kruppel like factor 6 |
| CPA1 | 0.99028 | 0.003622 | 0.031696 | 2.909338 | Up | carboxypeptidase A1 |
| ABCA1 | 0.989596 | 0.000135 | 0.003174 | 3.817307 | Up | ATP binding cassette subfamily A member 1 |
| ZFP36L1 | 0.983112 | 0.000597 | 0.009015 | 3.432766 | Up | ZFP36 ring finger protein like 1 |
| PBX1 | 0.979927 | 4.02E-13 | 5.59E-10 | 7.254949 | Up | PBX homeobox 1 |
| DYSF | 0.977559 | 0.006334 | 0.046301 | 2.729951 | Up | dysferlin |
| SH3RF1 | 0.977474 | 0.000206 | 0.004295 | 3.711432 | Up | SH3 domain containing ring finger 1 |
| PLEKHG6 | 0.975335 | 0.000311 | 0.0057 | 3.605994 | Up | pleckstrin homology and RhoGEF domain containing G6 |
| URM1 | 0.974945 | 1.43E-08 | 3.05E-06 | 5.670336 | Up | ubiquitin related modifier 1 |
| ERN1 | 0.974217 | 0.000112 | 0.002749 | 3.86398 | Up | endoplasmic reticulum to nucleus signaling 1 |
| RPL18 | 0.972875 | 1.59E-06 | 0.000129 | 4.799987 | Up | ribosomal protein L18 |
| CNTN3 | 0.966787 | 0.001627 | 0.018064 | 3.151099 | Up | contactin 3 |
| CYGB | 0.960228 | 4.03E-05 | 0.001303 | 4.105482 | Up | cytoglobin |
| RNF24 | 0.952932 | 0.000767 | 0.010872 | 3.364507 | Up | ring finger protein 24 |
| ABCG2 | 0.942728 | 0.002598 | 0.025251 | 3.011741 | Up | ATP binding cassette subfamily G member 2 (Junior blood group) |
| PRRC1 | 0.940958 | 1.44E-05 | 0.000633 | 4.337711 | Up | proline rich coiled-coil 1 |
| NHEJ1 | 0.940305 | 0.005191 | 0.040636 | 2.794938 | Up | non-homologous end joining factor 1 |
| PDK2 | 0.936816 | 1.05E-05 | 0.000505 | 4.406224 | Up | pyruvate dehydrogenase kinase 2 |
| BOC | 0.933366 | 6.19E-06 | 0.000345 | 4.519766 | Up | BOC cell adhesion associated, oncogene regulated |
| PNPLA4 | 0.92827 | 1.98E-09 | 6.00E-07 | 5.999168 | Up | patatin like phospholipase domain containing 4 |
| SELENBP1 | 0.925116 | 5.37E-07 | 5.49E-05 | 5.012682 | Up | selenium binding protein 1 |
| STAT1 | 0.92016 | 2.19E-08 | 4.26E-06 | 5.596302 | Up | signal transducer and activator of transcription 1 |
| TMC4 | 0.918004 | 7.50E-06 | 0.000396 | 4.479012 | Up | transmembrane channel like 4 |
| HSPA2 | 0.917681 | 0.000234 | 0.004701 | 3.678748 | Up | heat shock protein family A (Hsp70) member 2 |
| DUSP1 | 0.916798 | 6.01E-06 | 0.000339 | 4.526156 | Up | dual specificity phosphatase 1 |
| CLSTN2 | 0.915169 | 0.000756 | 0.010747 | 3.368549 | Up | calsyntenin 2 |
| DIP2B | 0.914675 | 3.23E-05 | 0.001126 | 4.156545 | Up | disco interacting protein 2 homolog B |
| FMOD | 0.902186 | 0.001186 | 0.01473 | 3.242196 | Up | fibromodulin |
| SNORA46 | 0.900951 | 0.006185 | 0.045618 | 2.737784 | Up | small nucleolar RNA, H/ACA box 46 |
| MYADM | 0.899156 | 9.01E-06 | 0.000447 | 4.439634 | Up | myeloid associated differentiation marker |
| ARPC1A | 0.896149 | 8.17E-09 | 1.92E-06 | 5.764985 | Up | actin related protein 2/3 complex subunit 1A |
| PCDHB11 | 0.894232 | 0.006266 | 0.045933 | 2.733512 | Up | protocadherin beta 11 |
| KCTD12 | 0.89297 | 0.000774 | 0.010938 | 3.361758 | Up | potassium channel tetramerization domain containing 12 |
| APOD | 0.89142 | 0.00345 | 0.030622 | 2.924542 | Up | apolipoprotein D |
| GLS | 0.884954 | 0.00026 | 0.00507 | 3.652426 | Up | glutaminase |
| FBXO32 | 0.881866 | 3.15E-06 | 0.000211 | 4.660468 | Up | F-box protein 32 |
| ST6GALNAC6 | 0.879554 | 2.07E-05 | 0.000809 | 4.257572 | Up | ST6 N-acetylgalactosaminide alpha-2,6-sialyltransferase 6 |
| SRM | 0.878308 | 0.000309 | 0.005677 | 3.607559 | Up | spermidine synthase |
| DIRAS3 | 0.876306 | 0.003122 | 0.028685 | 2.955463 | Up | DIRAS family GTPase 3 |
| NR0B2 | 0.874431 | 7.41E-05 | 0.002024 | 3.962782 | Up | nuclear receptor subfamily 0 group B member 2 |
| NHS | 0.87443 | 0.005302 | 0.041077 | 2.788104 | Up | NHS actin remodeling regulator |
| SYVN1 | 0.872096 | 0.000139 | 0.003248 | 3.810686 | Up | synoviolin 1 |
| ARL6IP1 | 0.872037 | 0.001505 | 0.017219 | 3.173755 | Up | ADP ribosylation factor like GTPase 6 interacting protein 1 |
| TCF7L2 | 0.871267 | 8.46E-06 | 0.000435 | 4.45327 | Up | transcription factor 7 like 2 |
| TMUB1 | 0.869907 | 2.94E-05 | 0.00105 | 4.178279 | Up | transmembrane and ubiquitin like domain containing 1 |
| SNHG9 | 0.868128 | 0.000191 | 0.004072 | 3.730146 | Up | small nucleolar RNA host gene 9 |
| DHRS11 | 0.86627 | 5.84E-08 | 9.11E-06 | 5.423684 | Up | dehydrogenase/reductase 11 |
| MRPL37 | 0.864054 | 1.60E-06 | 0.000129 | 4.79874 | Up | mitochondrial ribosomal protein L37 |
| DLL4 | 0.858948 | 0.003304 | 0.029823 | 2.937894 | Up | delta like canonical Notch ligand 4 |
| HELZ2 | 0.856157 | 8.62E-05 | 0.002263 | 3.926561 | Up | helicase with zinc finger 2 |
| NNMT | 0.851608 | 0.001206 | 0.014883 | 3.237453 | Up | nicotinamide N-methyltransferase |
| SMAD1 | 0.850932 | 1.92E-07 | 2.46E-05 | 5.206868 | Up | SMAD family member 1 |
| TP53 | 0.850773 | 0.000201 | 0.004226 | 3.717618 | Up | tumor protein p53 |
| PCDH18 | 0.850737 | 0.000247 | 0.004858 | 3.665575 | Up | protocadherin 18 |
| FAM160B2 | 0.848165 | 0.00339 | 0.030322 | 2.929974 | Up | family with sequence similarity 160 member B2 |
| ELK1 | 0.84679 | 7.59E-05 | 0.002058 | 3.957072 | Up | ETS transcription factor ELK1 |
| SUN2 | 0.846146 | 4.47E-08 | 7.36E-06 | 5.471101 | Up | Sad1 and UNC84 domain containing 2 |
| TMEM150A | 0.84345 | 7.57E-07 | 6.88E-05 | 4.946241 | Up | transmembrane protein 150A |
| PTBP1 | 0.84235 | 1.45E-05 | 0.000636 | 4.335712 | Up | polypyrimidine tract binding protein 1 |
| SOS1 | 0.842032 | 1.25E-05 | 0.000573 | 4.369507 | Up | SOS Ras/Rac guanine nucleotide exchange factor 1 |
| PRKACA | 0.841424 | 0.000208 | 0.004333 | 3.708612 | Up | protein kinase cAMP-activated catalytic subunit alpha |
| BACH2 | 0.840276 | 0.004124 | 0.034884 | 2.868539 | Up | BTB domain and CNC homolog 2 |
| TOR2A | 0.838785 | 0.000805 | 0.011164 | 3.351168 | Up | torsin family 2 member A |
| CTSF | 0.836129 | 6.47E-06 | 0.000355 | 4.51037 | Up | cathepsin F |
| EGFR | 0.835307 | 0.002303 | 0.023314 | 3.048103 | Up | epidermal growth factor receptor |
| ETS1 | 0.834376 | 0.002812 | 0.0267 | 2.987581 | Up | ETS proto-oncogene 1, transcription factor |
| PIK3CA | 0.834263 | 9.81E-06 | 0.000476 | 4.421306 | Up | phosphatidylinositol-4,5-bisphosphate 3-kinase catalytic subunit alpha |
| ZNRF1 | 0.834005 | 0.000348 | 0.006201 | 3.57648 | Up | zinc and ring finger 1 |
| CMPK2 | 0.833442 | 0.000382 | 0.006568 | 3.552511 | Up | cytidine/uridine monophosphate kinase 2 |
| C17orf49 | 0.831035 | 6.49E-08 | 9.94E-06 | 5.404842 | Up | chromosome 17 open reading frame 49 |
| GUK1 | 0.830906 | 0.005863 | 0.043941 | 2.755329 | Up | guanylate kinase 1 |
| TSHZ1 | 0.830001 | 0.001412 | 0.016467 | 3.192239 | Up | teashirt zinc finger homeobox 1 |
| ZNF326 | 0.829871 | 2.09E-05 | 0.000816 | 4.255297 | Up | zinc finger protein 326 |
| MED13L | 0.825296 | 1.99E-05 | 0.000788 | 4.266458 | Up | mediator complex subunit 13L |
| GCNT2 | 0.824275 | 0.006687 | 0.047973 | 2.712061 | Up | glucosaminyl (N-acetyl) transferase 2 (I blood group) |
| SAE1 | 0.821934 | 1.76E-05 | 0.000726 | 4.292772 | Up | SUMO1 activating enzyme subunit 1 |
| GTPBP8 | 0.820509 | 1.28E-06 | 0.000107 | 4.842977 | Up | GTP binding protein 8 (putative) |
| PKN1 | 0.820314 | 9.18E-05 | 0.002377 | 3.911368 | Up | protein kinase N1 |
| TNFRSF1A | 0.819517 | 8.65E-06 | 0.000442 | 4.448543 | Up | TNF receptor superfamily member 1A |
| ELOVL1 | 0.818479 | 2.24E-06 | 0.000163 | 4.730554 | Up | ELOVL fatty acid elongase 1 |
| TUT1 | 0.81736 | 0.000101 | 0.002556 | 3.887148 | Up | terminal uridylyltransferase 1, U6 snRNA-specific |
| NCSTN | 0.814539 | 5.60E-10 | 2.16E-07 | 6.201177 | Up | nicastrin |
| MFAP2 | 0.814276 | 0.004538 | 0.037128 | 2.838125 | Up | microfibril associated protein 2 |
| YBX1 | 0.812937 | 9.16E-05 | 0.002377 | 3.911852 | Up | Y-box binding protein 1 |
| PPP1R3B | 0.810696 | 0.000614 | 0.009215 | 3.425208 | Up | protein phosphatase 1 regulatory subunit 3B |
| PLCE1 | 0.80763 | 0.005261 | 0.040902 | 2.790596 | Up | phospholipase C epsilon 1 |
| NDST1 | 0.801304 | 1.38E-10 | 6.95E-08 | 6.417856 | Up | N-deacetylase and N-sulfotransferase 1 |
| KCNJ8 | 0.800647 | 0.000248 | 0.004871 | 3.664642 | Up | potassium inwardly rectifying channel subfamily J member 8 |
| ATF6B | 0.795539 | 5.53E-06 | 0.000322 | 4.543457 | Up | activating transcription factor 6 beta |
| GAMT | 0.794851 | 0.000283 | 0.00535 | 3.630634 | Up | guanidinoacetate N-methyltransferase |
| DGCR2 | 0.792202 | 0.000263 | 0.005112 | 3.649418 | Up | DiGeorge syndrome critical region gene 2 |
| ME2 | 0.792173 | 0.002342 | 0.02356 | 3.043104 | Up | malic enzyme 2 |
| SNORA70F | 0.791991 | 0.001262 | 0.015252 | 3.22447 | Up | small nucleolar RNA, H/ACA box 70F |
| TPI1 | 0.791213 | 0.000701 | 0.010178 | 3.389207 | Up | triosephosphateisomerase 1 |
| JMJD8 | 0.790139 | 3.01E-05 | 0.001068 | 4.172555 | Up | jumonji domain containing 8 |
| NES | 0.788137 | 1.56E-05 | 0.000667 | 4.320476 | Up | nestin |
| CDC42EP3 | 0.787657 | 0.006527 | 0.047324 | 2.72008 | Up | CDC42 effector protein 3 |
| SECISBP2L | 0.785942 | 0.002747 | 0.026216 | 2.994768 | Up | SECIS binding protein 2 like |
| PRPF8 | 0.785467 | 4.09E-05 | 0.001317 | 4.102281 | Up | pre-mRNA processing factor 8 |
| DNAJC4 | 0.784209 | 1.19E-06 | 0.000101 | 4.856749 | Up | DnaJ heat shock protein family (Hsp40) member C4 |
| EIF3B | 0.783198 | 4.65E-05 | 0.001445 | 4.072672 | Up | eukaryotic translation initiation factor 3 subunit B |
| EPHX1 | 0.78283 | 0.000166 | 0.003661 | 3.766555 | Up | epoxide hydrolase 1 |
| WDTC1 | 0.782558 | 8.41E-06 | 0.000434 | 4.454475 | Up | WD and tetratricopeptide repeats 1 |
| MAML1 | 0.78192 | 2.54E-05 | 0.000933 | 4.210975 | Up | mastermind like transcriptional coactivator 1 |
| PCDHB4 | 0.779944 | 0.00151 | 0.01726 | 3.172676 | Up | protocadherin beta 4 |
| CRNKL1 | 0.776112 | 0.001354 | 0.015976 | 3.204332 | Up | crooked neck pre-mRNA splicing factor 1 |
| NFIB | 0.775622 | 0.005204 | 0.040705 | 2.794109 | Up | nuclear factor I B |
| MAPK3 | 0.774729 | 6.63E-06 | 0.000361 | 4.505204 | Up | mitogen-activated protein kinase 3 |
| SLC23A2 | 0.771981 | 0.005612 | 0.042695 | 2.769626 | Up | solute carrier family 23 member 2 |
| RPL18A | 0.771037 | 0.000204 | 0.004258 | 3.713912 | Up | ribosomal protein L18a |
| FAM83B | 0.769782 | 0.000435 | 0.007223 | 3.51776 | Up | family with sequence similarity 83 member B |
| CNDP2 | 0.768381 | 0.000586 | 0.008901 | 3.43821 | Up | carnosinedipeptidase 2 |
| SERTAD2 | 0.768202 | 3.98E-05 | 0.001293 | 4.108585 | Up | SERTA domain containing 2 |
| RCE1 | 0.76665 | 0.000172 | 0.003741 | 3.757149 | Up | Ras converting CAAX endopeptidase 1 |
| C2orf69 | 0.766464 | 8.05E-05 | 0.002142 | 3.942914 | Up | chromosome 2 open reading frame 69 |
| FOXK1 | 0.764006 | 0.001585 | 0.0178 | 3.158636 | Up | forkhead box K1 |
| ZNF646 | 0.763811 | 0.000536 | 0.00834 | 3.462129 | Up | zinc finger protein 646 |
| CAMSAP3 | 0.762877 | 1.95E-05 | 0.000779 | 4.271089 | Up | calmodulin regulated spectrin associated protein family member 3 |
| ACVR2B | 0.761578 | 2.49E-05 | 0.000919 | 4.215416 | Up | activin A receptor type 2B |
| ATXN7L1 | 0.761216 | 0.001983 | 0.020842 | 3.09281 | Up | ataxin 7 like 1 |
| LRRC8B | 0.761015 | 0.000468 | 0.007577 | 3.49819 | Up | leucine rich repeat containing 8 VRAC subunit B |
| PLOD3 | 0.760059 | 3.88E-05 | 0.001266 | 4.114231 | Up | procollagen-lysine,2-oxoglutarate 5-dioxygenase 3 |
| FAM193B | 0.758019 | 1.78E-05 | 0.000726 | 4.291384 | Up | family with sequence similarity 193 member B |
| ZFHX3 | 0.757752 | 0.001036 | 0.013414 | 3.280609 | Up | zinc finger homeobox 3 |
| SMURF1 | 0.756591 | 1.06E-05 | 0.000508 | 4.404419 | Up | SMAD specific E3 ubiquitin protein ligase 1 |
| AOX1 | 0.756086 | 0.003594 | 0.031565 | 2.911801 | Up | aldehyde oxidase 1 |
| RPS15 | 0.755554 | 6.99E-07 | 6.59E-05 | 4.961597 | Up | ribosomal protein S15 |
| PRMT6 | 0.753247 | 0.001239 | 0.01511 | 3.22983 | Up | protein arginine methyltransferase 6 |
| TRIM24 | 0.752928 | 1.16E-05 | 0.000543 | 4.384086 | Up | tripartite motif containing 24 |
| PRKCSH | 0.750891 | 0.000284 | 0.005365 | 3.629653 | Up | protein kinase C substrate 80K-H |
| ETV6 | 0.75061 | 9.73E-07 | 8.46E-05 | 4.896989 | Up | ETS variant transcription factor 6 |
| GSK3A | 0.7503 | 0.000194 | 0.004091 | 3.727348 | Up | glycogen synthase kinase 3 alpha |
| WDR74 | 0.749277 | 0.000605 | 0.009092 | 3.429532 | Up | WD repeat domain 74 |
| KCNC4 | 0.746517 | 0.002407 | 0.023989 | 3.03483 | Up | potassium voltage-gated channel subfamily C member 4 |
| LARP6 | 0.745789 | 7.94E-05 | 0.002124 | 3.946332 | Up | La ribonucleoprotein 6, translational regulator |
| BCOR | 0.744144 | 0.00012 | 0.002934 | 3.845436 | Up | BCL6 corepressor |
| PSD4 | 0.741924 | 0.005215 | 0.040726 | 2.793473 | Up | pleckstrin and Sec7 domain containing 4 |
| URB1 | 0.74192 | 6.05E-05 | 0.00174 | 4.01084 | Up | URB1 ribosome biogenesis homolog |
| RAB4A | 0.740681 | 0.000763 | 0.010829 | 3.365815 | Up | RAB4A, member RAS oncogene family |
| ZNF341 | 0.740369 | 0.002638 | 0.025544 | 3.007083 | Up | zinc finger protein 341 |
| SGTA | 0.740279 | 0.000162 | 0.003593 | 3.77241 | Up | small glutamine rich tetratricopeptide repeat containing alpha |
| STUB1 | 0.739139 | 0.000973 | 0.012833 | 3.298143 | Up | STIP1 homology and U-box containing protein 1 |
| SLC35E1 | 0.736682 | 9.91E-09 | 2.30E-06 | 5.732304 | Up | solute carrier family 35 member E1 |
| RSBN1 | 0.73582 | 4.22E-08 | 7.07E-06 | 5.481284 | Up | round spermatid basic protein 1 |
| NPAS2 | 0.732705 | 3.23E-05 | 0.001126 | 4.156955 | Up | neuronal PAS domain protein 2 |
| MAOA | 0.731781 | 4.31E-06 | 0.000262 | 4.595946 | Up | monoamine oxidase A |
| USP1 | 0.728983 | 0.003174 | 0.029017 | 2.950339 | Up | ubiquitin specific peptidase 1 |
| RBM47 | 0.728968 | 1.56E-08 | 3.21E-06 | 5.654557 | Up | RNA binding motif protein 47 |
| RNF43 | 0.728384 | 0.000997 | 0.013032 | 3.291369 | Up | ring finger protein 43 |
| KIAA0930 | 0.728341 | 6.95E-05 | 0.001924 | 3.977861 | Up | KIAA0930 |
| SHISA5 | 0.72744 | 0.000573 | 0.008736 | 3.444214 | Up | shisa family member 5 |
| TCOF1 | 0.727002 | 0.00054 | 0.008367 | 3.460175 | Up | treacle ribosome biogenesis factor 1 |
| NCL | 0.725941 | 0.002185 | 0.022354 | 3.063829 | Up | nucleolin |
| FKBP5 | 0.724631 | 7.85E-06 | 0.000411 | 4.469326 | Up | FKBP prolylisomerase 5 |
| RBM22 | 0.723796 | 3.19E-06 | 0.000211 | 4.6585 | Up | RNA binding motif protein 22 |
| SLC48A1 | 0.720957 | 2.30E-05 | 0.000874 | 4.233151 | Up | solute carrier family 48 member 1 |
| GNL1 | 0.720727 | 0.000309 | 0.005676 | 3.607864 | Up | G protein nucleolar 1 (putative) |
| FOXP1 | 0.720205 | 1.58E-09 | 5.04E-07 | 6.036016 | Up | forkhead box P1 |
| TRAPPC3 | 0.719608 | 0.000551 | 0.008489 | 3.45471 | Up | trafficking protein particle complex 3 |
| METTL14 | 0.718111 | 0.000565 | 0.008669 | 3.447662 | Up | methyltransferase like 14 |
| PIGM | 0.716435 | 0.000194 | 0.004091 | 3.727242 | Up | phosphatidylinositol glycan anchor biosynthesis class M |
| MZT2B | 0.714175 | 0.001677 | 0.018471 | 3.142085 | Up | mitotic spindle organizing protein 2B |
| ATF7IP | 0.712488 | 4.27E-09 | 1.07E-06 | 5.873211 | Up | activating transcription factor 7 interacting protein |
| RWDD2B | 0.711793 | 0.000232 | 0.00466 | 3.681821 | Up | RWD domain containing 2B |
| POLR1D | 0.708737 | 0.005643 | 0.042875 | 2.767845 | Up | RNA polymerase I and III subunit D |
| UBIAD1 | 0.706576 | 8.09E-06 | 0.00042 | 4.46291 | Up | UbiAprenyltransferase domain containing 1 |
| SFXN2 | 0.700426 | 0.001682 | 0.018509 | 3.141299 | Up | sideroflexin 2 |
| FIGN | 0.699523 | 3.68E-05 | 0.001226 | 4.126718 | Up | fidgetin, microtubule severing factor |
| UBAP2 | 0.699319 | 3.77E-08 | 6.56E-06 | 5.50114 | Up | ubiquitin associated protein 2 |
| UBE2E2 | 0.699156 | 5.75E-05 | 0.001693 | 4.022695 | Up | ubiquitin conjugating enzyme E2 E2 |
| MX1 | 0.698814 | 0.003319 | 0.029859 | 2.936555 | Up | MX dynamin like GTPase 1 |
| SPPL3 | 0.697852 | 0.000696 | 0.010145 | 3.390959 | Up | signal peptide peptidase like 3 |
| KCTD11 | 0.697791 | 2.01E-05 | 0.000792 | 4.263333 | Up | potassium channel tetramerization domain containing 11 |
| HSP90AA1 | 0.696743 | 0.00019 | 0.00406 | 3.731557 | Up | heat shock protein 90 alpha family class A member 1 |
| BTG1 | 0.694132 | 4.22E-06 | 0.000257 | 4.600208 | Up | BTG anti-proliferation factor 1 |
| GALM | 0.693238 | 0.005154 | 0.040486 | 2.797233 | Up | galactosemutarotase |
| GAB1 | 0.693194 | 1.58E-08 | 3.21E-06 | 5.652613 | Up | GRB2 associated binding protein 1 |
| PXMP2 | 0.692523 | 0.001283 | 0.015424 | 3.21973 | Up | peroxisomal membrane protein 2 |
| SCARNA17 | 0.691261 | 8.34E-06 | 0.000432 | 4.456247 | Up | small Cajal body-specific RNA 17 |
| S1PR1 | 0.691074 | 6.64E-05 | 0.001855 | 3.988707 | Up | sphingosine-1-phosphate receptor 1 |
| THRB | 0.691029 | 0.000885 | 0.011987 | 3.324668 | Up | thyroid hormone receptor beta |
| RPL39 | 0.690209 | 0.005561 | 0.042488 | 2.77258 | Up | ribosomal protein L39 |
| UTP14A | 0.6899 | 0.003424 | 0.030472 | 2.926834 | Up | UTP14A small subunit processome component |
| EDNRB | 0.689734 | 0.000237 | 0.004752 | 3.6754 | Up | endothelin receptor type B |
| CRTC3 | 0.689519 | 8.27E-05 | 0.002191 | 3.936548 | Up | CREB regulated transcription coactivator 3 |
| AK1 | 0.689426 | 0.001162 | 0.014574 | 3.248124 | Up | adenylate kinase 1 |
| PTGER3 | 0.687494 | 0.001956 | 0.020638 | 3.09677 | Up | prostaglandin E receptor 3 |
| SYBU | 0.687308 | 0.000239 | 0.004773 | 3.674039 | Up | syntabulin |
| MED25 | 0.687197 | 0.002406 | 0.023989 | 3.034873 | Up | mediator complex subunit 25 |
| ERP29 | 0.686161 | 0.000267 | 0.005139 | 3.645521 | Up | endoplasmic reticulum protein 29 |
| RBM23 | 0.685812 | 0.000216 | 0.004436 | 3.699217 | Up | RNA binding motif protein 23 |
| GPATCH4 | 0.684195 | 0.001189 | 0.014747 | 3.241437 | Up | G-patch domain containing 4 |
| CDNF | 0.684189 | 0.001318 | 0.015708 | 3.212028 | Up | cerebral dopamine neurotrophic factor |
| RNASEK | 0.683852 | 0.005382 | 0.041449 | 2.783259 | Up | ribonuclease K |
| LPCAT3 | 0.683539 | 0.000328 | 0.005946 | 3.591945 | Up | lysophosphatidylcholineacyltransferase 3 |
| CASC3 | 0.683245 | 0.000342 | 0.006117 | 3.581073 | Up | CASC3 exon junction complex subunit |
| MTMR3 | 0.682235 | 0.000873 | 0.01186 | 3.328634 | Up | myotubularin related protein 3 |
| TNKS | 0.681959 | 0.000955 | 0.012674 | 3.303437 | Up | tankyrase |
| SF3A1 | 0.681792 | 0.000107 | 0.002666 | 3.87484 | Up | splicing factor 3a subunit 1 |
| CHID1 | 0.681692 | 2.35E-05 | 0.000888 | 4.228751 | Up | chitinase domain containing 1 |
| AGPAT3 | 0.679929 | 4.02E-06 | 0.000249 | 4.610538 | Up | 1-acylglycerol-3-phosphate O-acyltransferase 3 |
| ADPRHL1 | 0.67793 | 0.003299 | 0.029823 | 2.93839 | Up | ADP-ribosylhydrolase like 1 |
| MEX3C | 0.676707 | 1.74E-07 | 2.28E-05 | 5.225485 | Up | mex-3 RNA binding family member C |
| SIAH2 | 0.676628 | 4.59E-05 | 0.001437 | 4.075423 | Up | siah E3 ubiquitin protein ligase 2 |
| ACD | 0.674822 | 0.000397 | 0.006739 | 3.541993 | Up | ACD shelterin complex subunit and telomerase recruitment factor |
| NFKBIA | 0.671989 | 2.49E-09 | 7.07E-07 | 5.961923 | Up | NFKB inhibitor alpha |
| CDC42EP4 | 0.670577 | 3.53E-05 | 0.001193 | 4.136046 | Up | CDC42 effector protein 4 |
| ARHGAP17 | 0.670458 | 0.000353 | 0.006238 | 3.572609 | Up | Rho GTPase activating protein 17 |
| GJA1 | 0.669532 | 0.000191 | 0.004071 | 3.730543 | Up | gap junction protein alpha 1 |
| RNPS1 | 0.669286 | 0.000469 | 0.007577 | 3.498063 | Up | RNA binding protein with serine rich domain 1 |
| ATP13A2 | 0.668974 | 0.001969 | 0.020738 | 3.094808 | Up | ATPase cation transporting 13A2 |
| ZSWIM3 | 0.666665 | 0.005783 | 0.043574 | 2.759838 | Up | zinc finger SWIM-type containing 3 |
| TXNDC5 | 0.666561 | 6.22E-05 | 0.001775 | 4.004346 | Up | thioredoxin domain containing 5 |
| PPFIBP2 | 0.66328 | 0.00471 | 0.038127 | 2.826221 | Up | PPFIA binding protein 2 |
| MRPL49 | 0.661083 | 4.76E-05 | 0.001473 | 4.066891 | Up | mitochondrial ribosomal protein L49 |
| SLC35C2 | 0.660269 | 0.002623 | 0.025458 | 3.008774 | Up | solute carrier family 35 member C2 |
| CD34 | 0.656903 | 0.001941 | 0.020527 | 3.099122 | Up | CD34 molecule |
| SQSTM1 | 0.656832 | 0.000903 | 0.012146 | 3.319117 | Up | sequestosome 1 |
| DEAF1 | 0.656044 | 0.000482 | 0.007723 | 3.490807 | Up | DEAF1 transcription factor |
| CPNE8 | 0.655935 | 0.006745 | 0.048175 | 2.709168 | Up | copine 8 |
| INSR | 0.655878 | 0.001522 | 0.017355 | 3.170368 | Up | insulin receptor |
| RPL19 | 0.655743 | 0.0001 | 0.002543 | 3.889412 | Up | ribosomal protein L19 |
| NR1D2 | 0.655727 | 0.001279 | 0.015407 | 3.220621 | Up | nuclear receptor subfamily 1 group D member 2 |
| SLC25A43 | 0.655559 | 0.000873 | 0.01186 | 3.32843 | Up | solute carrier family 25 member 43 |
| AGBL5 | 0.654572 | 0.003824 | 0.032915 | 2.892293 | Up | ATP/GTP binding protein like 5 |
| SLC39A4 | 0.653952 | 0.005292 | 0.041019 | 2.788697 | Up | solute carrier family 39 member 4 |
| SP2 | 0.652169 | 0.002758 | 0.026271 | 2.993532 | Up | Sp2 transcription factor |
| SLC2A4RG | 0.65117 | 0.00165 | 0.018241 | 3.146991 | Up | SLC2A4 regulator |
| YIPF3 | 0.650173 | 3.26E-05 | 0.001131 | 4.15422 | Up | Yip1 domain family member 3 |
| MRPS18B | 0.649811 | 7.15E-06 | 0.000383 | 4.489106 | Up | mitochondrial ribosomal protein S18B |
| NEDD9 | 0.648825 | 0.000492 | 0.007837 | 3.485031 | Up | neural precursor cell expressed, developmentally down-regulated 9 |
| GADD45G | 0.648736 | 0.006811 | 0.048569 | 2.705943 | Up | growth arrest and DNA damage inducible gamma |
| ZNRF3 | 0.648723 | 0.001296 | 0.015533 | 3.216764 | Up | zinc and ring finger 3 |
| MAST3 | 0.648387 | 0.001533 | 0.017433 | 3.168306 | Up | microtubule associated serine/threonine kinase 3 |
| SNX12 | 0.648357 | 0.001185 | 0.014729 | 3.242554 | Up | sorting nexin 12 |
| TMED5 | 0.647804 | 4.52E-05 | 0.001421 | 4.079333 | Up | transmembrane p24 trafficking protein 5 |
| BAZ2A | 0.647031 | 0.000218 | 0.004449 | 3.69755 | Up | bromodomain adjacent to zinc finger domain 2A |
| CCDC86 | 0.646936 | 0.000592 | 0.008966 | 3.435136 | Up | coiled-coil domain containing 86 |
| MBOAT7 | 0.644983 | 0.005086 | 0.040095 | 2.801514 | Up | membrane bound O-acyltransferase domain containing 7 |
| GID8 | 0.644926 | 0.00015 | 0.003436 | 3.790262 | Up | GID complex subunit 8 homolog |
| DCPS | 0.642919 | 0.002036 | 0.021241 | 3.084943 | Up | decapping enzyme, scavenger |
| LIG3 | 0.642442 | 5.43E-05 | 0.001633 | 4.036185 | Up | DNA ligase 3 |
| BSG | 0.641779 | 0.001141 | 0.014369 | 3.253151 | Up | basigin (Ok blood group) |
| SP4 | 0.640664 | 0.000992 | 0.012988 | 3.292727 | Up | Sp4 transcription factor |
| KLF11 | 0.63968 | 1.52E-05 | 0.000659 | 4.325257 | Up | Kruppel like factor 11 |
| CIZ1 | 0.63856 | 1.87E-05 | 0.000756 | 4.279502 | Up | CDKN1A interacting zinc finger protein 1 |
| LNX1 | 0.636517 | 1.47E-05 | 0.000641 | 4.333615 | Up | ligand of numb-protein X 1 |
| RALB | 0.63579 | 3.37E-06 | 0.000219 | 4.646621 | Up | RAS like proto-oncogene B |
| BTF3 | 0.632977 | 0.000199 | 0.004195 | 3.720016 | Up | basic transcription factor 3 |
| ZNF836 | 0.632392 | 0.002077 | 0.021526 | 3.078926 | Up | zinc finger protein 836 |
| NET1 | 0.631653 | 0.005749 | 0.043387 | 2.761785 | Up | neuroepithelial cell transforming 1 |
| IAPP | -10.801 | 2.63E-45 | 2.38E-41 | -14.126 | Down | islet amyloid polypeptide |
| INS | -10.3513 | 7.60E-52 | 1.37E-47 | -15.1498 | Down | insulin |
| HAPLN4 | -6.9522 | 3.78E-09 | 1.00E-06 | -5.89366 | Down | hyaluronan and proteoglycan link protein 4 |
| ADCYAP1 | -6.37952 | 7.01E-16 | 1.81E-12 | -8.07029 | Down | adenylatecyclase activating polypeptide 1 |
| CSNK1G1 | -6.37349 | 5.07E-14 | 8.34E-11 | -7.53001 | Down | casein kinase 1 gamma 1 |
| MAFA | -6.08612 | 2.10E-12 | 1.90E-09 | -7.02742 | Down | MAF bZIP transcription factor A |
| IGLL5 | -5.95682 | 0.00111 | 0.014081 | -3.26108 | Down | immunoglobulin lambda like polypeptide 5 |
| WSCD2 | -5.89111 | 1.55E-09 | 5.04E-07 | -6.03927 | Down | WSC domain containing 2 |
| PLCH2 | -5.70065 | 1.54E-15 | 3.49E-12 | -7.9734 | Down | phospholipase C eta 2 |
| SLCO1A2 | -5.65472 | 9.18E-05 | 0.002377 | -3.9112 | Down | solute carrier organic anion transporter family member 1A2 |
| HHATL | -5.54001 | 4.92E-11 | 3.23E-08 | -6.57335 | Down | hedgehog acyltransferase like |
| DMBT1 | -5.11154 | 0.000502 | 0.007945 | -3.47993 | Down | deleted in malignant brain tumors 1 |
| RGS16 | -4.81121 | 1.53E-18 | 9.23E-15 | -8.78743 | Down | regulator of G protein signaling 16 |
| PREX1 | -4.79312 | 5.00E-11 | 3.23E-08 | -6.57103 | Down | phosphatidylinositol-3,4,5-trisphosphate dependent Rac exchange factor 1 |
| SYT16 | -4.64825 | 1.25E-08 | 2.80E-06 | -5.69211 | Down | synaptotagmin 16 |
| ASB9 | -4.60995 | 1.08E-17 | 3.26E-14 | -8.56492 | Down | ankyrin repeat and SOCS box containing 9 |
| LRFN2 | -4.54487 | 1.77E-05 | 0.000726 | -4.29175 | Down | leucine rich repeat and fibronectin type III domain containing 2 |
| NRG3 | -4.53264 | 3.24E-05 | 0.001126 | -4.15624 | Down | neuregulin 3 |
| HSD17B13 | -4.51785 | 1.90E-09 | 5.93E-07 | -6.00595 | Down | hydroxysteroid 17-beta dehydrogenase 13 |
| DLK1 | -4.43907 | 2.20E-12 | 1.90E-09 | -7.02104 | Down | delta like non-canonical Notch ligand 1 |
| LOC100286922 | -4.09518 | 0.00035 | 0.00622 | -3.57543 | Down | DnaJ heat shock protein family (Hsp40) member B3 pseudogene |
| LRRTM3 | -4.05763 | 3.63E-06 | 0.00023 | -4.63163 | Down | leucine rich repeat transmembrane neuronal 3 |
| GABRA2 | -3.9965 | 1.21E-06 | 0.000101 | -4.85468 | Down | gamma-aminobutyric acid type A receptor subunit alpha2 |
| GTSF1 | -3.96587 | 8.59E-06 | 0.00044 | -4.44996 | Down | gametocyte specific factor 1 |
| NTNG2 | -3.89902 | 1.78E-07 | 2.30E-05 | -5.22131 | Down | netrin G2 |
| GLRA1 | -3.83003 | 4.47E-05 | 0.001413 | -4.08161 | Down | glycine receptor alpha 1 |
| DGKG | -3.7646 | 8.17E-13 | 9.53E-10 | -7.15833 | Down | diacylglycerol kinase gamma |
| ISX | -3.75152 | 0.001191 | 0.014761 | -3.24097 | Down | intestine specific homeobox |
| OR10J3 | -3.70449 | 1.79E-06 | 0.000139 | -4.77542 | Down | olfactory receptor family 10 subfamily J member 3 |
| SLC6A17 | -3.70444 | 1.83E-05 | 0.00074 | -4.28474 | Down | solute carrier family 6 member 17 |
| DACH2 | -3.67639 | 4.10E-08 | 6.95E-06 | -5.48656 | Down | dachshund family transcription factor 2 |
| VPREB1 | -3.6609 | 0.001658 | 0.018315 | -3.14545 | Down | V-set pre-B cell surrogate light chain 1 |
| RXRG | -3.64353 | 3.01E-06 | 0.000204 | -4.67042 | Down | retinoid X receptor gamma |
| IFNA16 | -3.63676 | 0.00089 | 0.012039 | -3.32325 | Down | interferon alpha 16 |
| G6PC2 | -3.62269 | 3.42E-06 | 0.00022 | -4.64385 | Down | glucose-6-phosphatase catalytic subunit 2 |
| OR4F5 | -3.59416 | 0.001252 | 0.015226 | -3.22678 | Down | olfactory receptor family 4 subfamily F member 5 |
| SLC18A2 | -3.57954 | 7.56E-09 | 1.80E-06 | -5.77806 | Down | solute carrier family 18 member A2 |
| BIRC8 | -3.56786 | 5.34E-05 | 0.001609 | -4.04035 | Down | baculoviral IAP repeat containing 8 |
| KLHL1 | -3.55633 | 6.13E-05 | 0.001753 | -4.00789 | Down | kelch like family member 1 |
| SLC26A9 | -3.53742 | 0.000256 | 0.005002 | -3.65613 | Down | solute carrier family 26 member 9 |
| OR6C74 | -3.52371 | 0.000373 | 0.006457 | -3.55827 | Down | olfactory receptor family 6 subfamily C member 74 |
| DKK4 | -3.51465 | 3.70E-05 | 0.001229 | -4.12522 | Down | dickkopf WNT signaling pathway inhibitor 4 |
| UNC5A | -3.5005 | 3.25E-06 | 0.000214 | -4.65467 | Down | unc-5 netrin receptor A |
| OR5L1 | -3.49176 | 0.000203 | 0.004248 | -3.71483 | Down | olfactory receptor family 5 subfamily L member 1 |
| LOC154761 | -3.48046 | 1.69E-05 | 0.000707 | -4.30203 | Down | family with sequence similarity 115, member C pseudogene |
| OR4D2 | -3.47937 | 0.001687 | 0.018539 | -3.14047 | Down | olfactory receptor family 4 subfamily D member 2 |
| KCNG3 | -3.45067 | 5.90E-07 | 5.87E-05 | -4.99431 | Down | potassium voltage-gated channel modifier subfamily G member 3 |
| ARHGEF35 | -3.42973 | 0.00378 | 0.032645 | -2.89592 | Down | Rho guanine nucleotide exchange factor 35 |
| SLC27A6 | -3.42698 | 0.00025 | 0.004903 | -3.66238 | Down | solute carrier family 27 member 6 |
| OR2T33 | -3.40998 | 0.000418 | 0.007022 | -3.52868 | Down | olfactory receptor family 2 subfamily T member 33 |
| KIF4B | -3.39216 | 0.000503 | 0.00795 | -3.47931 | Down | kinesin family member 4B |
| CLDN25 | -3.35937 | 0.000658 | 0.009732 | -3.40632 | Down | claudin 25 |
| OR2T6 | -3.33501 | 0.000203 | 0.004248 | -3.71558 | Down | olfactory receptor family 2 subfamily T member 6 |
| EBLN1 | -3.32606 | 0.000711 | 0.010271 | -3.38516 | Down | endogenous Bornavirus like nucleoprotein 1 |
| TKTL2 | -3.32283 | 0.000899 | 0.012131 | -3.32029 | Down | transketolase like 2 |
| ST8SIA5 | -3.29995 | 9.50E-05 | 0.002441 | -3.90304 | Down | ST8 alpha-N-acetyl-neuraminide alpha-2,8-sialyltransferase 5 |
| ADAM30 | -3.29013 | 0.003501 | 0.030982 | -2.91998 | Down | ADAM metallopeptidase domain 30 |
| CDHR1 | -3.24111 | 0.000721 | 0.010362 | -3.38165 | Down | cadherin related family member 1 |
| GJA10 | -3.22733 | 0.000173 | 0.003765 | -3.75491 | Down | gap junction protein alpha 10 |
| CYP3A43 | -3.20066 | 3.99E-05 | 0.001294 | -4.10804 | Down | cytochrome P450 family 3 subfamily A member 43 |
| BMP5 | -3.18774 | 4.18E-06 | 0.000255 | -4.60225 | Down | bone morphogenetic protein 5 |
| PRUNE2 | -3.17519 | 1.38E-09 | 4.71E-07 | -6.05767 | Down | prune homolog 2 with BCH domain |
| LINC00602 | -3.17107 | 0.000318 | 0.005801 | -3.60012 | Down | long intergenic non-protein coding RNA 602 |
| RASD1 | -3.16012 | 1.06E-09 | 3.74E-07 | -6.10078 | Down | ras related dexamethasone induced 1 |
| TAAR9 | -3.13989 | 0.003702 | 0.032231 | -2.90248 | Down | trace amine associated receptor 9 |
| OR8H2 | -3.12734 | 0.000427 | 0.007125 | -3.5231 | Down | olfactory receptor family 8 subfamily H member 2 |
| INSRR | -3.11972 | 0.005572 | 0.042533 | -2.77196 | Down | insulin receptor related receptor |
| POSTN | -3.11052 | 1.26E-07 | 1.75E-05 | -5.28454 | Down | periostin |
| OR4N2 | -3.09015 | 0.00604 | 0.044854 | -2.74562 | Down | olfactory receptor family 4 subfamily N member 2 |
| CASQ2 | -3.08655 | 0.001058 | 0.013583 | -3.27466 | Down | calsequestrin 2 |
| LOC441204 | -3.08411 | 7.24E-05 | 0.001987 | -3.96836 | Down | uncharacterized LOC441204 |
| OR2A25 | -3.08173 | 0.000264 | 0.005116 | -3.64842 | Down | olfactory receptor family 2 subfamily A member 25 |
| OR5E1P | -3.0489 | 0.001755 | 0.019094 | -3.12882 | Down | olfactory receptor family 5 subfamily E member 1 pseudogene |
| LY6G6E | -3.04857 | 0.001493 | 0.017122 | -3.17595 | Down | lymphocyte antigen 6 family member G6E |
| SSTR5-AS1 | -3.0425 | 8.27E-07 | 7.44E-05 | -4.92887 | Down | SSTR5 antisense RNA 1 |
| CYP24A1 | -3.03607 | 0.002253 | 0.022897 | -3.0547 | Down | cytochrome P450 family 24 subfamily A member 1 |
| TAS2R7 | -3.02863 | 0.000145 | 0.003362 | -3.79889 | Down | taste 2 receptor member 7 |
| SLC8A1-AS1 | -3.02422 | 0.000815 | 0.011262 | -3.34768 | Down | SLC8A1 antisense RNA 1 |
| ZNF385D | -3.02118 | 7.51E-07 | 6.88E-05 | -4.94777 | Down | zinc finger protein 385D |
| LINC00460 | -3.01407 | 0.001244 | 0.015147 | -3.22856 | Down | long intergenic non-protein coding RNA 460 |
| KIF20A | -3.01006 | 0.000681 | 0.009971 | -3.39723 | Down | kinesin family member 20A |
| HLA-DQA1 | -3.00395 | 1.85E-08 | 3.64E-06 | -5.6257 | Down | major histocompatibility complex, class II, DQ alpha 1 |
| ASB5 | -3.00067 | 0.000216 | 0.004436 | -3.69951 | Down | ankyrin repeat and SOCS box containing 5 |
| OR1E2 | -2.99957 | 0.001083 | 0.013816 | -3.26804 | Down | olfactory receptor family 1 subfamily E member 2 |
| TNFAIP8 | -2.99257 | 8.45E-15 | 1.70E-11 | -7.7607 | Down | TNF alpha induced protein 8 |
| OR2B3 | -2.98546 | 0.000293 | 0.005479 | -3.62108 | Down | olfactory receptor family 2 subfamily B member 3 |
| SLC45A3 | -2.97833 | 4.34E-05 | 0.001375 | -4.08865 | Down | solute carrier family 45 member 3 |
| IFNA10 | -2.97627 | 0.000425 | 0.007112 | -3.52402 | Down | interferon alpha 10 |
| VWA5B1 | -2.95932 | 0.001384 | 0.016227 | -3.19796 | Down | von Willebrand factor A domain containing 5B1 |
| USP26 | -2.95706 | 0.002713 | 0.026028 | -2.99855 | Down | ubiquitin specific peptidase 26 |
| TM6SF2 | -2.95528 | 2.99E-08 | 5.40E-06 | -5.54201 | Down | transmembrane 6 superfamily member 2 |
| PPM1E | -2.94698 | 8.01E-06 | 0.000417 | -4.46499 | Down | protein phosphatase, Mg2+/Mn2+ dependent 1E |
| FOXM1 | -2.94119 | 0.003185 | 0.029075 | -2.94926 | Down | forkhead box M1 |
| TPX2 | -2.93595 | 0.000949 | 0.012618 | -3.30526 | Down | TPX2 microtubule nucleation factor |
| LRRC10 | -2.93393 | 0.002164 | 0.022223 | -3.06669 | Down | leucine rich repeat containing 10 |
| GREM2 | -2.92963 | 0.002908 | 0.027299 | -2.97732 | Down | gremlin 2, DAN family BMP antagonist |
| OR6N1 | -2.92321 | 0.00263 | 0.025499 | -3.00796 | Down | olfactory receptor family 6 subfamily N member 1 |
| EIF3IP1 | -2.92189 | 0.003394 | 0.030322 | -2.92956 | Down | eukaryotic translation initiation factor 3 subunit I pseudogene 1 |
| CAPN8 | -2.91404 | 0.000476 | 0.007661 | -3.49417 | Down | calpain 8 |
| GOLGA8CP | -2.90644 | 0.006607 | 0.047649 | -2.71601 | Down | golgin A8 family member C, pseudogene |
| TFF3 | -2.89791 | 8.85E-06 | 0.000445 | -4.44363 | Down | trefoil factor 3 |
| OR4K5 | -2.89759 | 0.000785 | 0.011002 | -3.35799 | Down | olfactory receptor family 4 subfamily K member 5 |
| HOPX | -2.89043 | 5.18E-06 | 0.000306 | -4.55731 | Down | HOP homeobox |
| MTRNR2L5 | -2.88837 | 0.000627 | 0.009365 | -3.41948 | Down | MT-RNR2 like 5 |
| COMP | -2.88387 | 9.00E-07 | 7.98E-05 | -4.9123 | Down | cartilage oligomeric matrix protein |
| OR4A5 | -2.88266 | 0.000521 | 0.008166 | -3.46976 | Down | olfactory receptor family 4 subfamily A member 5 |
| SULT1C2P1 | -2.87268 | 0.000168 | 0.003688 | -3.76253 | Down | sulfotransferase family 1C member 2 pseudogene 1 |
| OR5AN1 | -2.86497 | 0.005374 | 0.041436 | -2.78374 | Down | olfactory receptor family 5 subfamily AN member 1 |
| KRTAP1-3 | -2.86473 | 0.002933 | 0.027411 | -2.97471 | Down | keratin associated protein 1-3 |
| OR5AS1 | -2.8631 | 0.00075 | 0.010703 | -3.37055 | Down | olfactory receptor family 5 subfamily AS member 1 |
| GABRR2 | -2.85852 | 0.001493 | 0.017122 | -3.17608 | Down | gamma-aminobutyric acid type A receptor subunit rho2 |
| OR13G1 | -2.85436 | 0.005275 | 0.040973 | -2.78976 | Down | olfactory receptor family 13 subfamily G member 1 |
| OR2M5 | -2.8505 | 0.001514 | 0.017284 | -3.17193 | Down | olfactory receptor family 2 subfamily M member 5 |
| HTR3A | -2.82474 | 0.000642 | 0.009519 | -3.41324 | Down | 5-hydroxytryptamine receptor 3A |
| KRTAP13-3 | -2.82267 | 0.002954 | 0.027543 | -2.97253 | Down | keratin associated protein 13-3 |
| OR13C5 | -2.82057 | 7.98E-05 | 0.00213 | -3.9449 | Down | olfactory receptor family 13 subfamily C member 5 |
| OR4C3 | -2.80788 | 0.005491 | 0.042035 | -2.77675 | Down | olfactory receptor family 4 subfamily C member 3 |
| PPIEL | -2.78532 | 4.64E-05 | 0.001444 | -4.07319 | Down | peptidylprolylisomerase E like pseudogene |
| ASB11 | -2.78468 | 0.000245 | 0.004837 | -3.66753 | Down | ankyrin repeat and SOCS box containing 11 |
| CHST8 | -2.78461 | 1.89E-05 | 0.00076 | -4.27789 | Down | carbohydrate sulfotransferase 8 |
| GOLGA6L1 | -2.7816 | 0.000171 | 0.003726 | -3.75848 | Down | golgin A6 family like 1 |
| MAS1 | -2.77551 | 0.00044 | 0.007269 | -3.51512 | Down | MAS1 proto-oncogene, G protein-coupled receptor |
| OR52L1 | -2.76744 | 8.16E-05 | 0.002168 | -3.93961 | Down | olfactory receptor family 52 subfamily L member 1 |
| PBK | -2.76743 | 0.000196 | 0.004138 | -3.72403 | Down | PDZ binding kinase |
| ANXA2P3 | -2.76623 | 0.001772 | 0.019268 | -3.12596 | Down | annexin A2 pseudogene 3 |
| KRTAP4-2 | -2.7586 | 0.001037 | 0.013422 | -3.28017 | Down | keratin associated protein 4-2 |
| OR8K1 | -2.75811 | 0.002112 | 0.021778 | -3.07408 | Down | olfactory receptor family 8 subfamily K member 1 |
| TCN1 | -2.75785 | 0.002731 | 0.026128 | -2.99649 | Down | transcobalamin 1 |
| KIRREL3 | -2.75601 | 0.000505 | 0.007982 | -3.47799 | Down | kirre like nephrin family adhesion molecule 3 |
| OR6B2 | -2.7546 | 0.003595 | 0.031565 | -2.91164 | Down | olfactory receptor family 6 subfamily B member 2 |
| KRTAP19-1 | -2.738 | 0.000419 | 0.007044 | -3.52758 | Down | keratin associated protein 19-1 |
| KRTAP3-1 | -2.73476 | 0.006721 | 0.048118 | -2.71035 | Down | keratin associated protein 3-1 |
| SLCO5A1 | -2.7339 | 0.000151 | 0.003436 | -3.78966 | Down | solute carrier organic anion transporter family member 5A1 |
| IFNA1 | -2.71621 | 0.003812 | 0.032859 | -2.89328 | Down | interferon alpha 1 |
| KRTAP3-2 | -2.71406 | 0.003974 | 0.033866 | -2.8802 | Down | keratin associated protein 3-2 |
| VENTXP7 | -2.70781 | 0.000793 | 0.011058 | -3.35508 | Down | VENT homeoboxpseudogene 7 |
| LOC341056 | -2.70401 | 0.004623 | 0.037645 | -2.83218 | Down | SUMO1 activating enzyme subunit 1 pseudogene |
| KRTAP9-9 | -2.69101 | 0.005252 | 0.040848 | -2.79118 | Down | keratin associated protein 9-9 |
| OR56A4 | -2.68936 | 0.002123 | 0.021887 | -3.07243 | Down | olfactory receptor family 56 subfamily A member 4 |
| FAM111B | -2.68489 | 0.000288 | 0.005416 | -3.62612 | Down | family with sequence similarity 111 member B |
| PSMA6 | -2.68174 | 3.99E-18 | 1.80E-14 | -8.67908 | Down | proteasome 20S subunit alpha 6 |
| DLGAP5 | -2.68048 | 0.001477 | 0.017037 | -3.17907 | Down | DLG associated protein 5 |
| PFKFB2 | -2.67874 | 2.19E-10 | 9.91E-08 | -6.34724 | Down | 6-phosphofructo-2-kinase/fructose-2,6-biphosphatase 2 |
| OR5B17 | -2.67817 | 0.00413 | 0.034912 | -2.86807 | Down | olfactory receptor family 5 subfamily B member 17 |
| LOC100287036 | -2.66753 | 0.003578 | 0.031512 | -2.91317 | Down | uncharacterized LOC100287036 |
| PCDH7 | -2.66116 | 6.16E-07 | 6.09E-05 | -4.98602 | Down | protocadherin 7 |
| MND1 | -2.65071 | 2.22E-08 | 4.27E-06 | -5.59402 | Down | meiotic nuclear divisions 1 |
| RCAN2 | -2.60302 | 0.001595 | 0.01787 | -3.15677 | Down | regulator of calcineurin 2 |
| TMEM108 | -2.59295 | 5.48E-05 | 0.001641 | -4.03417 | Down | transmembrane protein 108 |
| OR51A2 | -2.58736 | 0.004723 | 0.038181 | -2.82532 | Down | olfactory receptor family 51 subfamily A member 2 |
| P2RX5 | -2.58657 | 0.000125 | 0.003005 | -3.8356 | Down | purinergic receptor P2X 5 |
| SLC22A9 | -2.58464 | 0.00124 | 0.01511 | -3.22949 | Down | solute carrier family 22 member 9 |
| OR5T1 | -2.57736 | 0.003632 | 0.031742 | -2.90848 | Down | olfactory receptor family 5 subfamily T member 1 |
| OR1A1 | -2.57723 | 0.002728 | 0.026111 | -2.99685 | Down | olfactory receptor family 1 subfamily A member 1 |
| OR13C8 | -2.57325 | 0.001945 | 0.020542 | -3.09849 | Down | olfactory receptor family 13 subfamily C member 8 |
| OR13C2 | -2.57298 | 0.005077 | 0.040061 | -2.80213 | Down | olfactory receptor family 13 subfamily C member 2 |
| GAP43 | -2.57224 | 0.001777 | 0.019296 | -3.12518 | Down | growth associated protein 43 |
| MFAP5 | -2.56407 | 0.000759 | 0.010785 | -3.36736 | Down | microfibril associated protein 5 |
| SGPP2 | -2.55582 | 2.11E-05 | 0.00082 | -4.25256 | Down | sphingosine-1-phosphate phosphatase 2 |
| ADAMTSL1 | -2.55426 | 1.36E-07 | 1.87E-05 | -5.27081 | Down | ADAMTS like 1 |
| OR2L1P | -2.55171 | 0.000488 | 0.007806 | -3.48701 | Down | olfactory receptor family 2 subfamily L member 1 pseudogene |
| TPD52L3 | -2.55159 | 0.001889 | 0.020166 | -3.10709 | Down | TPD52 like 3 |
| PABPC1L2A | -2.55147 | 0.006252 | 0.045885 | -2.73428 | Down | poly(A) binding protein cytoplasmic 1 like 2A |
| OR51B4 | -2.54936 | 0.001501 | 0.017188 | -3.17446 | Down | olfactory receptor family 51 subfamily B member 4 |
| LCE1F | -2.54374 | 0.002888 | 0.027195 | -2.97945 | Down | late cornified envelope 1F |
| KCNQ2 | -2.52303 | 0.001454 | 0.016864 | -3.18366 | Down | potassium voltage-gated channel subfamily Q member 2 |
| SFRP4 | -2.52066 | 1.03E-08 | 2.36E-06 | -5.72526 | Down | secreted frizzled related protein 4 |
| KCNF1 | -2.51814 | 0.000113 | 0.002775 | -3.86075 | Down | potassium voltage-gated channel modifier subfamily F member 1 |
| OR2AG2 | -2.51736 | 0.004873 | 0.038957 | -2.8153 | Down | olfactory receptor family 2 subfamily AG member 2 |
| CD1E | -2.51051 | 0.002538 | 0.024876 | -3.01877 | Down | CD1e molecule |
| OR2M7 | -2.50383 | 0.004156 | 0.03502 | -2.86606 | Down | olfactory receptor family 2 subfamily M member 7 |
| OR51L1 | -2.49986 | 0.001702 | 0.018656 | -3.13791 | Down | olfactory receptor family 51 subfamily L member 1 |
| LINC00477 | -2.49804 | 0.004433 | 0.036513 | -2.8456 | Down | long intergenic non-protein coding RNA 477 |
| OR5H1 | -2.49529 | 0.0037 | 0.032227 | -2.90267 | Down | olfactory receptor family 5 subfamily H member 1 |
| FAM83E | -2.49154 | 0.000801 | 0.01113 | -3.35243 | Down | family with sequence similarity 83 member E |
| CRLF1 | -2.48748 | 1.68E-12 | 1.60E-09 | -7.05875 | Down | cytokine receptor like factor 1 |
| STAG3 | -2.48528 | 0.002489 | 0.024577 | -3.0247 | Down | stromal antigen 3 |
| KRTAP21-2 | -2.47732 | 0.001534 | 0.017433 | -3.16815 | Down | keratin associated protein 21-2 |
| OR8G1 | -2.4672 | 0.005221 | 0.04074 | -2.79305 | Down | olfactory receptor family 8 subfamily G member 1 |
| OR6K6 | -2.46634 | 0.006059 | 0.044944 | -2.74455 | Down | olfactory receptor family 6 subfamily K member 6 |
| KRTAP19-2 | -2.4657 | 0.003103 | 0.028539 | -2.95735 | Down | keratin associated protein 19-2 |
| OR10Z1 | -2.45396 | 0.003252 | 0.029509 | -2.94285 | Down | olfactory receptor family 10 subfamily Z member 1 |
| KRTAP10-1 | -2.45298 | 0.006735 | 0.048175 | -2.70969 | Down | keratin associated protein 10-1 |
| DNAH11 | -2.45203 | 0.000344 | 0.006141 | -3.57944 | Down | dynein axonemal heavy chain 11 |
| NPTX2 | -2.44884 | 1.08E-10 | 5.76E-08 | -6.455 | Down | neuronal pentraxin 2 |
| KCNH1 | -2.44466 | 0.001977 | 0.020792 | -3.0937 | Down | potassium voltage-gated channel subfamily H member 1 |
| OR4K1 | -2.43709 | 0.004239 | 0.035455 | -2.85982 | Down | olfactory receptor family 4 subfamily K member 1 |
| OR5T2 | -2.43076 | 0.002914 | 0.027332 | -2.97665 | Down | olfactory receptor family 5 subfamily T member 2 |
| PSMD6-AS2 | -2.42892 | 0.000265 | 0.005132 | -3.64722 | Down | PSMD6 antisense RNA 2 |
| EDARADD | -2.4128 | 9.72E-06 | 0.000474 | -4.42333 | Down | EDAR associated death domain |
| SDR16C5 | -2.41156 | 0.00454 | 0.037128 | -2.838 | Down | short chain dehydrogenase/reductase family 16C member 5 |
| LOC100130673 | -2.41083 | 0.004883 | 0.03897 | -2.81463 | Down | phosphoribosyl pyrophosphate synthetase 2 pseudogene |
| PI16 | -2.40562 | 0.000421 | 0.007072 | -3.52631 | Down | peptidase inhibitor 16 |
| JPH2 | -2.40312 | 0.00471 | 0.038127 | -2.82621 | Down | junctophilin 2 |
| INSC | -2.40292 | 4.17E-06 | 0.000255 | -4.60267 | Down | INSC spindle orientation adaptor protein |
| FN1 | -2.4003 | 9.15E-07 | 8.07E-05 | -4.90913 | Down | fibronectin 1 |
| ACTBL2 | -2.39082 | 0.003746 | 0.03246 | -2.89875 | Down | actin beta like 2 |
| KIF4A | -2.37867 | 0.001252 | 0.015226 | -3.22668 | Down | kinesin family member 4A |
| VSIG10L | -2.37789 | 0.001623 | 0.018046 | -3.15176 | Down | V-set and immunoglobulin domain containing 10 like |
| GPD1 | -2.37721 | 0.002655 | 0.025667 | -3.00509 | Down | glycerol-3-phosphate dehydrogenase 1 |
| C1QL1 | -2.37667 | 0.000214 | 0.004411 | -3.7015 | Down | complement C1q like 1 |
| NPFF | -2.36906 | 8.90E-05 | 0.002312 | -3.91888 | Down | neuropeptide FF-amide peptide precursor |
| OR4N3P | -2.35827 | 0.005904 | 0.044102 | -2.75305 | Down | olfactory receptor family 4 subfamily N member 3 pseudogene |
| OR2A12 | -2.34861 | 0.002721 | 0.026055 | -2.99766 | Down | olfactory receptor family 2 subfamily A member 12 |
| CAPNS2 | -2.34428 | 0.000221 | 0.004497 | -3.69321 | Down | calpain small subunit 2 |
| OR5AK4P | -2.34022 | 0.006512 | 0.04726 | -2.72083 | Down | olfactory receptor family 5 subfamily AK member 4 pseudogene |
| BEND6 | -2.33639 | 0.000272 | 0.005194 | -3.64043 | Down | BEN domain containing 6 |
| YIPF4 | -2.33303 | 6.30E-05 | 0.00178 | -4.0014 | Down | Yip1 domain family member 4 |
| OR1L8 | -2.33257 | 0.005375 | 0.041436 | -2.78365 | Down | olfactory receptor family 1 subfamily L member 8 |
| FAM95B1 | -2.31927 | 0.003903 | 0.033444 | -2.88593 | Down | family with sequence similarity 95 member B1 |
| RDH16 | -2.3132 | 0.00098 | 0.012875 | -3.29622 | Down | retinol dehydrogenase 16 |
| HBD | -2.30048 | 0.006912 | 0.049 | -2.70104 | Down | hemoglobin subunit delta |
| OR1J2 | -2.29925 | 0.005289 | 0.041019 | -2.7889 | Down | olfactory receptor family 1 subfamily J member 2 |
| TAS2R60 | -2.29771 | 0.000463 | 0.007527 | -3.50125 | Down | taste 2 receptor member 60 |
| VSIG4 | -2.29506 | 5.66E-07 | 5.69E-05 | -5.00245 | Down | V-set and immunoglobulin domain containing 4 |
| IGF2BP3 | -2.29121 | 0.000493 | 0.007844 | -3.48429 | Down | insulin like growth factor 2 mRNA binding protein 3 |
| CHRNA10 | -2.28988 | 0.003958 | 0.033807 | -2.88147 | Down | cholinergic receptor nicotinic alpha 10 subunit |
| OR5AP2 | -2.25924 | 0.006852 | 0.048745 | -2.70396 | Down | olfactory receptor family 5 subfamily AP member 2 |
| MEP1B | -2.25724 | 0.004207 | 0.035254 | -2.86219 | Down | meprin A subunit beta |
| UGT1A9 | -2.25439 | 0.00578 | 0.043574 | -2.76 | Down | UDP glucuronosyltransferase family 1 member A9 |
| OR52K1 | -2.24884 | 0.004392 | 0.03629 | -2.84857 | Down | olfactory receptor family 52 subfamily K member 1 |
| CYP26B1 | -2.24879 | 0.000361 | 0.006326 | -3.56691 | Down | cytochrome P450 family 26 subfamily B member 1 |
| FAT3 | -2.24517 | 0.000527 | 0.008238 | -3.46655 | Down | FAT atypical cadherin 3 |
| IL10 | -2.24228 | 0.003217 | 0.029301 | -2.94624 | Down | interleukin 10 |
| TNFRSF11A | -2.24044 | 7.07E-10 | 2.66E-07 | -6.16461 | Down | TNF receptor superfamily member 11a |
| GCGR | -2.23561 | 0.000942 | 0.012562 | -3.30743 | Down | glucagon receptor |
| RERGL | -2.21524 | 6.67E-06 | 0.000361 | -4.50409 | Down | RERG like |
| ZMAT4 | -2.21188 | 0.004388 | 0.03629 | -2.84882 | Down | zinc finger matrin-type 4 |
| CCDC54 | -2.20591 | 0.004509 | 0.036964 | -2.84014 | Down | coiled-coil domain containing 54 |
| OR9A2 | -2.19677 | 0.006953 | 0.049212 | -2.69909 | Down | olfactory receptor family 9 subfamily A member 2 |
| SPAG6 | -2.19033 | 0.006245 | 0.045876 | -2.73463 | Down | sperm associated antigen 6 |
| LOC401127 | -2.18482 | 0.000134 | 0.003165 | -3.81831 | Down | WD repeat domain 5 pseudogene |
| ENTPD3 | -2.18044 | 2.71E-12 | 2.23E-09 | -6.99221 | Down | ectonucleoside triphosphate diphosphohydrolase 3 |
| LCN2 | -2.16225 | 4.27E-05 | 0.001359 | -4.09226 | Down | lipocalin 2 |
| TOP2A | -2.14257 | 0.001988 | 0.020883 | -3.09206 | Down | DNA topoisomerase II alpha |
| ACTL7A | -2.13887 | 0.002923 | 0.027371 | -2.97574 | Down | actin like 7A |
| OXGR1 | -2.13657 | 0.003157 | 0.028911 | -2.95204 | Down | oxoglutarate receptor 1 |
| MESP2 | -2.13597 | 0.004817 | 0.038713 | -2.81903 | Down | mesoderm posterior bHLH transcription factor 2 |
| CDH22 | -2.13575 | 3.22E-05 | 0.001126 | -4.15707 | Down | cadherin 22 |
| OR10A5 | -2.13555 | 0.00036 | 0.006308 | -3.56815 | Down | olfactory receptor family 10 subfamily A member 5 |
| SPC24 | -2.13072 | 0.002388 | 0.023883 | -3.03725 | Down | SPC24 component of NDC80 kinetochore complex |
| LOC100506422 | -2.12894 | 0.001293 | 0.015506 | -3.21745 | Down | putative deoxyuridine 5'-triphosphate nucleotidohydrolase-like protein FLJ16323 |
| NSUN5P2 | -2.12488 | 0.004023 | 0.034205 | -2.87632 | Down | NSUN5 pseudogene 2 |
| KLHDC8A | -2.12063 | 0.001931 | 0.020472 | -3.10071 | Down | kelch domain containing 8A |
| ANKRD34C | -2.11947 | 0.005215 | 0.040726 | -2.79342 | Down | ankyrin repeat domain 34C |
| OR4C46 | -2.11678 | 0.004308 | 0.035862 | -2.85472 | Down | olfactory receptor family 4 subfamily C member 46 |
| LINC00671 | -2.11267 | 0.000874 | 0.01186 | -3.32835 | Down | long intergenic non-protein coding RNA 671 |
| UCHL1 | -2.10092 | 0.000329 | 0.005946 | -3.59133 | Down | ubiquitin C-terminal hydrolase L1 |
| HLA-C | -2.09812 | 0.001034 | 0.01341 | -3.28109 | Down | major histocompatibility complex, class I, C |
| CLEC4D | -2.09688 | 0.006021 | 0.044768 | -2.74665 | Down | C-type lectin domain family 4 member D |
| FGL1 | -2.09534 | 2.36E-05 | 0.00089 | -4.22771 | Down | fibrinogen like 1 |
| LRRC10B | -2.09389 | 0.001064 | 0.013642 | -3.27304 | Down | leucine rich repeat containing 10B |
| CDKN3 | -2.09092 | 0.0001 | 0.002543 | -3.88942 | Down | cyclin dependent kinase inhibitor 3 |
| TMEM200C | -2.08833 | 0.000653 | 0.009666 | -3.40863 | Down | transmembrane protein 200C |
| KANK4 | -2.07825 | 0.000722 | 0.010375 | -3.38106 | Down | KN motif and ankyrin repeat domains 4 |
| ACTC1 | -2.07805 | 0.001404 | 0.016404 | -3.1939 | Down | actin alpha cardiac muscle 1 |
| COL6A4P2 | -2.06787 | 0.003406 | 0.030366 | -2.92853 | Down | collagen type VI alpha 4 pseudogene 2 |
| VASP | -2.06614 | 9.06E-11 | 5.12E-08 | -6.48188 | Down | vasodilator stimulated phosphoprotein |
| LCN10 | -2.05205 | 0.000462 | 0.00752 | -3.50197 | Down | lipocalin 10 |
| MEG3 | -2.04725 | 1.56E-08 | 3.21E-06 | -5.65483 | Down | maternally expressed 3 |
| CIT | -2.04665 | 0.000351 | 0.00623 | -3.57439 | Down | citron rho-interacting serine/threonine kinase |
| CTNNA3 | -2.03117 | 0.004056 | 0.034415 | -2.87379 | Down | catenin alpha 3 |
| OR52W1 | -2.02704 | 0.004903 | 0.039089 | -2.81336 | Down | olfactory receptor family 52 subfamily W member 1 |
| IL5RA | -2.02549 | 0.006579 | 0.047591 | -2.71742 | Down | interleukin 5 receptor subunit alpha |
| RPL31 | -2.02494 | 0.001538 | 0.017454 | -3.16745 | Down | ribosomal protein L31 |
| CTSE | -2.00792 | 0.005802 | 0.043589 | -2.75877 | Down | cathepsin E |
| SOSTDC1 | -2.00044 | 0.005797 | 0.043583 | -2.75905 | Down | sclerostin domain containing 1 |
| TTC8 | -1.99853 | 2.42E-07 | 2.95E-05 | -5.16352 | Down | tetratricopeptide repeat domain 8 |
| RADIL | -1.98793 | 0.001675 | 0.018469 | -3.14246 | Down | Rap associating with DIL domain |
| TAS2R8 | -1.98705 | 0.004385 | 0.03629 | -2.84903 | Down | taste 2 receptor member 8 |
| OIP5 | -1.97891 | 0.000709 | 0.010265 | -3.38597 | Down | Opa interacting protein 5 |
| OLFM4 | -1.97222 | 0.00062 | 0.009266 | -3.42261 | Down | olfactomedin 4 |
| NDUFA8 | -1.95217 | 1.65E-12 | 1.60E-09 | -7.06142 | Down | NADH:ubiquinoneoxidoreductase subunit A8 |
| TSPAN1 | -1.94046 | 1.70E-05 | 0.000707 | -4.30155 | Down | tetraspanin 1 |
| SDHAP2 | -1.93873 | 2.15E-07 | 2.68E-05 | -5.18585 | Down | succinate dehydrogenase complex flavoprotein subunit A pseudogene 2 |
| MYCN | -1.92871 | 0.006923 | 0.049039 | -2.70052 | Down | MYCN proto-oncogene, bHLH transcription factor |
| GPR141 | -1.91909 | 0.000936 | 0.012505 | -3.30911 | Down | G protein-coupled receptor 141 |
| NTN1 | -1.91802 | 6.50E-07 | 6.32E-05 | -4.9757 | Down | netrin 1 |
| MAGEE2 | -1.90962 | 0.000537 | 0.00834 | -3.46166 | Down | MAGE family member E2 |
| EEF1A2 | -1.90299 | 0.000516 | 0.008113 | -3.47224 | Down | eukaryotic translation elongation factor 1 alpha 2 |
| MAP3K8 | -1.90261 | 0.002553 | 0.024928 | -3.01704 | Down | mitogen-activated protein kinase kinasekinase 8 |
| OR52E6 | -1.89409 | 0.007 | 0.049484 | -2.69686 | Down | olfactory receptor family 52 subfamily E member 6 |
| P2RX6 | -1.8888 | 0.002619 | 0.025432 | -3.00924 | Down | purinergic receptor P2X 6 |
| CTNNA2 | -1.88542 | 0.001368 | 0.016106 | -3.20124 | Down | catenin alpha 2 |
| SNORD17 | -1.87942 | 0.000449 | 0.007397 | -3.50975 | Down | small nucleolar RNA, C/D box 17 |
| DIRC3 | -1.87831 | 0.00023 | 0.004625 | -3.68404 | Down | disrupted in renal carcinoma 3 |
| KIF6 | -1.87185 | 7.20E-06 | 0.000384 | -4.4878 | Down | kinesin family member 6 |
| TTN | -1.86646 | 2.37E-05 | 0.00089 | -4.22669 | Down | titin |
| SAMD3 | -1.85982 | 0.003005 | 0.027939 | -2.96719 | Down | sterile alpha motif domain containing 3 |
| OR6K3 | -1.85822 | 0.00637 | 0.046452 | -2.72811 | Down | olfactory receptor family 6 subfamily K member 3 |
| QRICH2 | -1.85193 | 7.62E-05 | 0.002061 | -3.95612 | Down | glutamine rich 2 |
| RRM2 | -1.85099 | 0.002235 | 0.022764 | -3.05702 | Down | ribonucleotidereductase regulatory subunit M2 |
| BCO2 | -1.84966 | 5.49E-05 | 0.001643 | -4.03358 | Down | beta-carotene oxygenase 2 |
| MUSTN1 | -1.84629 | 0.000187 | 0.003999 | -3.73656 | Down | musculoskeletal, embryonic nuclear protein 1 |
| OR11H2 | -1.84559 | 0.001589 | 0.017817 | -3.1579 | Down | olfactory receptor family 11 subfamily H member 2 |
| ZNF695 | -1.84353 | 0.000466 | 0.007558 | -3.49967 | Down | zinc finger protein 695 |
| SULT1C2 | -1.84327 | 0.000838 | 0.011496 | -3.33987 | Down | sulfotransferase family 1C member 2 |
| GTF2H2C | -1.84144 | 2.42E-08 | 4.62E-06 | -5.57866 | Down | GTF2H2 family member C |
| TK1 | -1.83218 | 0.000127 | 0.003035 | -3.83165 | Down | thymidine kinase 1 |
| GINS2 | -1.83103 | 6.19E-07 | 6.09E-05 | -4.98512 | Down | GINS complex subunit 2 |
| CCR2 | -1.82698 | 0.004504 | 0.036953 | -2.84055 | Down | C-C motif chemokine receptor 2 |
| SHCBP1 | -1.82541 | 0.005054 | 0.039942 | -2.80359 | Down | SHC binding and spindle associated 1 |
| RAB11B-AS1 | -1.8231 | 1.61E-05 | 0.000683 | -4.31271 | Down | RAB11B antisense RNA 1 |
| PRAM1 | -1.81806 | 0.005685 | 0.043083 | -2.76544 | Down | PML-RARA regulated adaptor molecule 1 |
| NCKAP5 | -1.81025 | 9.58E-07 | 8.38E-05 | -4.90001 | Down | NCK associated protein 5 |
| P2RY12 | -1.80448 | 1.20E-05 | 0.000557 | -4.37801 | Down | purinergic receptor P2Y12 |
| CNN1 | -1.80432 | 0.000511 | 0.008054 | -3.47488 | Down | calponin 1 |
| HRC | -1.80167 | 5.89E-05 | 0.001709 | -4.01703 | Down | histidine rich calcium binding protein |
| CYP4Z1 | -1.78642 | 0.005884 | 0.044023 | -2.75418 | Down | cytochrome P450 family 4 subfamily Z member 1 |
| IP6K3 | -1.78353 | 0.00152 | 0.017335 | -3.17089 | Down | inositol hexakisphosphate kinase 3 |
| PTGS2 | -1.7827 | 0.003252 | 0.029509 | -2.94289 | Down | prostaglandin-endoperoxide synthase 2 |
| DEF8 | -1.77864 | 0.000794 | 0.011065 | -3.35471 | Down | differentially expressed in FDCP 8 homolog |
| FOXD2 | -1.77487 | 0.000539 | 0.008367 | -3.46036 | Down | forkhead box D2 |
| APOC1 | -1.77454 | 4.60E-05 | 0.001437 | -4.07517 | Down | apolipoprotein C1 |
| LYZ | -1.77313 | 5.00E-05 | 0.001534 | -4.0555 | Down | lysozyme |
| ITGBL1 | -1.76632 | 1.37E-07 | 1.87E-05 | -5.26957 | Down | integrin subunit beta like 1 |
| GLP1R | -1.7604 | 9.18E-08 | 1.36E-05 | -5.34227 | Down | glucagon like peptide 1 receptor |
| CYP3A5 | -1.75739 | 0.000151 | 0.003436 | -3.79001 | Down | cytochrome P450 family 3 subfamily A member 5 |
| SH3BP2 | -1.75378 | 0.00039 | 0.006665 | -3.54652 | Down | SH3 domain binding protein 2 |
| SNORA47 | -1.75268 | 8.24E-07 | 7.44E-05 | -4.9295 | Down | small nucleolar RNA, H/ACA box 47 |
| NDRG4 | -1.74784 | 0.002093 | 0.02164 | -3.07666 | Down | NDRG family member 4 |
| KLRK1 | -1.74544 | 0.000776 | 0.010938 | -3.36131 | Down | killer cell lectin like receptor K1 |
| CNGA4 | -1.73089 | 0.00257 | 0.025014 | -3.01493 | Down | cyclic nucleotide gated channel subunit alpha 4 |
| APOC2 | -1.71885 | 0.000977 | 0.012856 | -3.29702 | Down | apolipoprotein C2 |
| SCD5 | -1.71546 | 1.24E-07 | 1.74E-05 | -5.28776 | Down | stearoyl-CoA desaturase 5 |
| KRTAP5-1 | -1.71212 | 0.001104 | 0.014013 | -3.26265 | Down | keratin associated protein 5-1 |
| FAM223A | -1.71176 | 0.006595 | 0.047609 | -2.71663 | Down | family with sequence similarity 223 member A |
| ANKRD23 | -1.70813 | 1.69E-06 | 0.000135 | -4.78678 | Down | ankyrin repeat domain 23 |
| HADH | -1.70627 | 3.96E-09 | 1.04E-06 | -5.88592 | Down | hydroxyacyl-CoA dehydrogenase |
| SERPINA3 | -1.70483 | 0.00017 | 0.003704 | -3.76058 | Down | serpin family A member 3 |
| OR52D1 | -1.7042 | 0.004591 | 0.037466 | -2.83438 | Down | olfactory receptor family 52 subfamily D member 1 |
| TMEM178A | -1.70223 | 0.002146 | 0.022047 | -3.06927 | Down | transmembrane protein 178A |
| BMS1P4 | -1.69662 | 4.36E-07 | 4.67E-05 | -5.05261 | Down | BMS1 pseudogene 4 |
| SMAD9 | -1.68648 | 0.006737 | 0.048175 | -2.70957 | Down | SMAD family member 9 |
| PIGP | -1.68357 | 4.36E-08 | 7.24E-06 | -5.47564 | Down | phosphatidylinositol glycan anchor biosynthesis class P |
| GALK2 | -1.68337 | 2.87E-13 | 4.33E-10 | -7.30016 | Down | galactokinase 2 |
| SNORA70E | -1.68291 | 0.001609 | 0.017966 | -3.1543 | Down | small nucleolar RNA, H/ACA box 70E |
| NEFM | -1.6821 | 4.63E-05 | 0.001444 | -4.07361 | Down | neurofilament medium |
| ZNF622 | -1.67707 | 3.95E-08 | 6.81E-06 | -5.49285 | Down | zinc finger protein 622 |
| EFNA4 | -1.67629 | 0.001144 | 0.014391 | -3.25251 | Down | ephrin A4 |
| LRP5L | -1.67397 | 1.35E-05 | 0.000604 | -4.35245 | Down | LDL receptor related protein 5 like |
| MED10 | -1.67292 | 3.27E-06 | 0.000215 | -4.65306 | Down | mediator complex subunit 10 |
| PIR | -1.67257 | 1.57E-07 | 2.11E-05 | -5.24394 | Down | pirin |
| HSD17B3 | -1.67244 | 0.00193 | 0.020472 | -3.10075 | Down | hydroxysteroid 17-beta dehydrogenase 3 |
| MRO | -1.66683 | 2.46E-05 | 0.000915 | -4.21819 | Down | maestro |
| PTGFR | -1.66615 | 4.11E-08 | 6.95E-06 | -5.48615 | Down | prostaglandin F receptor |
| GUSBP11 | -1.66179 | 9.52E-08 | 1.40E-05 | -5.33562 | Down | GUSB pseudogene 11 |
| CDK15 | -1.66068 | 0.000948 | 0.012618 | -3.30537 | Down | cyclin dependent kinase 15 |
| WDR3 | -1.65943 | 1.43E-08 | 3.05E-06 | -5.66959 | Down | WD repeat domain 3 |
| SCRG1 | -1.65505 | 0.001442 | 0.016755 | -3.1861 | Down | stimulator of chondrogenesis 1 |
| NR6A1 | -1.65044 | 0.00046 | 0.007518 | -3.50276 | Down | nuclear receptor subfamily 6 group A member 1 |
| FAM72D | -1.63864 | 0.00183 | 0.019741 | -3.11653 | Down | family with sequence similarity 72 member D |
| ZNF391 | -1.63472 | 8.82E-06 | 0.000445 | -4.44436 | Down | zinc finger protein 391 |
| CFB | -1.63155 | 0.000144 | 0.003338 | -3.80159 | Down | complement factor B |
| LGI2 | -1.62508 | 2.48E-06 | 0.000175 | -4.70977 | Down | leucine rich repeat LGI family member 2 |
| FCHO1 | -1.62484 | 0.005264 | 0.04091 | -2.79039 | Down | FCH and mu domain containing endocytic adaptor 1 |
| LRRC7 | -1.62384 | 0.002558 | 0.024957 | -3.01644 | Down | leucine rich repeat containing 7 |
| NEK6 | -1.62009 | 0.00659 | 0.047609 | -2.71687 | Down | NIMA related kinase 6 |
| LOC648987 | -1.61592 | 0.003007 | 0.02794 | -2.96703 | Down | uncharacterized LOC648987 |
| MMP28 | -1.60978 | 0.000384 | 0.006598 | -3.55064 | Down | matrix metallopeptidase 28 |
| LHFPL3-AS2 | -1.60714 | 0.000745 | 0.010639 | -3.37256 | Down | LHFPL3 antisense RNA 2 |
| AKR1C2 | -1.60408 | 2.37E-05 | 0.00089 | -4.22683 | Down | aldo-ketoreductase family 1 member C2 |
| BUB1B | -1.60351 | 0.005799 | 0.043583 | -2.75895 | Down | BUB1 mitotic checkpoint serine/threonine kinase B |
| LOC100507547 | -1.60229 | 0.000828 | 0.011404 | -3.34317 | Down | uncharacterized LOC100507547 |
| CDH23 | -1.59792 | 0.001165 | 0.01459 | -3.24742 | Down | cadherin related 23 |
| ANXA10 | -1.59619 | 7.10E-06 | 0.000381 | -4.49071 | Down | annexin A10 |
| ADAMTS2 | -1.59391 | 7.57E-07 | 6.88E-05 | -4.94615 | Down | ADAM metallopeptidase with thrombospondin type 1 motif 2 |
| PAPOLB | -1.58767 | 0.005074 | 0.040061 | -2.80231 | Down | poly(A) polymerase beta |
| CDKN1C | -1.58201 | 2.08E-06 | 0.000154 | -4.74542 | Down | cyclin dependent kinase inhibitor 1C |
| ATP2C1 | -1.58082 | 0.001222 | 0.015003 | -3.23363 | Down | ATPase secretory pathway Ca2+ transporting 1 |
| SPAG17 | -1.57901 | 0.003276 | 0.029678 | -2.94057 | Down | sperm associated antigen 17 |
| P2RY1 | -1.57648 | 0.000798 | 0.011091 | -3.35364 | Down | purinergic receptor P2Y1 |
| IGSF11 | -1.57462 | 1.96E-05 | 0.000779 | -4.26992 | Down | immunoglobulin superfamily member 11 |
| COMMD7 | -1.57449 | 0.000354 | 0.006238 | -3.57221 | Down | COMM domain containing 7 |
| LOC440173 | -1.5733 | 0.000203 | 0.004248 | -3.71519 | Down | uncharacterized LOC440173 |
| UBXN10 | -1.56955 | 0.003965 | 0.033807 | -2.8809 | Down | UBX domain protein 10 |
| TSHZ3 | -1.56942 | 3.81E-05 | 0.001252 | -4.11863 | Down | teashirt zinc finger homeobox 3 |
| LRRC39 | -1.56781 | 0.00276 | 0.02628 | -2.99327 | Down | leucine rich repeat containing 39 |
| PDLIM3 | -1.56765 | 3.35E-05 | 0.001154 | -4.14833 | Down | PDZ and LIM domain 3 |
| IFI27L2 | -1.56751 | 0.004728 | 0.038202 | -2.825 | Down | interferon alpha inducible protein 27 like 2 |
| EFCAB12 | -1.56177 | 0.005243 | 0.040847 | -2.79172 | Down | EF-hand calcium binding domain 12 |
| SCGB2A1 | -1.56061 | 0.005011 | 0.039689 | -2.80634 | Down | secretoglobin family 2A member 1 |
| CRYGS | -1.56043 | 0.003935 | 0.033661 | -2.88331 | Down | crystallin gamma S |
| ARHGAP20 | -1.55688 | 0.001308 | 0.015618 | -3.21424 | Down | Rho GTPase activating protein 20 |
| FRMPD1 | -1.54919 | 0.001419 | 0.016541 | -3.19075 | Down | FERM and PDZ domain containing 1 |
| KIF20B | -1.54488 | 0.001915 | 0.020374 | -3.10318 | Down | kinesin family member 20B |
| SELL | -1.54158 | 0.003719 | 0.032311 | -2.90103 | Down | selectin L |
| NEDD8 | -1.54099 | 4.08E-09 | 1.04E-06 | -5.88096 | Down | NEDD8 ubiquitin like modifier |
| RASSF1 | -1.54021 | 0.000352 | 0.006238 | -3.57352 | Down | Ras association domain family member 1 |
| ESR1 | -1.53465 | 0.003504 | 0.030999 | -2.91966 | Down | estrogen receptor 1 |
| ERVK13-1 | -1.52459 | 0.000155 | 0.003502 | -3.78254 | Down | endogenous retrovirus group K13 member 1 |
| HEBP2 | -1.51608 | 1.06E-06 | 9.13E-05 | -4.88012 | Down | heme binding protein 2 |
| ARHGAP22 | -1.51421 | 0.002497 | 0.024632 | -3.02369 | Down | Rho GTPase activating protein 22 |
| TYW1B | -1.51088 | 2.73E-06 | 0.000187 | -4.69036 | Down | tRNA-yW synthesizing protein 1 homolog B |
| AGAP2 | -1.51064 | 0.003808 | 0.03285 | -2.89366 | Down | ArfGAP with GTPase domain, ankyrin repeat and PH domain 2 |
| CYP4F12 | -1.5094 | 0.004865 | 0.038909 | -2.81584 | Down | cytochrome P450 family 4 subfamily F member 12 |
| RASD2 | -1.50824 | 1.28E-06 | 0.000107 | -4.84244 | Down | RASD family member 2 |
| SLC26A7 | -1.50389 | 0.002177 | 0.022312 | -3.06497 | Down | solute carrier family 26 member 7 |
| LY6G5B | -1.49949 | 0.002268 | 0.023028 | -3.05265 | Down | lymphocyte antigen 6 family member G5B |
| TFCP2L1 | -1.49843 | 5.31E-06 | 0.000312 | -4.55219 | Down | transcription factor CP2 like 1 |
| CSF2RA | -1.49594 | 0.002137 | 0.02198 | -3.07048 | Down | colony stimulating factor 2 receptor subunit alpha |
| TDH | -1.49341 | 0.001129 | 0.014269 | -3.25632 | Down | L-threonine dehydrogenase (pseudogene) |
| RELT | -1.49125 | 0.001848 | 0.019864 | -3.11363 | Down | RELT TNF receptor |
| MAP1A | -1.48562 | 0.001649 | 0.018241 | -3.14714 | Down | microtubule associated protein 1A |
| LOXL1 | -1.48506 | 0.000251 | 0.004917 | -3.66139 | Down | lysyl oxidase like 1 |
| KIF18A | -1.48239 | 0.001571 | 0.017708 | -3.16123 | Down | kinesin family member 18A |
| B3GNT3 | -1.47982 | 0.002083 | 0.021574 | -3.07809 | Down | UDP-GlcNAc:betaGal beta-1,3-N-acetylglucosaminyltransferase 3 |
| LOC646214 | -1.47392 | 0.001568 | 0.017688 | -3.16181 | Down | p21 (RAC1) activated kinase 2 pseudogene |
| PRICKLE4 | -1.47244 | 0.003718 | 0.032311 | -2.90114 | Down | prickle planar cell polarity protein 4 |
| MYO1F | -1.47133 | 0.000544 | 0.008416 | -3.45796 | Down | myosin IF |
| JMJD6 | -1.47043 | 4.70E-07 | 4.99E-05 | -5.03799 | Down | jumonji domain containing 6, arginine demethylase and lysine hydroxylase |
| TXNDC9 | -1.46925 | 6.09E-13 | 7.86E-10 | -7.19855 | Down | thioredoxin domain containing 9 |
| STXBP5-AS1 | -1.4673 | 0.002667 | 0.025755 | -3.00372 | Down | STXBP5 antisense RNA 1 |
| LUC7L | -1.46492 | 1.72E-06 | 0.000136 | -4.78434 | Down | LUC7 like |
| ARG2 | -1.46353 | 0.002923 | 0.027371 | -2.97571 | Down | arginase 2 |
| POU5F1 | -1.45988 | 0.006916 | 0.049011 | -2.70084 | Down | POU class 5 homeobox 1 |
| ARHGAP9 | -1.45129 | 0.002566 | 0.025001 | -3.01541 | Down | Rho GTPase activating protein 9 |
| LINC00239 | -1.45125 | 0.001024 | 0.013326 | -3.28388 | Down | long intergenic non-protein coding RNA 239 |
| MS4A7 | -1.44992 | 2.59E-05 | 0.000948 | -4.20697 | Down | membrane spanning 4-domains A7 |
| ROBO1 | -1.44661 | 0.000429 | 0.007144 | -3.52164 | Down | roundabout guidance receptor 1 |
| TRIM66 | -1.44341 | 5.54E-06 | 0.000322 | -4.5431 | Down | tripartite motif containing 66 |
| ALOX5 | -1.43679 | 1.24E-07 | 1.74E-05 | -5.28802 | Down | arachidonate 5-lipoxygenase |
| GPX2 | -1.43654 | 0.000227 | 0.004581 | -3.68729 | Down | glutathione peroxidase 2 |
| EXOSC8 | -1.43535 | 3.46E-10 | 1.40E-07 | -6.27648 | Down | exosome component 8 |
| NUSAP1 | -1.43456 | 0.001488 | 0.017083 | -3.17698 | Down | nucleolar and spindle associated protein 1 |
| ANLN | -1.43239 | 0.004239 | 0.035455 | -2.8598 | Down | anillin actin binding protein |
| CDK1 | -1.43222 | 0.001258 | 0.015231 | -3.22537 | Down | cyclin dependent kinase 1 |
| SELPLG | -1.43155 | 0.000579 | 0.008821 | -3.44138 | Down | selectin P ligand |
| GREB1 | -1.42927 | 3.36E-07 | 3.75E-05 | -5.10196 | Down | growth regulating estrogen receptor binding 1 |
| MYH11 | -1.42608 | 0.004478 | 0.036813 | -2.84233 | Down | myosin heavy chain 11 |
| ABCB1 | -1.42508 | 0.001795 | 0.019432 | -3.12223 | Down | ATP binding cassette subfamily B member 1 |
| CHCHD1 | -1.41714 | 0.001135 | 0.014326 | -3.25479 | Down | coiled-coil-helix-coiled-coil-helix domain containing 1 |
| PDE1C | -1.41678 | 0.001008 | 0.013153 | -3.28817 | Down | phosphodiesterase 1C |
| DLG2 | -1.41507 | 6.13E-05 | 0.001753 | -4.00758 | Down | discs large MAGUK scaffold protein 2 |
| SNORA57 | -1.41264 | 0.000168 | 0.00368 | -3.76338 | Down | small nucleolar RNA, H/ACA box 57 |
| COQ5 | -1.41257 | 1.02E-06 | 8.87E-05 | -4.88694 | Down | coenzyme Q5, methyltransferase |
| CYP2J2 | -1.40784 | 7.09E-05 | 0.001948 | -3.97338 | Down | cytochrome P450 family 2 subfamily J member 2 |
| TEKT2 | -1.40714 | 0.001087 | 0.013854 | -3.26686 | Down | tektin 2 |
| CPA4 | -1.39844 | 0.002227 | 0.022694 | -3.05821 | Down | carboxypeptidase A4 |
| OR4F3 | -1.39842 | 0.006622 | 0.04769 | -2.71528 | Down | olfactory receptor family 4 subfamily F member 3 |
| ADAMTS10 | -1.39793 | 0.00084 | 0.011516 | -3.33917 | Down | ADAM metallopeptidase with thrombospondin type 1 motif 10 |
| VAV3 | -1.39763 | 3.65E-06 | 0.000231 | -4.63055 | Down | vav guanine nucleotide exchange factor 3 |
| HSD17B7P2 | -1.39759 | 1.68E-05 | 0.000705 | -4.30357 | Down | hydroxysteroid 17-beta dehydrogenase 7 pseudogene 2 |
| TYMS | -1.39182 | 8.67E-05 | 0.00227 | -3.92507 | Down | thymidylatesynthetase |
| SCN1B | -1.39167 | 3.70E-06 | 0.000233 | -4.62739 | Down | sodium voltage-gated channel beta subunit 1 |
| LAT | -1.38953 | 0.001091 | 0.013873 | -3.26588 | Down | linker for activation of T cells |
| SNORA71B | -1.3858 | 0.001448 | 0.016813 | -3.18491 | Down | small nucleolar RNA, H/ACA box 71B |
| SMIM5 | -1.38397 | 0.002396 | 0.023909 | -3.03617 | Down | small integral membrane protein 5 |
| MATN3 | -1.38264 | 0.00087 | 0.011855 | -3.32948 | Down | matrilin 3 |
| RNASE2 | -1.37954 | 0.004946 | 0.039334 | -2.81051 | Down | ribonuclease A family member 2 |
| SWT1 | -1.36623 | 1.40E-05 | 0.000618 | -4.3442 | Down | SWT1 RNA endoribonuclease homolog |
| LAMB3 | -1.36487 | 0.002986 | 0.027804 | -2.96915 | Down | laminin subunit beta 3 |
| LINC00244 | -1.36128 | 0.005216 | 0.040726 | -2.79338 | Down | long intergenic non-protein coding RNA 244 |
| PRODH | -1.36073 | 0.001733 | 0.018929 | -3.1326 | Down | proline dehydrogenase 1 |
| SRD5A1 | -1.35922 | 4.03E-06 | 0.000249 | -4.60986 | Down | steroid 5 alpha-reductase 1 |
| SRPX2 | -1.35844 | 5.74E-07 | 5.74E-05 | -4.99961 | Down | sushi repeat containing protein X-linked 2 |
| APOBEC2 | -1.35451 | 1.34E-06 | 0.000111 | -4.8333 | Down | apolipoprotein B mRNA editing enzyme catalytic subunit 2 |
| PKDREJ | -1.35249 | 0.002568 | 0.025007 | -3.01517 | Down | polycystin family receptor for egg jelly |
| SGIP1 | -1.35053 | 0.000715 | 0.010307 | -3.38374 | Down | SH3GL interacting endocytic adaptor 1 |
| NAPSB | -1.34741 | 0.001225 | 0.015005 | -3.23311 | Down | napsin B aspartic peptidase, pseudogene |
| MBL1P | -1.34188 | 0.004945 | 0.039334 | -2.81059 | Down | mannose binding lectin 1, pseudogene |
| SLC7A7 | -1.33735 | 1.58E-05 | 0.000674 | -4.31659 | Down | solute carrier family 7 member 7 |
| GLG1 | -1.33647 | 4.71E-07 | 4.99E-05 | -5.03762 | Down | golgi glycoprotein 1 |
| SNORA10 | -1.33039 | 0.001471 | 0.017006 | -3.18032 | Down | small nucleolar RNA, H/ACA box 10 |
| TPM2 | -1.32665 | 6.85E-08 | 1.04E-05 | -5.39512 | Down | tropomyosin 2 |
| MEG8 | -1.32203 | 1.68E-07 | 2.23E-05 | -5.23175 | Down | maternally expressed 8, small nucleolar RNA host gene |
| ASB16 | -1.32095 | 5.04E-05 | 0.001536 | -4.05368 | Down | ankyrin repeat and SOCS box containing 16 |
| RAD51AP1 | -1.31844 | 0.001646 | 0.018238 | -3.14755 | Down | RAD51 associated protein 1 |
| TBC1D3 | -1.31758 | 0.000265 | 0.005132 | -3.6469 | Down | TBC1 domain family member 3 |
| SIX2 | -1.31048 | 0.000344 | 0.006141 | -3.57942 | Down | SIX homeobox 2 |
| TBC1D3F | -1.30768 | 0.000476 | 0.007661 | -3.49393 | Down | TBC1 domain family member 3F |
| SAMHD1 | -1.30624 | 3.02E-08 | 5.40E-06 | -5.54048 | Down | SAM and HD domain containing deoxynucleoside triphosphate triphosphohydrolase 1 |
| FAM227A | -1.3019 | 0.001032 | 0.01339 | -3.28173 | Down | family with sequence similarity 227 member A |
| LOC100506123 | -1.30135 | 0.001802 | 0.019495 | -3.12109 | Down | uncharacterized LOC100506123 |
| SERTAD4-AS1 | -1.30081 | 0.003311 | 0.029845 | -2.93729 | Down | SERTAD4 antisense RNA 1 |
